# Conformational Locking of the Geometry in Photoluminescent Cyclometalated N^C^N Ni(II) Complexes

**DOI:** 10.3390/molecules30091901

**Published:** 2025-04-24

**Authors:** Maryam Niazi, Iván Maisuls, Lukas A. Mai, Sascha A. Schäfer, Alex Oster, Lukas Santiago Diaz, Dirk M. Guldi, Nikos L. Doltsinis, Cristian A. Strassert, Axel Klein

**Affiliations:** 1 Department of Chemistry and Biochemistry, Institute for Inorganic and Materials Chemistry, Faculty for Mathematics and Natural Sciences, University of Cologne, Greinstraße 6, D-50939 Köln, Germany; mniazi1@smail.uni-koeln.de (M.N.); sascha.schaefer@uni-koeln.de (S.A.S.); 2 Institut für Anorganische und Analytische Chemie, Universität Münster, Corrensstraße 28/30, D-48149 Münster, Germany; maisuls@uni-muenster.de; 3 CiMIC, CeNTech, Heisenbergstraße 11, D-48149 Münster, Germany; 4 Department of Chemistry and Pharmacy & Interdisciplinary Center for Molecular Materials (ICMM), Friedrich-Alexander-University Erlangen-Nuremberg, Egerlandstraße 3, D-91058 Erlangen, Germany; lukas.mai@fau.de (L.A.M.); lukas.santiago@fau.de (L.S.D.); dirk.guldi@fau.de (D.M.G.); 5 Institut für Festkörpertheorie and Center for Multiscale Theory and Computation, Universität Münster, Wilhelm-Klemm-Straße 10, D-48149 Münster, Germany; oster.alex@uni-muenster.de

**Keywords:** nickel(II), cyclometalation, electrochemistry, photoluminescence, DFT calculations

## Abstract

In our research aimed at replacing precious transition metals like platinum with abundant base metals such as nickel for efficient triplet emitters, we synthesized and studied Ni(II) complexes [Ni(L^NHR^)Cl]. These complexes containing the N^C^N cyclometalating dipyridyl-phenide ligand, equipped with pending H-bonding amine groups (NH(C₆H₅) (L^NHPh^) and NH(C₆H₅CH₂), ClL^NHBn^). Molecular structures determined from experimental X-ray diffractometry and density functional theory (DFT) calculations in the ground state showed marked deviation of the Cl^−^ coligand (ancillary ligand) from the ideal planar coordination, with τ_4_ values of 0.35 and 0.33, respectively, along with hydrogen bonding interactions of the ligand NH function with the Cl^−^ coligand. The complexes exhibit long-wavelength absorption bands at approximately 425 nm in solution, with the experimental spectra being accurately reproduced through time-dependent density functional theory (TD-DFT) calculations. Vibrationally structured emission profiles and steady-state photoluminescence quantum yields of 30% for [Ni(L^NHPh^)Cl] and 40% for [Ni(L^NHBn^)Cl] (along with dual excited state lifetimes in the ns and in the ms range) were found in frozen 2-methyl-tetrahydrofuran (2MeTHF) glassy matrices at 77 K. Furthermore, within a poly(methyl methacrylate) matrix, the complexes showed emission bands centered at around 550 nm within a temperature range from 6 K to 300 K with lifetimes similar to 77 K. Based on TD-DFT potential scans along the metal–ligand (Ni–N) coordinate, we found that in a rigid environment that restricts the geometry to the Franck-Condon region, either the triplet *T*_5_ or the singlet *S*_4_ state could contribute to the photoluminescence.

## 1. Introduction

Organometallic transition metal complexes with efficient phosphorescence at ambient temperature are the subject of academic and technological interest, promising applications in illumination, display technology, sensors, therapeutic agents, and biological probes [1,2,3,4,5,6,7,8]. High quantum efficiency of such materials is attributed to strong spin–orbit coupling (SOC) which facilitates triplet (electro)luminescence [9,10]. However, many transition metal complexes are scarcely emissive at room temperature, often due to efficient non-radiative deactivation of their excited metal-centered (MC) (i.e., π/d–d*) states [10,11,12]. Cyclometalated heteroaromatic ligands represent a promising strategy for creating effective emitters. The strong ligand field of the cyclometalated carbanion raises the energy of the MC states, thereby impeding the population of these non-emissive, or “dark” states [1,2,3,6,7,8]. Additionally, the rigidity introduced by these ligands prevents significant distortion of the excited state compared to the electronic ground state. The rigidity of both the organic ligand and the coordination geometry around the metal is crucial for generating phosphorescent complexes [2,7,8,13]. This approach has led to the development of numerous cyclometalated d^8^-configured Pt(II) and related Pd(II) complexes, which are emissive from either triplet states of primarily ^3^π–π* character or from ^3^MLCT (metal-to-ligand charge transfer) states (i.e., ^3^d–π* configurations) as well as admixtures thereof [2,13,14,15,16,17,18,19,20,21].

When comparing isoleptic Pt(II), Pd(II), and Ni(II) complexes as potential luminophores [22,23,24,25,26], it is clear that efficient triplet emitters can be easily obtained with the heavy Pt(II) center, with literally hundreds of examples reported containing rigid, heteroaromatic tridentate or tetradentate ligands [1,2,13,14,27,28,29,30]. The number of luminescent Pd(II) complexes is still significantly high despite the lower spin–orbit coupling of Pd (1412 cm^−1^ compared with 4052 cm^−1^ for Pt) [31]. The smaller ligand-field splitting of Pd(II) can be compensated by judicious ligand design (rigidity, donor strength, and chromophores) [2,4,5,18,19,20,21,22,23,25,27]. The ultimate challenge, however, lies with luminescent Ni(II) derivatives, which usually suffer from the thermal accessibility of the dark (π/d–d*) states as well as from their poorer synthetic availability and reduced stability compared to the heavier homologues [32,33,34,35,36,37,38,39,40,41,42]. Spin–orbit coupling of Ni (630 cm^−1^) is only a fraction of that of Pt, and the easy cyclometalation chemistry established for Pt(II) and Pd(II) complexes over the past 30 years was not available for Ni(II) for a long time [22].

**Scheme 1:**
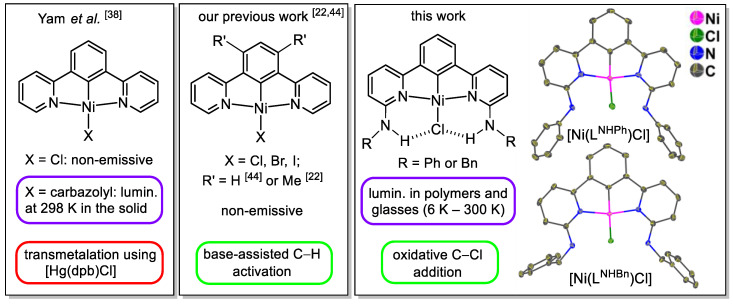
The previously reported complexes [Ni(dpb)(carbazolate)] and [Ni(dpb)X] (Hdpb = 1,3-bis(2-pyridyl)benzene), and the herein reported Ni(II) complexes [Ni(L^NHPh^)Cl] and [Ni(L^NHBn^)Cl].

Recently, the first phosphorescent cyclometalated Ni(II) complex of the type [Ni(dpb)(carbazolate)] (Hdpb = 1,3-bis(2-pyridyl)benzene; Scheme 1) was reported by Yam et al. [38], showing weak photoluminescence in the solid state at ambient temperature. At 77 K, an emission band peaking at 468 nm was observed with a vibrational progression of around 1300 to 1400 cm^−1^, corresponding to the heteroaromatic νC = N and νC = C vibrational modes of the N^C^N ligand and an excited state lifetime in the sub-microsecond range was obtained. Based on this result, the emission band was tentatively assigned to a metal-perturbed ligand-centered (^3^LC or π–π*) excited state [38]. The synthesis of this complex requires the use of an Hg-intermediate [Hg(dpb)Cl] for a transmetalation reaction [38]. In contrast, for the C^C^C coordinated complexes [Ni(*C^C^C*)(MeCN)]X (X = PF_6_, BF_4_, BPh_4_, SO_3_CF_3_) with *N*-butyl-imidazolydene as pending C* groups, a ligand-centered fluorescence was reported [39]. Very recently, dinuclear derivatives [Ni(*C^C^C*)(L)](PF_6_) (L = anionic N,N bridging ligands bis(phenyl-λ^2^-azaneyl)methanide or pyrido[2,3-b]indol-9-ide) were reported to show near-infrared photoluminescence from metal-metal-to-ligand charge-transfer excited states [40]. When using the same NHC ligand and bulky isonitrile ligands, ^3^MLCT-based luminescence with up to 48 ps lifetime was observed [32]. Moreover, a Ni(II) complex with a phenazine-based O^N^N^O ligand showed fluorescence from a coordination-induced spin state switch [35]. Efficient thermal quenching was very recently reported for a cyclometalated Ni(II) complex of a macrocyclic N^N^C^N tripyrrin-phenyldiether ligand, possessing mixed ligand-centered (^3^LC)/metal-to-ligand charge transfer (^3^MLCT) excited states [33]. Very recently, the picosecond decay of the photoexcited states of the two Ni(II) chlorido complexes with the above mentioned *C^C^C* and dpb ligands was studied using UV-vis transient absorption (TA) spectroscopy and compared with their Pt(II) analogs [42]. This study showed that both complexes display initial TA features of ^1^MLCT character yielding rapidly (less than 1 ps) to excited states of ^3^MC character which decay within about 10 to 40 ps. No evidence was found for ^3^MLCT or ^3^LC states for the Ni complexes, which are involved in the photoluminescence of their Pt derivatives [42]. Very recently, we reported on Ni(II)(dpb) complexes with chlorido, azido, and triazolato coligands. The triazolato derivative carrying a coumarin substituent on the triazolato coligand showed phosphorescence from the triplet *T*_5_ state in frozen glassy matrices [43].

Herein, we report the synthesis of two cyclometalated Ni(II) complexes [Ni(L^NHR^)Cl] with bis-3-NHR substituted dpb ligands (R = phenyl (L^NHPh^) or benzyl (L^NHBn^). The introduction of the NHR substituents aims to rigidify and distort the coordination geometries through NH^...^Cl^...^NH hydrogen bonds (Scheme 1). This approach follows the relatively new idea that the rigidity of the coordination around the metal can be enhanced by non-covalent interactions in the so-called second coordination sphere, thereby improving the photoluminescence (PL) efficiency i.e., the corresponding quantum yields *Φ*_PL_ [30]. Following this approach, we also recently reported on the Pt(II) and Pd(II) complexes of the same ligands. We found large distortions of the square planar geometry around the metal centers with τ_4_ values from 0.24 to almost 0.3 [44]. However, the photoluminescence properties, with moderate quantum yields (*Φ*_PL_) of around 0.2 at ambient temperature in solution (under Ar) and around 0.6 at 77 K in frozen glassy matrices for the two Pt(II) complexes were not markedly boosted compared with Pt(II) complexes of sterically non-bulky dipyridyl(phen-2-ide) ligands. The complexes [Pd(L^NHPh^)Cl] and [Pd(L^NHBn^)Cl] were not luminescent at ambient temperature and showed quantum yields of around 0.08 at 77 K in frozen glassy matrices [44]. The herein studied Ni(II) complexes show partially structured emission bands in glassy frozen 2-Me-THF matrices at 77 K and in poly(methyl methacrylate) (PMMA) matrix in the temperature range between 6 K and 300 K, with *Φ*_PL_ values exceeding 30% in frozen glassy matrices. Detailed photophysical and electrochemical measurements as well as (TD-)DFT calculations provided excellent insights into the electronics of the two luminescent [Ni(L^NHR^)Cl] complexes.

## 2. Results and Discussion

### 2.1. Syntheses and Characterization

The two ligand precursors, ClL^NHPh^ and ClL^NHBn^, were synthesized through the optimization of established methods, including Suzuki cross-coupling reactions, with a yield of 65% (details in the syntheses part in the Supplementary Materials (SM) and Figures S1–S4, SM). The two Ni(II) complexes were obtained from an oxidative addition reaction of [Ni(COD)_2_] (COD = 1,5-cyclooctadiene) with the chlorinated ligand precursors, ClL^NHPh^ and ClL^NHBn^, in 60 and 67% yields, respectively. The complexes were obtained as orange microcrystalline powders and were analyzed using ^1^H nuclear magnetic resonance (NMR) spectroscopy, high-resolution electrospray mass spectrometry (HR-ESI-MS), and elemental analysis (CHN) (details on syntheses and analytics in the SM and Figures S5–S9). The two complexes are insoluble in non-polar solvents but are soluble in polar solvents, though they must be handled under the exclusion of air and moisture (Figures S9 and S10).

### 2.2. Experimental Crystal Structures from X-Ray Diffractometry and DFT-Calculated Molecular Structures

Both complexes crystallize in the monoclinic space group *P*2_1_/n (Figures S11–S14, data in Tables S1–S3), similarly to [Ni(dpb)X] with X = Cl, Br, and I [45]. In both crystal structures, a characteristic head-to-tail stacking of the molecules was found (Figures S11–S14), very similar to those observed for the previously reported [M(dpb)X] or [M(phbpy)Br] derivatives with M = Ni, Pd, and Pt [22,38,44,45,46,47,48,49]. The shortest π-stacking interaction in [Ni(L^NHPh^)Cl] occurs between the pyridine units of two neighboring molecules with an angle of 8.159°, a centroid–centroid distance of 3.613 Å, and shift distance of 1.242 Å (Figure S13). Metallophilic Ni^…^Ni interactions, as recently proposed for similar [Ni(N^C^N)(L)] complexes (L = thiophenol or thiadiazole) [50], were not found in our experimental structures.

**Figure 1:**
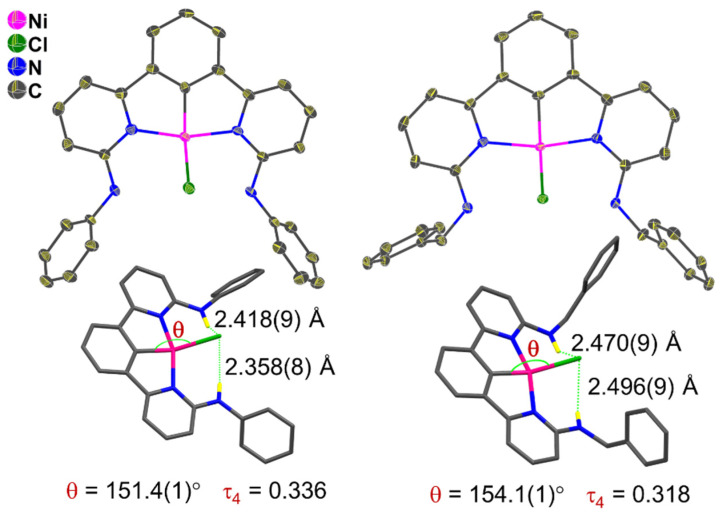
Molecular structures of the complexes [Ni(L^NHR^)Cl] (N^C^N = L^NHPh^, top left and L^NHBn^, top right) from X-ray diffraction studies on single crystals (top, 50% displacement ellipsoids) and their side-on views featuring the deviation τ from the square planar coordination (bottom).

The molecular structures of the two Ni(II) complexes are overall very similar to the previously reported Ni(II) dpb complexes in terms of bond length and angles [38,45]. However, both complexes show C–Ni‒Cl angles of around 150° instead of 180° (Figure 1). Intramolecular non-symmetric Ni‒Cl^...^H‒N hydrogen bonds were found ranging from about 2.36 to 2.49 Å with angles of around 123 to 138°, which represent medium-strong hydrogen bonds (Figure 1, Tables S2 and S3) [51]. In contrast to our initial idea to govern the complex geometry through intramolecular hydrogen bonding, the found hydrogen bonds are not the cause of the geometric distortion of the two complexes, as shown by the comparative geometry optimization with F or H (or the replacement of the NHR function by OMe). Geometry optimization using density functional theory (DFT) at the TPSSh/6-31G(d)/LanL2DZ level (Table S2), gave a very good agreement between experimental and calculated metrics (Figure S15, Table S2, the resulting *xyz* coordinates of the optimized structures are presented in Tables S21 and S22). This is in keeping with many studies using the TPSSh functional for square planar d^8^-configured Ni(II), Pd(II), and Pt(II) complexes [22,24,35,44,45,46,52]. In parallel, we also tried alternative settings for the DFT geometry optimizations, in particular the combinations TPSSh/def2-TZVP/CPCM(THF), B3LYP/6-31G(d)/def2-TZVP/CPCM(THF), TPSSh/6-31G(d)/def2-TZVP/CPCM(THF), and PBE0/SDD. However, these methods gave very similar bond parameters (Table S4). This allowed us to use the TPSSh functional as a consistent setting as previously used for the DFT and time-dependent DFT (TD-DFT) calculations on the Pt and Pd derivatives of [Ni(L^NHPh^)Cl] and [Ni(L^NHBn^)Cl] [44]. On the other hand, for our detailed TD-DFT structural distortion calculations with the Tamm–Dancoff approximation (TDA), and the radiative lifetime calculations, the PBE0 exchange–correlation functional was used.

The most remarkable feature is the marked deviation of the Cl coligand from the square planar coordination plane with τ_4_ values of 0.336 for L^NHPh^ and 0.318 for L^NHBn^ (Figure 1). Correspondingly, the DFT-calculated C–M–Cl angle shows a pronounced energy minimum at 150°, since wider angles are penalized when moving towards planarity (Figure S23). We also calculated the hypothetical complexes [Ni(L^NHPh^)F] and [Ni(L^NHPh^)H] having far smaller coligands, which results in very reduced τ_4_ values of 0.19 and 0.11, respectively. Still, they are not completely planar (Figure S16, Table S5). Evidently, the rigid RNH‒N^C^N‒NHR (R = Ph or Bn) ligand scaffold does not provide enough space to accommodate even the smallest anionic coligand H^‒^. When replacing the NHR groups through OMe substituents on the N^C^N scaffold, the steric bulk of the binding pocket for the coligand even increased resulting in a higher τ_4_ value of 0.411 for the DFT-optimized [Ni(L^OMe^)Cl] complex (Figure S16, Table S5). This occurs while the τ_4_ value for the nearly completely planar complex [Ni(dpb)Cl] is 0.116, based on single crystal data [38,45]. Similarly, the τ_4_ value for [Ni(triazolato^Coumarin,COOCH3^)(dpb)] is 0.107 [43].

For the recently reported Pt(II) and Pd(II) complexes [M(L^NHPh^)Cl] and [M(L^NHBn^)Cl] (M = Pt or Pd), we found τ_4_ values ranging from 0.239 to 0.296 [44], which are markedly smaller than those found for the Ni derivatives. However, the main reason for the strong deviation of the Cl^–^ coligand from the plane is the same in the Pt, Pd, and Ni complexes of these two chelate ligands.

### 2.3. Electrochemistry and DFT-Calculated Frontier Molecular Orbitals

**Figure 2:**
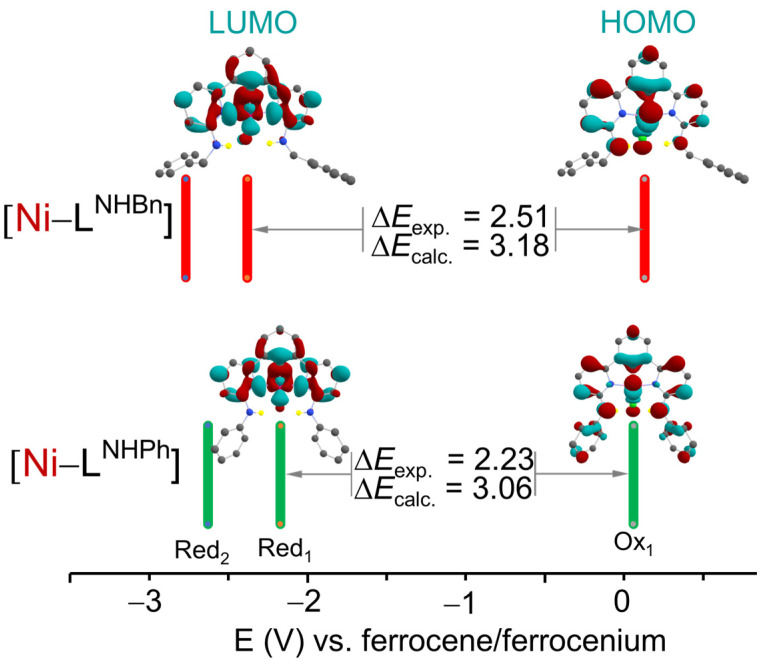
Selected experimental redox potentials (bars) and HOMO–LUMO gaps (Δ*E*_exp._) from cyclic voltammetry along with DFT-calculated orbital compositions and energies of the HOMO and LUMO and the corresponding HOMO–LUMO gap (Δ*E*_calc_.) for the Ni complexes. HOMO = highest occupied molecular orbital; LUMO = lowest unoccupied molecular orbital.

At a first glance, the cyclic voltammograms for the two complexes are very similar, each exhibiting two reduction peaks in the range of −2.2 to −2.8 V vs. the ferrocene/ferrocenium couple, and one oxidation wave at very similar potentials, slightly above 0 V (Figure 2 and Figure S17, Table S6). The DFT-calculated lowest unoccupied molecular orbitals (LUMO) are centered on the N^C^N ligand core with some contributions from Ni (d_x2–y2_) and Cl for both complexes (for details, see Figure S18 and Tables S7 and S9).

For the highest occupied molecular orbitals (HOMOs), the calculations show contributions of the pending phenyl cores for the L^NHPh^ complex, while this is not the case for the L^NHBn^ derivative (Figure S18, Tables S7 and S9) and all other contributions are very similar. This might explain the slightly higher oxidation potential for the L^NHBn^ complex.

### 2.4. UV-Vis Absorption Spectra and TD-DFT-Calculated Electronic Transitions

**Figure 3:**
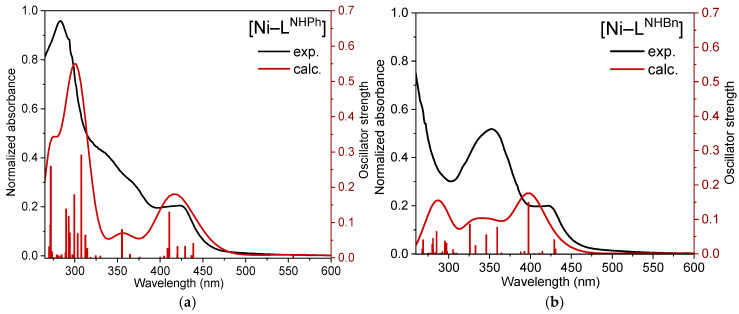
UV-vis absorption spectra of the complexes [Ni(N^C^N)Cl] (N^C^N = L^NHPh^ (**a**) and L^NHBn^ (**b**) in THF solution at 298 K with calculated time-dependent DFT (TD-DFT) transitions (spectrum as line and sticks for individual transitions, in red).

The intense UV absorption bands of the complexes, peaking at 250 nm (L^NHBn^) and 283 (L^NHPh^) nm, also occur for the ligand precursors (Figure 3, Figures S19 and S20, Table S11) and are assigned to transitions into singlet π–π* (LC) states. The bands in the visible range with maxima at 340 nm (L^NHBn^) and 368 nm (L^NHPh^) as well as the long-wavelength bands at around 425 nm, are similar to those previously observed for the Pt(II) and Pd(II) derivatives [44], with the long-wavelength energies of the Ni being the lowest in the series Ni < Pt < Pd. For the recently reported [Ni(dpb)(triazolato)] complexes, similar long-wavelength absorptions at 407 for the derivative with two COOMe substituents on the triazolo coligand [Ni(triazolato^COOCH3,COOCH3^)(dpb)] and 410 nm for the coumarin/COOMe substituted derivative [Ni(triazolato^Coumarin,COOCH3^)(dpb)] were reported [43]. In analogy to these Ni(II)(dpb) complexes, and the Pt(II) and Pd(II) derivatives containing L^NHBn^ or L^NHPh^ ligands, we can attribute the long-wavelength bands of the two Ni(II) complexes to transitions into mixed LC/metal-to-ligand charge transfer (MLCT) singlet states.

The TD-DFT calculated transitions match the observed absorption bands (Figure 3, Figures S19 and S20, Tables S11–S13) for both the UV range and in the visible. The appearance of the long-wavelength bands is very similar to those of other reported Ni, Pd, and Pt complexes of dpb derivatives [22,43,44,45,53,54,55,56,57,58,59], aligning with our assignment.

### 2.5. Photophysical Properties at Low Temperature and in Poly(methyl methacylate) (PMMA) Matrices

**Figure 4:**
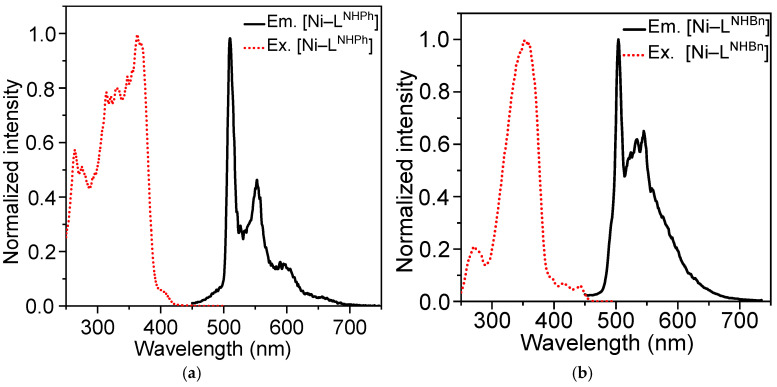
Photoluminescence excitation (red, *λ*_em_ = 525 nm) and emission (black, *λ*_ex_ = 350 nm) spectra of the complexes [Ni(N^C^N)Cl] at 77 K in a glassy matrix of frozen 2-methyltetrahydrofuran (2MeTHF). (**a**): [Ni(L^NHPh^)Cl]; (**b**): [Ni(L^NHBn^)Cl].

While the two Ni(II) complexes did not show photoluminescence in liquid solutions at RT, within a frozen 2-methyltetrahydrofuran (2MeTHF) glassy matrix at 77 K, vibrationally structured emission bands were recorded (Figure 4) with quantum yields of 30% for [Ni(L^NHPh^)Cl] and 40% for [Ni(L^NHBn^)Cl] (data in Table 1).

The bands look very similar to those recorded for the Pt(II) and Pd(II) derivatives [44]. The excitation spectra show broad intense bands peaking at around 350 nm, coincident with intense bands in the absorption spectra, while very small long-wavelength features in the excitation spectrum resemble the long-wavelength absorptions (Figure 3).

We observed lifetime components in both the ms and ns range (Table 1). The long lifetimes of 648 ms for [Ni(L^NHPh^)Cl] and 178 ms for [Ni(L^NHBn^)Cl] were far longer than those for the Pt(II) derivatives (about 30 µs) and markedly longer than those found for the Pd(II) analogs (540 µs) [43]. The short lifetimes lie around 3.5 ns for both complexes, while the Pt(II) and Pd(II) analogs did not show such short-lived components.

Measuring the Ni(II) complexes incorporated into poly(methyl methacrylate, PMMA) films in the temperature range 6 K to 300 K, we observed broad emission bands peaking at around 540 nm and a temperature dependence in both structuring of the emission bands and lifetimes (Figure 5, Figures S27–S48, and Table 1). Increasing the temperature resulted in a less vibrationally structured emission profile and a shortening of the lifetimes for both complexes (Figure 5 and Table 1). This is true for both the long and the short components of the lifetimes. The quantum yields dropped to 0.03 and 0.04 at 300 K in PMMA.

To ensure that the emission does not originate from residual ligand, the ligand precursors ClL^NHBn^ and ClL^NHPh^, as well as the protonated ligands HL^NHBn^ and HL^NHPh^ were measured at 77 K. Neither the emission spectra nor the lifetimes resemble those of the complexes (Figure S49, Table S14), which makes us confident that both the long and short lifetime components of the emission arise from the Ni(II) complexes.

Remarkably, even at 300 K, lifetimes in the ms range (143 ms for [Ni(L^NHPh^)Cl] and 1.05 ms for [Ni(L^NHBn^)Cl], along with the nanosecond components) were achieved. The differences between the complexes can be attributed to the higher deactivation rates associated with the less rigid benzyl-substituted ligand. This behavior was also observed at 77 K.

For the very recently reported [Ni(triazolato^Coumarin,COOCH3^)(dpb)] complex, a very similar, partially structured emission with the maximum at 472 nm was observed in glassy frozen THF matrices at 77 K when irradiated at 320 nm. At 298 K, the complex showed a broad emission band peaking at 413 nm (λ_exc_ = 335 nm). Remarkably, the [Ni(triazolato^COOCH3,COOCH3^)(dpb)] did not show any emission at 298 or 77 K [43]. The lifetime for the [Ni(triazolato^Coumarin,COOCH3^)(dpb)] complex was measured to about 148 µs [43].

### 2.6. DFT Calculations

**Figure 6:**
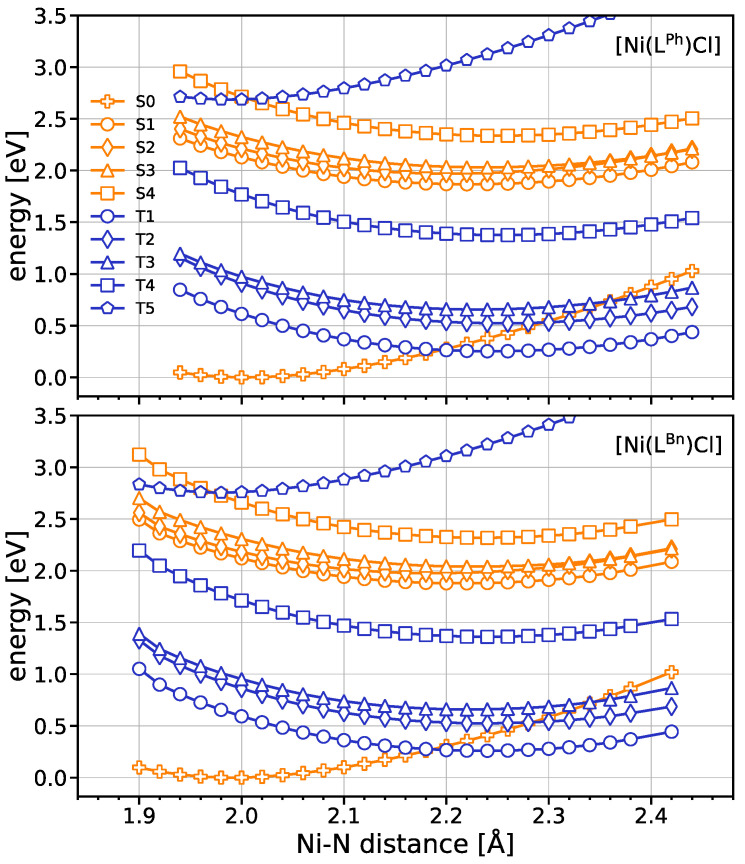
Ground and excited state energies for fixed Ni–N distances of the central [Ni(N^C^N)Cl] unit from time-dependent DFT (TD-DFT) calculations using the Tamm–Dancoff approximation (TDA) [59], in vacuum for both substituents, Ph (top) and Bn (bottom). Elongation of the Ni–N bonds promotes non-radiative transitions from *T*_1_–*T*_3_ to the *S*_0_ ground state due to conical intersections, whereas the *S*_1_ state should be emissive. In a rigid environment, where the Ni–N distance is kept at ca. 2.0 Å, luminescence could also occur from the *T*_5_ and *S*_4_ states.

As discussed above, the experimental molecular structures align very well with the DFT-calculated *S*_0_ ground state geometries. However, these differ significantly from the DFT-calculated *T*_1_ geometries (Figure S22), in which the Ni–N bonds stretch by about 0.3 Å. We initially hypothesized that the photoluminescence observed for the Ni(II) complexes in rigid matrices occurs from an excited triplet state, which is promoted by the restrictive environment suppressing the expansion of the Ni–N bonds. To investigate this scenario in more detail, we performed a scan of the first five singlet and triplet states using time-dependent DFT (TD-DFT) in the Tamm–Dancoff approximation (TDA) [60], varying the Ni–N bond lengths of the central [Ni(N^C^N)Cl] unit. Figure 6 shows potential curves obtained with the PBE0 exchange–correlation functional [61] and the SDD basis set [62]. It is worth mentioning that the Ni–N bond stretch correlates almost linearly with the out-of-plane bending of the Cl^–^ coligand (Figures S57 and S58) as it alleviates the steric hindrance responsible for the nonplanarity in the ground state geometry.

For both complexes, the population of the *T*_1_ to *T*_4_ or *S*_1_ to *S*_4_ states leads to a substantial Ni–N bond stretch (Figure 6). For the *T*_1_ to *T*_3_ states, conical intersections with the *S*_0_ ground state are clearly visible, enabling nonradiative decay and thus quenching the phosphorescence. In the case of the *T*_4_ state, the curves suggest that there may well be an energetically accessible crossing with the *S*_0_ state (or at least a very small gap) at a Ni–N distance beyond 2.45 Å. On the other hand, the *S*_1_ state is well separated from the *T*_4_ state and is therefore likely to be fluorescent. In a rigid environment that restricts the geometry to the Franck–Condon region (i.e., around 2.0 Å), the *T*_5_ and the *S*_4_ state could both contribute to the luminescence as well. In fact, a geometry optimization of [Ni(L^NHBn^)Cl] in the *S*_4_ state with the Ni–N bonds constrained to their ground state lengths, yielded emission wavelengths of 514 nm and 485 nm for the vertical and adiabatic transitions, respectively. The optimized *T*_5_ state of [Ni(L^NHBn^)Cl] gives an adiabatic (vertical) emission of 490 nm (556 nm). Thus, the calculated *S*_4_ and the *T*_5_ results match the experimental emission wavelengths quite well. Similarly, the optimized *T*_5_ state of [Ni(L^NHPh^)Cl] corresponds to an adiabatic (vertical) emission wavelength of 502 nm (568 nm), while the constraint-optimized *S*_4_ state with ground state Ni–N bond lengths yields 485 nm (514 nm).

The main electronic configurations contributing to the *T*_5_ state are HOMO→LUMO+1 (45%/44%) and HOMO-1→LUMO (33%/34%) excitations for [Ni(L^NHPh^)Cl] and [Ni(L^NHBn^)Cl], respectively (see Tables S15 and S16). The *S*_4_ state is mainly composed of the HOMO-13→LUMO+3 (48%) and HOMO-9→LUMO+3 (35%) excited configurations for [Ni(L^NHPh^)Cl], and the HOMO-14→LUMO+2 (48%) and HOMO-8→LUMO+2 (29%) for [Ni(L^NHBn^)Cl]. A visualization of the most relevant orbitals participating in the description of the *S*_1_, *S*_4_, and *T*_5_ states can be found in Figures S59 and S60. A triplet emission from the *T*_5_ state or *S*_4_ states is violating Kasha’s rule and is rarely reported [43,63,64,65]. However, for transition metal complexes, there are some recent examples for emission from higher triplet states [43,64,65].

To further support our argument, we performed TD-DFT calculations including spin–orbit coupling using the def2-TVZP basis set (for computational details, see Section 3.7; for detailed results, see Tables S17–S20) for both complexes at the optimized ground state geometry. For both species, [Ni(L^NHPh^)Cl] and [Ni(L^NHBn^)Cl], the lifetime of the *T*_5_ state is three orders of magnitude higher than that of the *S*_4_ state with 946 µs vs. 1 µs for [Ni(L^NHPh^)Cl] and 3.3 ms vs. 1 µs for [Ni(L^NHBn^)Cl]). These numbers confirm the general trend observed in the experiments, although the difference between the two lifetimes is somewhat less pronounced. It should be noted, however, that the frozen environment could not be rigorously taken into account in the calculations.

In a similar way, the photoluminescent Ni(II) complex [Ni(triazolato^Coumarin,COOCH3^)(dpb)] was studied and a lifetime of 197.2 µs for the excited triplet *T*_5_ state was calculated, which agrees very well with the experimental value of 147.8 µs. Thus, the low-T photoluminescence of this complex was assigned to an LC triplet state with perturbative MLCT character, while the emission observed at 298 K probably stems from the de-coordinated triazolo ligand as product of a dissociative process [43].

In contrast to this assignment, recent TD-DFT calculations on [Ni(dpb)Cl] on a PBE0/6-31G(d,p) level confirmed the ^1^MLCT character of the initially populated excited state, as concluded from nanosecond transient absorption spectroscopy (TAS), although the calculated wavelengths were slightly shorter than the experimental values [42]. Below this ^1^MLCT state, the calculations located three ^1^MC states which means that the initially excited ^1^MLCT state could undergo ISC to a ^3^MC state. This would explain the lacking photoluminescence of this complex at ambient T in solution and also in glassy frozen matrices at 77 K [38,42]. This study also showed that in the Pt(II) analog [Pt(dpb)Cl], the ^3^MC states lie higher in energy than the emissive ^3^LC states [42]. Very similar findings were reported for the pair of complexes [M(*C^C^C*)Cl] (M = Ni or Pt) with the *C^C^C* coordinating 1,3-bis(n-butyl-N-imidazolydene)phen-2-ide) ligand [42]. A similar TAS study on the isonitrile Ni(II) complexes [Ni(*C^C^C*)(CN-R)](PF_6_) showed that the enlargement of the R group from methyl, to 2,6-bis(3,5-xylyl)phenyl, and to 2,6-bis(3,5-isopropylphenyl)phenyl allowed to extend the lifetime of the excited ^3^MLCT state from 0.5 up to 48 ps, before decaying into a ^3^MC state [32]. The ^3^MC state are short-lived with lifetimes of 14 to 50 ps in CH_2_Cl_2_ and non-luminescent.

### 2.7. Femto- and Nanosecond Transient Absorption Spectroscopy (fs-TAS and ns-TAS)

To further analyze the excited state dynamics, femto- and nanosecond transient absorption spectroscopy was carried out either at room temperature or in a temperature range from 297 to 80 K.

Heat maps of fs-TAS following 365 nm photoexcitation of [Ni(L^NHPh^)Cl] and [Ni(L^NHBn^)Cl] in THF are best described by differential absorption changes across the visible that are positive up to 500 nm and negative from there on (Figures S61 and S62). The earlier represents an excited state absorption (ESA) with 450 nm maxima, while the latter is red-shifted relative to the ground state absorption and stems from stimulated emission (SE) rather than ground state bleaching (GSB) with minima around 600 nm. Global analysis with just one species afforded rather short lifetimes of 17.8 ps for [Ni(L^NHPh^)Cl] and 91.7 ps for [Ni(L^NHBn^)Cl] during which the ground state recovery is completed. In complementary room temperature ns-TAS, no appreciable transients were noted.

When cooling to 80 K, we noted in ns-TAS, additional ESAs at 400 and 580 nm and lifetimes of 722 ps for [Ni(L^NHPh^)Cl] and 1.94 ns for [Ni(L^NHBn^)Cl] (Figures S63–S66). These ESAs perfectly overlap with the ESAs seen for the ligand precursors ClL^NHBn^ and ClL^NHPh^ (Figures S67 and S68). As such, it confirms that in contrast to our low temperature emission studies, the recorded ESAs are ligand-centered and short-lived in nature.

Although being unlikely, considering the care that we have taken in the sample preparation, we cannot rule out any chemical decomposition. In that particular case, ligand-based ESAs would stem from the protonated and de-coordinated ligands HL^NHPh^ and HL^NHBn^ in solution and/or the matrix upon decomposition. Traces of the protonated ligands within the material of the Ni(II) complexes could possibly also be responsible for the short lifetime components in our photoluminescence measurements. However, neither in the low-T photoluminescence nor the ESA experiments we found any evidence for photodecomposition of the Ni(II) complexes. Thus, for the long-lived emissions, we can rule out that they stem from ligand species and it is very unlike that they stem from photoproducts. We are thus confident that they originate from the phosphorescence of the intact Ni(II) complexes, although we cannot confirm them using TAS.

A recent fs-TAS study on the [Ni(dpb)Cl] complex showed ESAs with a lifetime of 0.9 ps in CH_2_Cl_2_ and 0.7 ps in MeCN, resembling the absorption spectra of the one-electron reduced complex [42]. Based on this resemblance, they were assigned to a ^1^MLCT excited state which decays to a ^3^MC excited state with a lifetime of 14 ps [42]. The combined ESA and ground state bleach (GSB) occurred within 100 ps. As outlined above, TD-DFT calculated confirmed the ^1^MLCT character of the initially populated excited state and suggested a rapid ISC to the excited ^3^MC state within fractions of a ps [42].

Remarkably, we were not able to detect in our study the same ESA and GSB features reported for [Ni(dpb)Cl], although the lifetimes of the ESA and GSB features found for this complex lie in the same ps range as our ESAs and SEs. We might explain this difference with the markedly different geometries of the complexes [Ni(L^NHPh^)Cl] and [Ni(L^NHBn^)Cl] with their pronounced deviation from a planar coordination expressed by the τ_4_ values of 0.336 and 0.318, in contrast to the almost completely planar [Ni(dpb)Cl] [38,45]. It is interesting to note that, in our hands, [Ni(dpb)Cl] turned out to be far more stable than [Ni(L^NHPh^)Cl] and [Ni(L^NHBn^)Cl].

## 3. Experimental Section

### 3.1. Materials and Syntheses

Experimental details on the materials and syntheses are provided in the Supplementary Materials.

### 3.2. Instrumentation

^1^H and ^13^C spectra were recorded on a *Bruker* Avance II 300 MHz (^1^H: 300 MHz, ^13^C: 75 MHz) equipped with a double resonance (BBFO) 5 mm observe probe head with z-gradient coil, and Bruker Avance II 600 MHz spectrometer (^1^H: 600 MHz, ^13^C: 151 MHz) with a triple resonance (TBI) 5 mm inverse probe head with z-gradient coil using a triple resonance (Bruker, Rheinhausen, Germany). The unambiguous assignment of the ^1^H and ^13^C resonances was obtained from ^1^H NOESY, ^1^H COSY, ^1^H ^13^C HSQC, and ^1^H ^13^C HMBC experiments. All 2D NMR experiments were performed using standard pulse sequences from the Bruker pulse program library. Chemical shifts were measured relative to TMS (^1^H, ^13^C). UV-vis absorption spectra were recorded on Varian Cary 05E spectrophotometer (Varian Medical Systems, Darmstadt, Germany). Elemental analyses were obtained using a HEKAtech CHNS EuroEA 3000 analyzer (HEKAtech, Wegberg, Germany). HR-ESI-MS(+) spectra were measured at the Thermo Scientific LTQ OrbitrapXL mass spectrometer via electron spray ionization and a FTMS Analyzer (Thermo Fisher Scientific GmbH, Dreieich, Germany). EI-MS spectra in the positive mode were measured using a Finnigan MAT 95 mass spectrometer (Thermo Fisher Scientific GmbH, Dreieich, Germany). Simulations were performed using ISOPRO 3.0 (free download: https://isopro.software.informer.com/3.0/ (accessed on 4 March 2025)). Electrochemical measurements were carried out in 0.1 M *n*-Bu_4_NPF_6_ solution in THF (tetrahydrofuran) using a three-electrode configuration (glassy carbon electrode, Pt counter electrode, Ag/AgCl reference) and a Metrohm Autolab PGSTAT30 or µStat400 potentiostat (Metrohm, Filderstadt, Germany). The potentials were referenced against the ferrocene/ferrocenium redox couple as internal standard.

### 3.3. Crystal Structure Determination

Single crystals of [Ni(L^NHPh^)Cl] and [Ni(L^NHBn^)Cl] were grown by layering saturated solutions in argon-purged, de-aerated CH_2_Cl_2_ with a mixture of *n*-pentane and *n*-hexane (8:2) under argon flow. Measurements were carried out at 100(2) K, employing a Bruker D8 Venture including a Bruker Photon 100 CMOS detector using Mo Kα radiation (λ = 0.71073 Å) (Bruker, Rheinhausen, Germany). The crystal data were collected using APEX3 v2015.5–2 [66]. The structures were solved using dual space methods using SHELXT [67] and the refinement was carried out with SHELXL 2017 employing full-matrix least-squares methods on *F*_0_^2^ ≥ 2σ(*F*_0_^2^) [68]. The non-hydrogen atoms were refined with anisotropic displacement parameters without any constraints. The hydrogen atoms were included by using appropriate riding models. Data of the structure solutions and refinements can be obtained for [Ni(L^NHPh^)Cl] (CCDC 2246584), and [Ni(L^NHBn^)Cl] (CCDC 2252984), free of charge at https://summary.ccdc.cam.ac.uk/structure-summary-form (accessed on 5 March 2025) or from the Cambridge Crystallographic Data Centre, 12 Union Road, Cambridge, CB2 1EZ UK (e-mail: deposit@ccdc.cam.ac.uk).

### 3.4. Photophysical Measurements

Steady-state excitation and emission spectra were recorded on a FluoTime 300 spectrometer from PicoQuant (PicoQuant, Berlin, Germany) equipped with a 300 W ozone-free Xe lamp (250–1100 nm), a 10 W Xe flash-lamp (250 to 1100 nm, pulse width ca. 1 µs) with repetition rates of 0.1 to 300 Hz, a double-grating excitation monocromator (Czerny-Turner type, grating with 1200 lines/mm, blazed wavelength: 300 nm), diode lasers (pulse width < 20 ps) operated by a computer-controlled laser driver PDL-828 “Sepia II” (repetition rate up to 80 MHz, burst mode for slow and weak decays), two emission monochromators (Czerny–Turner, selectable between double-grating blazed at 500 nm with 2.7 nm/mm dispersion and 1200 lines/mm, or single-grating blazed at 1250 nm with 5.4 nm/mm dispersion and 600 lines/mm) with an adjustable slit width between 25 µm and 7 mm, Glan–Thompson polarizers for excitation (after the Xe-lamps), and emission (after the sample). Different sample holders (Peltier-cooled mounting unit ranging from –15 to 110 °C or an adjustable front-face sample holder), along with two detectors (namely a PMA Hybrid-07 from PicoQuant with transit time spread FWHM < 50 ps, 200 to 850 nm, or a H10330C-45-C3 NIR detector with transit time spread FWHM 0.4 ns, 950 to 1400 nm from Hamamatsu), were used. Steady-state spectra and photoluminescence lifetimes were recorded in TCSPC mode using a PicoHarp 300 (minimum base resolution 4 ps) or in MCS mode by a TimeHarp 260 (where up to several ms can be traced). Emission and excitation spectra were corrected for source intensity (lamp and grating) by standard correction curves. For samples with lifetimes in the ns order, an instrument response function calibration (IRF) was performed using a diluted Ludox^®^ dispersion. Lifetime analysis was performed using the commercial EasyTau 2 software (PicoQuant). The quality of the fit was assessed by minimizing the reduced chi squared function (χ^2^) and visual inspection of the weighted residuals and their autocorrelation. For the low temperature measurements (up to 6 K), a Cryostat model DE-204S (ARS) with two DT-670B-SD thermocouples attached, coupled to a Helium ARS-4HW Compressor (ARS) was used. Temperature was controlled using a Cryogenic Temperature Controller (Lake Shore, Model 335, Westerville, OH, USA).

Luminescence micrographs were acquired using an optical microscope (IX 73 from Olympus, Olympus Europa SE&Co.KG, Hamburg, Germany), equipped with a X CiteQ Lamp module (Excelitas Technologies, Pittsburgh, PA, USA) as excitation source and a UI 5580SE (IDS) digital camera. Different band pass (BP) and low pass (LP) cubes were used accordingly.

Luminescence quantum yields were measured with a Hamamatsu Photonics absolute PL quantum yield measurement system (C9920-02) equipped with a L9799-01 CW Xenon light source (150 W), monochromator, C7473 photonic multi-channel analyzer, integrating sphere and employing U6039-05 PLQY measurement software (Hamamatsu Photonics Deutschland GmbH, Herrsching am Ammersee, Germany). All cuvettes used were round quartz cuvettes and the solvents were of spectrometric grade (Uvasol^®^, Merck, Darmstadt, Germany).

Femtosecond transient absorption (fsTA) experiments were conducted employing an amplified Ti:sapphire CPA-2101 laser system from Clark:MXR Inc. (Dexter, MI, USA) as excitation source. The generated lasers pulses are characterized by an initial wavelength of 775 nm, a pulse width of 150 fs, and a frequency of 1050 Hz. The detection of the data was realized using a HELIOS “transient absorption pump-probe system” (TAPPS) detection unit from Ultrafast Systems (Sarasota, FL, USA). The 365 nm excitation wavelengths, with energies of roughly 500 nJ, were generated by a noncolinear optical parametric amplifier (NOPA, Clark:MXR Inc.), whereas the white light (probe pulse) is generated by a sapphire crystal. An optical delay line allowed for time delays up to 5.5 ns. The experiments were carried out using fused quartz glass cuvettes with a width of 2 mm. All of the samples were deoxygenated for approximately ten minutes using argon gas and measured at an optical density of roughly 0.2 at the respective excitation wavelength. For data recording, the HELIOS system from Newport photonics (Irvine, CA, USA)/Ultrafast Systems was utilized.

Nanosecond transient absorption (nsTA) experiments were recorded with the EOS TAPPS detection unit from Ultrafast Systems using two independent pulsed laser sources. While the pump pulse is also generated by an amplified Ti:sapphire CPA-2101 laser system from Clark:MXR Inc., the probe pulse is generated by a pulsed supercontinuum laser (output 350–2200 nm, repetition rate of 2100 Hz, and 1 ns pulse width). The electronic setup allows for time delays up to 400 μs between the pump and probe pulse. The excitation wavelength of 365 nm were generated by a noncolinear optical para-metric amplifier (NOPA, Clark:MXR Inc.).

For the nsTA measurements, global analysis and fitting of the transient data were carried out utilizing the open-source software GloTarAn (Version 1.5.1), which is a free, Java-based graphical user interface to the R-package TIMP (Version 3.3.2) [69,70,71]. Global analysis was performed on the TA data sets using a sequential model. The instrument response function (IRF) and dispersion (chirp of the white light pulse) were modeled and considered during the fitting procedure. The nsTA data sets were corrected for scattered light as well. Cryostat supported measurements between 297 and 80 K were conducted using an Optistat DN2 cryostat from Oxford Instruments (Abingdon, UK) in 2 methyltetrahydrofuran (MeTHF, anhydrous, ≥99%, Sigma-Aldrich, Germany).

### 3.5. Quantum Chemical Calculations Using Density Functional Theory (DFT)

All calculations were performed using the Gaussian 16 suite of programs [72] with the hybrid functional TPSSh and including τ-dependent gradient corrected correlation [73]. The LanL2DZ [74] basis set was used with effective core potentials ecp(10/18) for Ni [75,76,77], while the 6-31G(d)[78] basis set was used for all non-metallic elements. The solvents THF and CH_2_Cl_2_ were included by using the C-PCM solvation model [79,80] implemented in Gaussian. Geometry optimization of all complexes [Ni(^Y^N^C^N^Y^)X] (X = Cl, F, H; Y = NHPh, NHBn, MeO) was followed by frequency calculations and yielded no imaginary modes, thus confirming the energetically minimal nature of the stationary points. To obtain the UV-vis absorption spectrum of the complexes, TD-DFT calculations of the 45 lowest excited singlet states were performed at the optimized *S*_0_ geometry. Molecular orbitals and electronic properties were extracted from the single point calculations.

### 3.6. Structural Distortion Calculations Using Time-Dependent Density Functional Theory (TD-DFT) with the Tamm–Dancoff Approximation (TDA)

All calculations were performed using the Gaussian 16 suite of programs [72] with the PBE0 exchange–correlation functional [61] including the D3 version of Grimme’s dispersion with Becke–Johnson damping [81] for the van der Waals interaction. The SDD basis set, which applies an effective core potential for the metal atoms [62], was used for all elements, including nickel. Here, no PCM solvation models were used to simulate a solvent. Molecular orbitals were visualized using VMD [82] with an isovalue of 0.02. Geometrical optimizations in the ground state *S*_0_ were carried out with Kohn–Sham DFT for scans, where the two-central nickel-nitrogen bonds were fixed at increasing values. For each resulting geometry a TD-DFT calculation with TDA [60] was conducted.

### 3.7. Radiative Lifetime Calculations Using TD-DFT with Spin–Orbit Coupling

TD-DFT calculations including spin–orbit coupling (SOC) were performed with the ORCA program package 5.0.4 [83,84] using the PBE0 functional and the def2-TZVP basis set [85]. The optimized geometry was taken from the calculations without SOC (see Section 3.6). SOC-TD-DFT [86] calculations with the TDA of the first 50 excited singlet and triplet states were performed in combination with the scalar relativistic zeroth-order regular approximation (ZORA) [87] and the ZORA-adapted ZORA-def2-TZVP basis. To accelerate the calculation, the RI-SOMF(1X) method [88] was employed for the Coulomb part.

## 4. Conclusions

The two N^C^N-cyclometalated complexes [Ni(L^NHPh^)Cl] and [Ni(L^NHBn^)Cl] containing 2,6-di(2-pyridyl)phen-1-ide ligands with pendent amine groups (L^NHPh^ with R = NH(C₆H₅); L^NHBn^ with R = NH(CH_2_C₆H₅)) show a marked deviation of the Cl^−^ coligand (ancillary ligand) from the ideally planar coordination in their molecular structures, with τ_4_ values of 0.35 and 0.33, respectively. We found that the steric strain of the ligand, rather than the observed N–H^…^Cl^…^H–N hydrogen bonding interactions of the NH function with the Cl^−^ coligand, is responsible for this observation. Importantly, the experimental molecular geometries from X-ray diffractometry agree very well with the DFT-calculated *S*_0_ conformation. The DFT-calculated excited *T*_1_ state geometry involves significantly elongated Ni–N bonds.

The complexes show long-wavelength absorption bands at around 425 nm in solution, and the experimental spectra are well reproduced by TD-DFT calculations. A detailed theoretical analysis of the potential energy landscapes of the first 10 excited states has revealed that the lowest triplet states decay non-radiatively due to conical intersections with the ground state. Contributions to the photoluminescence could originate from the *S*_1_, *S*_4_, or *T*_5_ states. The calculated emission wavelengths from the *T*_5_ state agree particularly well with the experimental results. If the elongation of the Ni–N bonds is suppressed by a rigid environment, the calculated *S*_4_ emission wavelengths also lie close to the experimental results. Consequently, the complexes display structured emission spectra in frozen 2MeTHF glassy matrices at 77 K. Consistently, lifetimes in the millisecond and nanosecond range were observed. Moreover, the photophysical properties of the complexes within a poly(methyl methacrylate) (PMMA) matrix were evaluated across an extensive temperature range, where the complexes exhibited well-defined emission spectra and long photoluminescence lifetimes with measurable quantum yields, even at room temperature. Femto- and nanosecond transient absorption spectroscopy at 297 or 80 K gave only short-lived excited state species and our present assumption is that they are very probably ligand-based. When considering the possibility of partial hydrolysis of the very sensitive Ni(II) complexes and the formation of traces of ligand species, the short-lived photoluminescence components might be due to ligand species in the samples. But the long-lived photoluminescence in the ms range stems very probably from the Ni(II) complexes. Furthermore, we assume that the ligand scaffold rigidifying the electronic *S*_0_ ground state in combination with the low-T matrices rigidifying the excited *S*_4_ or *T*_5_ states promote the photoluminescence, in keeping with our initial research idea of the conformational locking of ground and excited state geometries.

The previously reported very similar complex [Ni(dpb)Cl] (Hdpb = 1,3-bis(2-pyridylbenzene) allowed us to study an excited ^1^MLCT state decaying into a dark ^3^MC state within fractions of ps, which explains lacking photoluminescence of the complex at both ambient T and 77 K. But in contrast to this complex, which shows a perfectly planar geometry in the ground state but a strong D_4h_ to the D_2h_ distortion in the MC states, our complexes [Ni(L^NHPh^)Cl] and [Ni(L^NHBn^)Cl] show only very small distortions between ground and excited states. Although we cannot currently supply unequivocal proof that the observed ms emission stems from the Ni(II) complexes, as the herein presented complexes are quite labile, the introduction of the rigid environment at the ortho-to-N-positions of the pyridyl groups might pave the way to photoluminescent Ni(II) complexes with long lifetimes.

## Figures and Tables

**Figure 5:**
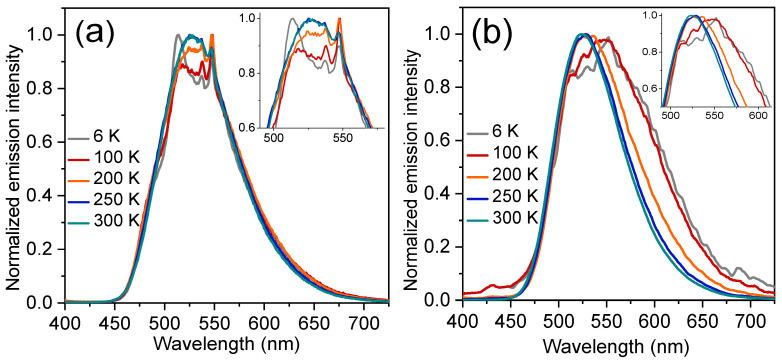
Photoluminescence spectra of the complexes [Ni(L^NHPh^)Cl] (**a**) and [Ni(L^NHBn^)Cl] (**b**) (*λ*_ex_ = 350 nm) at different temperatures within poly(methyl methacrylat) (PMMA) films (2.5%).

**Table 1:** Selected photophysical data for the [Ni(N^C^N)Cl] complexes ^a^.

		[Ni(L^NHPh^)Cl]	[Ni(L^NHBn^)Cl]
	*λ*_em, max_ (nm)	510	504
2MeTHF (77 K)	*Φ*_PL_ ± 0.05	0.30	0.40
	*τ*_av_amp_ (ns) *τ*_av_amp_ (ms)	3.34 ± 0.01 648 ± 3	3.63 ± 0.02 180 ± 10
PMMA (6 K)	*τ*_av_amp_ (ns) *τ*_av_amp_ (ms)	1.91 ± 0.04 476 ± 6	2.73 ± 0.02 93 ± 3
PMMA (100 K)	*τ*_av_amp_ (ns) *τ*_av_amp_ (ms)	1.80 ± 0.05 464 ± 6	2.69 ± 0.03 74 ± 3
PMMA (200 K)	*τ*_av_amp_ (ns) *τ*_av_amp_ (ms)	1.69 ± 0.04 161 ± 5	2.46 ± 0.05 16 ± 1
PMMA (250 K)	*τ*_av_amp_ (ns) *τ*_av_amp_ (ms)	1.51 ± 0.03 163 ± 4	2.24 ± 0.04 10.4 ± 0.2
PMMA (300 K)	*τ*_av_amp_ (ns) *τ*_av_amp_ (ms)	1.41 ± 0.04 143 ± 5	2.18 ± 0.02 1.05 ± 0.01
	*Φ*_PL_ ± 0.05	0.03 ± 0.02	0.04 ± 0.02

^a^ In a frozen 2-methyltetrahydrofuran (2MeTHF) glassy matrix at 77 K or immersed in a poly(methyl methacrylate) (PMMA) matrix at different temperatures (where short and long lifetimes were detected). *Φ*_PL_: photoluminescence quantum yields. (*τ*_av_amp_: amplitude-weighted average lifetimes from multi-exponential decays). Raw time-resolved photoluminescence decay with all the components and fitting parameters are shown in Figures S27–S48.

## Data Availability

Deposition numbers CCDC 2246584 ([Ni(L^NHPh^)Cl]), 2252984 ([Ni(L^NHBn^)Cl]), and 2194775 (HL^NHPh^) contain the supplementary crystallographic data for this paper. These data are provided free of charge by the Cambridge Crystallographic Data Centre Access Structures service www.ccdc.cam.ac.uk/structures (accessed on 5 March 2025). All other data are available from the authors on request.

## Supplementary Materials

The following supporting information can be downloaded at https://www.mdpi.com/article/10.3390/molecules30091901/s1. Materials and Syntheses, Supplementary Figures S1–S9: ^1^H and ^13^C NMR spectra, High-resolution ESI-MS(+) spectra. Figure S10: UV-vis absorption spectra of the [Ni(N^C^N)Cl] complexes over time in THF solution at 298 K. Figures S11–S14: Crystal structure and molecular structures. Figures S15 and S16: DFT-calculated molecular structures. Figure S17: Cyclic voltammograms. Figure S18: DFT-calculated energies of the highest occupied (HOMO) and lowest unoccupied (LUMO) molecular orbitals. Figures S19 and S20: Experimental UV-vis absorption spectra with TD-DFT-calculated transitions. Figure S21: DFT-calculated energies of the lowest singly occupied (LSOMO) and highest singly occupied (HSOMO) molecular orbitals. Figure S22: DFT-calculated geometries *S*_0_ and *T*_1_ states. Figure S23: DFT-calculated energies as a function of the Ni–Cl bond length and the C1–Ni–Cl angle. Figure S24: TG and DTA. Figures S25–S48: Time-resolved photoluminescence decays and fitting parameters for the complexes. Figure S49: Emission spectra of the complexes, their ligand precursors ClL^NHPh^ and ClL^NHBn^, and the protonated ligands HL^NHPh^ and H^LNHBn^. Figure S50–S56: Time-resolved fluorescence decay, the instrument response functions and fitting parameters for the ligand and the protonated ligands. Figures S57 and S58: Dihedral angles Cl–Ni–N–C(1) and Cl–Ni–N–C(2), and bond angle Cl–Ni–C for the constraint optimized structures along the Ni–N coordinate. Figures S59 and S60: Main contributing orbitals to the *S*_1_→*S*_0_, *S*_4_→*S*_0_, and *T*→*S*_0_ from TDA calculations. Figures S61 and S62: 3D heat map of the Ni(II) complexes at 298 K obtained by cryostat supported fs-TAS measurements in THF and evolution associated spectra. Figure S63–S66: 3D heat maps of from ns-TAS measurements and evolution associated spectra of the complexes. Figures S67 and S68: 3D heat maps from ns-TAS measurements and evolution associated spectra of the ligand precursors with a lifetime of 8.3 ns. Supplementary Tables: Table S1: Crystal data and structure refinement for the complexes. Table S2: Experimental and DFT-calculated geometries for the complexes. Table S3: Selected experimental structural data for the complexes. Table S4: DFT-calculated geometries of the [Ni(N^C^N)Cl] (N^C^N = LNHPh or LNHBn). Table S5: Selected DFT-calculated geometries for the complexes in the *S*_0_ ground state. Table S6: Redox potentials of the complexes. Tables S7–S10: DFT-calculated compositions (%) of frontier MOs in the *S*_0_ and *T*_1_ states for the complexes. Table S11: Selected UV-vis absorption maxima of the complexes. Tables S12 and S13: Selected TD-DFT-calculated vertical *S*_0_→*S_n_* transitions for the complexes. Table S14: Excited state lifetimes of the ligand precursors ClLNHPh and ClLNHBn and the protonated ligands HLNHPh, and HLNHBn. Tables S15 and S16: Characterization of selected PBE0/SDD/TDA-calculated vertical excited states for the optimized ground state geometry of the complexes. Tables S17 and S18: Characterization of selected PBE0/DEF2-TZVP/TDA-calculated excited states. Tables S19 and S20: Radiative lifetime calculations from SOC-TDA. Tables S21 and S22: Calculated coordinates of the optimized singlet ground state structures of the complexes. Reference [89].
