# Supplementary material for: Conformational Locking of the Geometry in Photoluminescent Cyclometalated N^C^N Ni(II) Complexes
Source: Molecules. 2025 Apr 24;30(9):1901. doi: 10.3390/molecules30091901 (PMC12073815; doi:10.3390/molecules30091901)
Supplement: Supplementary file 1 [file molecules-30-01901-s001.zip › molecules-3580135-supplementary.pdf]

Supplementary Materials

Conformational Locking of the Geometry in Photoluminescent Cyclometalated N^C^N Ni(II) Complexes

Maryam Niazi [1], Iván Maisuls [2,3], Lukas A. Mai [4], Sascha A. Schäfer [1], Alex Oster [5], Lukas S. Diaz [4], Dirk M. Guldi [4], Nikos L. Doltsinis [5,*], Cristian A. Strassert [2,3,*] and Axel Klein [1,*]

1 Department of Chemistry and Biochemistry, Institute for Inorganic and Materials Chemistry, Faculty for Mathematics and Natural Sciences, University of Cologne, Greinstraße 6, D-50939 Köln, Germany;
mniazi1@smail.uni-koeln.de (M.N.); sascha.schaefer@uni-koeln.de (S.A.S.)

2 Institut für Anorganische und Analytische Chemie, Universität Münster, Corrensstraße 28/30, D-48149 Münster, Germany; maisuls@uni-muenster.de

3 CiMIC, CeNTech, Heisenbergstraße 11, D-48149 Münster, Germany

4 Department of Chemistry and Pharmacy & Interdisciplinary Center for Molecular Materials (ICMM),
Friedrich-Alexander-University Erlangen-Nuremberg, Egerlandstraße 3, D-91058 Erlangen, Germany;
lukas.mai@fau.de (L.A.M.); lukas.santiago@fau.de (L.S.D.); dirk.guldi@fau.de (D.M.G.)

5 Institut für Festkörpertheorie and Center for Multiscale Theory and Computation, Universität Münster, Wilhelm-Klemm-Straße 10, D-48149 Münster, Germany; oster.alex@uni-muenster.de

* Correspondence: nikos.doltsinis@uni-muenster.de (N.L.D.); cstra_01@uni-muenster.de (C.A.S.); axel.klein@uni-koeln.de (A.K.)

Contents

Materials and Syntheses

Supplementary Figures

Figure S1. 600 MHz ^{1}H NMR spectrum of ClL^{NHPh} in DMSO-d_6 at RT.
Figure S2. 151 MHz ^{13}C NMR spectrum of ClL^{NHPh} in DMSO-d_6 at RT.
Figure S3. 600 MHz ^{1}H NMR spectrum of ClL^{NHBn} in DMSO-d_6 at RT.
Figure S4. 151 MHz ^{13}C NMR spectrum of ClL^{NHBn} in DMSO-d_6 at RT.
Figure S5. 300 MHz ^{1}H NMR spectrum of [Ni(L^{NHPh})Cl] in DMSO-d_6 at RT.
Figure S6. 600 MHz ^{1}H NMR spectrum of [Ni(L^{NHBn})Cl] in DMSO-d_6 at RT.
Figure S7. High-resolution ESI-MS(+) spectrum of [Ni(L^{NHPh})Cl] and calculated pattern for [M–Cl]$^{+}$.
Figure S8. High-resolution ESI-MS(+) spectrum of [Ni(L^{NHBn})Cl] and calculated pattern [M–Cl]$^{+}$.
Figure S9. ^{1}H NMR spectra of [Ni(L^{NHBn})Cl] and ^{1}H NMR spectra of HL^{NHBn} and ClL^{NHBn}, in DMSO-d_6.
Figure S10. UV-vis absorption spectra of the [Ni(N^C^N)Cl] complexes over time in THF solution at 298 K.
Figure S11. Crystal structure and molecular structure of [Ni(L^{NHPh})Cl] from single crystal X-ray diffractometry with photoluminescence micrographs of the complex in the crystal state at 298 K.
Figure S12. Crystal structure of [Ni(L^{NHPh})Cl] from single crystal X-ray diffractometry.
Figure S13. Intermolecular contacts and Ni···Ni distances (Å) in [Ni(L^{NHPh})Cl].
Figure S14. Crystal structure and molecular structure of [Ni(L^{NHBn})Cl] from single crystal X-ray diffractometry with photoluminescence micrographs of the complex in the crystal state at 298 K.
Figure S15. DFT-calculated molecular structures of [Ni(L^{NHPh})Cl] and [Ni(L^{NHBn})Cl], in the S_0 ground state and deviation τ_4 from square planar coordination.
Figure S16. DFT-calculated molecular structures of the complexes [Ni(L^{NHPh})F], [Ni(L^{NHPh})H], and [Ni(L^{OMe})Cl] in the S_0 ground state and deviation τ_4 from square planar coordination.
Figure S17. Cyclic voltammograms of [Ni(N^C^N)Cl] in n-Bu$_4$NPF$_6$/THF.
Figure S18. DFT-calculated energies of the highest occupied (HOMO) and lowest unoccupied (LUMO) molecular orbitals for [Ni(L^{NHPh})Cl] and [Ni(L^{NHBn})Cl].

Figure S19. Experimental UV-vis absorption spectra of ClL^{NHPh}, and $[Ni(L^{NHPh})Cl]$ in THF at 298 K with TD-DFT-calculated transitions (sticks in red).
Figure S20. Experimental UV-vis absorption spectra of ClL^{NHBn}, and $[Ni(L^{NHBn})Cl]$ in THF at 298 K with TD-DFT-calculated transitions (sticks in red).
Figure S21. DFT-calculated energies of the lowest singly occupied (LSOMO) and highest singly occupied (HSOMO) molecular orbitals for [Ni(N^C^N)Cl], N^C^N = L^{NHPh} and L^{NHBn} .
Figure S22. DFT-calculated geometries of [Ni(N^C^N)Cl] (N^C^N = L^{NHPh} and L^{NHBn}) in the S_0 and T_1 states.
Figure S23. DFT-calculated energies as a function of the Ni–Cl bond length and the C1–Ni–Cl angle in [Ni(N^C^N)Cl], N^C^N = L^{NHPh} and L^{NHBn}.
Figure S24. TG and DTA of [Ni(N^C^N)Cl] (N^C^N = L^{NHPh} and L^{NHBn}) under argon atmosphere.
Figure S25. Time-resolved photoluminescence decay of $[Ni(L^{NHPh})Cl]$ in a frozen glassy matrix of 2MeTHF at 77 K, including the residuals (λ_{ex} = 376 nm).
Figure S26. Time-resolved photoluminescence decay of $[Ni(L^{NHBn})Cl]$ in a frozen glassy matrix of 2MeTHF at 77 K, including the residuals (λ_{ex} = 376 nm).
Figure S27. Time-resolved photoluminescence decay of $[Ni(L^{NHPh})Cl]$ within a PMMA film at 6 K, including the residuals (λ_{ex} = 376 nm).
Figure S28. Raw (experimental) time-resolved phosphorescence decay of $[Ni(L^{NHPh})Cl]$ within a PMMA film at 100 K, including the residuals (λ_{ex} = 376 nm, λ_{em} = 525 nm).
Figure S29. Raw (experimental) time-resolved phosphorescence decay of $[Ni(L^{NHPh})Cl]$ within a PMMA film at 200 K, including the residuals (λ_{ex} = 376 nm, λ_{em} = 525 nm).
Figure S30. Raw (experimental) time-resolved phosphorescence decay of $[Ni(L^{NHPh})Cl]$ within a PMMA film at 250 K, including the residuals (λ_{ex} = 376 nm, λ_{em} = 525 nm).
Figure S31. Raw (experimental) time-resolved phosphorescence decay of $[Ni(L^{NHPh})Cl]$ within a PMMA film at 300 K, including the Residuals (λ_{ex} = 376 nm, λ_{em} = 525 nm).
Figure S32. Raw (experimental) time-resolved phosphorescence decay of $[Ni(L^{NHBn})Cl]$ within a PMMA film at 6 K, Including the residuals (λ_{ex} = 376 nm, λ_{em} = 525 nm).
Figure S33. Raw (experimental) time-resolved phosphorescence decay of $[Ni(L^{NHBn})Cl]$ within a PMMA film at 100 K, including the residuals (λ_{ex} = 376 nm, λ_{em} = 525 nm). Fitting parameters including pre-exponential factors and confidence limits.
Figure S34. Raw (experimental) time-resolved phosphorescence decay of $[Ni(L^{NHBn})Cl]$ within a PMMA film at 200 K, including the residuals (λ_{ex} = 376 nm, λ_{em} = 525 nm).
Figure S35. Raw (experimental) time-resolved phosphorescence decay of $[Ni(L^{NHBn})Cl]$ within a PMMA film at 250 K, including the residuals (λ_{ex} = 376 nm, λ_{em} = 525 nm).
Figure S36. Raw (experimental) time-resolved phosphorescence decay of $[Ni(L^{NHBn})Cl]$ within a PMMA film at 300 K, including the residuals (λ_{ex} = 376 nm, λ_{em} = 525 nm).
Figure S37. Raw (experimental) fluorescence decay of $[Ni(L^{NHPh})Cl]$ within a PMMA film at 6 K (blue) and the instrumental response function (IRF, red) including the residuals (λ_{ex} = 376 nm, λ_{em} = 525 nm).
Figure S38. Raw (experimental) fluorescence decay of $[Ni(L^{NHPh})Cl]$ within a PMMA film at 100 K (blue) and the instrumental response function (IRF, red) including the residuals (λ_{ex} = 376 nm, λ_{em} = 525 nm).
Figure S39. Raw (experimental) fluorescence decay of $[Ni(L^{NHPh})Cl]$ within a PMMA film at 200 K (blue) and the instrumental response function (IRF, red) including the residuals (λ_{ex} = 376 nm, λ_{em} = 525 nm).
Figure S40. Raw (experimental) fluorescence decay of $[Ni(L^{NHPh})Cl]$ within a PMMA film at 250 K (blue) and the instrumental response function (IRF, red) including the residuals (λ_{ex} = 376 nm, λ_{em} = 525 nm).
Figure S41. Raw (experimental) fluorescence decay of $[Ni(L^{NHPh})Cl]$ within a PMMA film at 300 K (blue) and the instrumental response function (IRF, red) including the residuals (λ_{ex} = 376 nm, λ_{em} = 525 nm).
Figure S42. Raw (experimental) fluorescence decay of $[Ni(L^{NHBn})Cl]$ within a PMMA film at 6 K (blue) and the instrumental response function (IRF, red) including the residuals (λ_{ex} = 376 nm, λ_{em} = 525 nm).
Figure S43. Raw (experimental) fluorescence decay of $[Ni(L^{NHBn})Cl]$ within a PMMA film at 100 K (blue) and instrumental response function (IRF, red) including the residuals (λ_{ex} = 376 nm, λ_{em} = 525 nm).
Figure S44. Raw (experimental) fluorescence decay of $[Ni(L^{NHBn})Cl]$ within a PMMA film at 200 K (blue) and the instrumental response function (IRF, red) including the residuals (λ_{ex} = 376 nm, λ_{em} = 525 nm).

Figure S45. Raw (experimental) fluorescence decay of [Ni(L^{NHBn})Cl] within a PMMA film at 250 K (blue) and the instrumental response function (IRF, red) including the residuals (λ_{ex} = 376 nm, λ_{em} = 525 nm).
Figure S46. Raw (experimental) fluorescence decay of [Ni(L^{NHBn})Cl] within a PMMA film at 300 K (blue) and the instrumental response function (IRF, red) including the residuals (λ_{ex} = 376 nm, λ_{em} = 525 nm).
Figure S47. Raw (experimental) fluorescence decay of [Ni(L^{NHPh})Cl] within a glassy matrix (2MeTHF, c = 10^{-5} M) at 77 K (blue) and the instrumental response function (IRF, red) including the residuals (λ_{ex} = 376 nm, λ_{em} = 525 nm).
Figure S48. Raw (experimental) fluorescence decay of [Ni(L^{NHBn})Cl] within a glassy matrix (2MeTHF, c = 10^{-5} M) at 77 K (blue) and the Instrumental Response Function (IRF, red) including the residuals (λ_{ex} = 376 nm, λ_{em} = 525 nm).
Figure S49. Emission spectra of [Ni(N^C^N)Cl] (N^C^N = L^{NHPh} and L^{NHBn}) complexes (black), their ligand precursors ClL^{NHPh} and ClL^{NHBn}, and the protonated ligands HL^{NHPh} and HL^{NHBn} (all in red) in frozen glassy matrix (2MeTHF, *c* = 10^{-5} M), λ_{ex} = 350 nm.
Figure S50. Time-resolved fluorescence decay (blue) of **ClL^{NHPh}** and the instrument response function (IRF, red) in a frozen glassy matrix of 2MeTHF at 77 K, including the residuals (λ_{ex} = 376 nm, λ_{obs} = 475 nm).
Figure S51. Time-resolved phosphorescence decay (blue) of **ClL^{NHPh}** in a frozen glassy matrix of 2MeTHF at 77 K, including the residuals (λ_{ex} = 376 nm, λ_{obs} = 475 nm).
Figure S53. Time-resolved fluorescence decay (blue) of **ClL^{NHBn}** and the instrument response function (IRF, red) in a frozen glassy matrix of 2MeTHF at 77 K, including the residuals (λ_{ex} = 376 nm, λ_{obs} = 475 nm).
Figure S54. Time-resolved phosphorescence decay (blue) of **ClL^{NHBn}** in a frozen glassy matrix of 2MeTHF at 77 K, including the residuals (λ_{ex} = 376 nm, λ_{obs} = 475 nm). **Right**: Fitting parameters including pre-exponential factors and confidence limits.
Figure S55. Time-resolved fluorescence decay (blue) of **HL^{NHPh}** and the instrument response function (IRF, red) in a frozen glassy matrix of 2MeTHF at 77 K, including the residuals (λ_{ex} = 376 nm, λ_{obs} = 475 nm).
Figure S56. Time-resolved fluorescence decay (blue) of HL^{NHBn} and the instrument response function (IRF, red) in a frozen glassy matrix of 2MeTHF at 77 K, including the residuals (λ_{ex} = 376 nm, λ_{obs} = 475 nm).
Figure S57. Dihedral angles Cl–Ni–N–C(1) and Cl–Ni–N–C(2), and bond angle Cl–Ni–C for the constraint optimized structures of [Ni(L^{NHBn})Cl] along the Ni–N coordinate.
Figure S58. Dihedral angles Cl–Ni–N–C(1) and Cl–Ni–N–C(2), and bond angle Cl–Ni–C for the constraint optimized structures of [Ni(L^{NHPh})Cl] along the Ni–N coordinate.
Figure S59. Main contributing orbitals to the $S_1 \rightarrow S_0$, $S_4 \rightarrow S_0$, and $T \rightarrow S_0$ of [Ni(L^{NHPh})Cl] from TDA calculations as detailed in Table S16.
Figure S60. Main contributing orbitals to the $S_1 \rightarrow S_0$, $S_4 \rightarrow S_0$, and $T_5 \rightarrow S_0$ of [Ni(L^{NHBn})Cl] from TDA calculations as detailed in Table S17.
Figure S61. 3D heat map of [Ni(L^{NHBn})Cl] at 298 K obtained by cryostat supported fs-TAS measurements in THF and evolution associated spectrum.
Figure S62. 3D heat map of [Ni(L^{NHPh})Cl] at 298 K obtained by cryostat supported fs-TAS measurements in THF and evolution associated spectrum.
Figure S63. 3D heat map of [Ni(L^{NHPh})Cl] at 298 K obtained by cryostat supported ns-TAS measurements in 2MeTHF. Evolution associated spectra of the ns-TA data of [Ni(L^{NHPh})Cl] with a lifetime of 722 ps.
Figure S64. 3D heat map of [Ni(L^{NHPh})Cl] at 150 K obtained by cryostat supported ns-TAS measurements in 2MeTHF. Evolution associated spectra of the ns-TA data of [Ni(L^{NHPh})Cl] with a lifetime of 10.1 ns.
Figure S65. 3D heat map of [Ni(L^{NHBn})Cl] at 298 K obtained by cryostat supported ns-TAS measurements in 2MeTHF. Evolution associated spectra (right) of the ns-TA data of [Ni(L^{NHBn})Cl] with a lifetime of 1.94 ns.
Figure S66. 3D heat map of [Ni(L^{NHBn})Cl] at 180 K obtained by cryostat supported ns-TAS measurements in 2MeTHF. Evolution associated spectra of the ns-TA data of [Ni(L^{NHBn})Cl] with a lifetime of 500 fs. The fit was not successful because of the high intensity of the instrument response function (IRF).
Figure S67. 3D heat map of ClL^{NHPh} at 298 K obtained by ns-TAS measurements in 2MeTHF. Evolution associated spectra of the ns-TA data of ClL^{NHPh} with a lifetime of 8.3 ns.
Figure S68. 3D heat map of ClL^{NHBn} at 298 K obtained by ns-TAS measurements in 2MeTHF. A fit was not possible due to the lack of signal.

Supplementary Tables

Table S1. Crystal data and structure refinement for [Ni(N^C^N)Cl] (N^C^N = L^{NHPh} and L^{NHBn}) complexes.

Table S2. Experimental and DFT-calculated geometries for [Ni(N^C^N)Cl] (N^C^N = L^{NHPh} or L^{NHBn}) and [Ni(dpb)Cl].

Table S3. Selected experimental structural data for [Ni(L^{NHBn})Cl].

Table S4. DFT-calculated geometries of the [Ni(N^C^N)Cl] (N^C^N = L^{NHPh} or L^{NHBn}).

Table S5. Selected DFT-calculated geometries for the [Ni(N^C^N)X] complexes in the S_0 ground state.

Table S6. Redox potentials of the complexes [Ni(N^C^N)Cl].

Table S7. DFT-calculated compositions (%) of frontier MOs in the S_0 ground state for [Ni(L^{NHPh})Cl].

Table S8. DFT-calculated compositions (%) of frontier MOs in the T_1 excited state for [Ni(L^{NHPh})Cl].

Table S9. DFT-calculated compositions (%) of frontier MOs in the S_0 ground state for [Ni(L^{NHBn})Cl].

Table S10. DFT-calculated compositions (%) of frontier MOs in the T_1 excited state for [Ni(L^{NHBn})Cl].

Table S11. Selected UV-vis absorption maxima of the complexes [Ni(N^C^N)Cl].

Table S12. Selected TD-DFT-calculated vertical $S_0 \rightarrow S_n$ transitions for [Ni(L^{NHPh})Cl].

Table S13. Selected TD-DFT-calculated vertical $S_0 \rightarrow S_n$ transitions for [Ni(L^{NHBn})Cl].

Table S14. Excited state lifetimes of the ligand precursors ClL^{NHPh} and ClL^{NHBn} and the protonated ligands HL^{NHPh}, and HL^{NHBn}.

Table S15. Characterization of selected TDA-calculated vertical excited states for the optimized ground state geometry of [Ni(L^{NHPh})Cl] using PBE0/SDD.

Table S16. Characterization of selected TDA-calculated excited states for the optimized ground state geometry of [Ni(L^{NHBn})Cl] using PBE0/SDD.

Table S17. Characterization of selected TDA-calculated excited states for the optimized ground state geometry of [Ni(L^{NHPh})Cl] using PBE0/ZORA-DEF2-TZVP.

Table S18. Characterization of selected TDA-calculated excited states for the optimized ground state geometry of [Ni(L^{NHBn})Cl] using PBE0/ZORA-DEF2-TZVP.

Table S19. Radiative lifetimes from SOC-TDA calculations at the optimized ground state geometry of [Ni(L^{NHPh})Cl] in THF using PBE0/ZORA-DEF2-TZVP.

Table S20. Radiative lifetimes from SOC-TDA calculations at the optimized ground state geometry of [Ni(L^{NHBn})Cl] in THF using PBE0/ZORA-DEF2-TZVP.

Table S21. Calculated coordinates of the optimized singlet ground state structures of the complexes [Ni(N^C^N)Cl].

Table S22. Calculated coordinates of the optimized triplet excited state structures of the complexes [Ni(N^C^N)Cl].

References

Materials and Syntheses

1. Materials

The chemicals 1,3-dibromo-2-chlorobenzene (96%, BLD Pharmatech GmbH, Germany), Bis(pinacolato)diboron (B_2Pin_2) (98%, BLD Pharmatech GmbH, Germany), 2-amino-6-bromopyridine (98%, BLD Pharmatech GmbH, Germany), phenylboronic acid (98%, BLD Pharmatech GmbH, Germany), $[Pd(PPh_3)_2Cl_2]$ (98%, BLD Pharmatech GmbH, Germany), $[Pd(PPh_3)_4]$ (99%, Thermo Scientific Chemicals, Germany), KO*t*Bu (97%, Thermo Scientific Chemicals, Germany), KOAc (98%, Alfa Aesar, Germany), $[Cu(acac)_2]$ (97%, Sigma-Aldrich, Germany), K_2CO_3 (99%, Alfa Aesar, Germany), Na_2SO_4 (99%, Thermo Scientific Chemicals, Germany), *n*-Buli (2.5 M in hexanes; Thermo Scientific Chemicals, Germany), $[Ni(acac)_2]$ (96%, Thermo Scientific Chemicals, Germany), 1,5-cyclooctadiene (99%, Acros Organics, Germany), DIBAl-H (1.0 M in hexanes; Acros Organics, Germany) were purchased and used without further purification.

Dry THF was obtained from distillation over sodium/potassium alloy. $[Ni(COD)_2]$ (COD = 1,5-cyclooctadiene) [1] and the protonated ligands HL^{NHBn} and HL^{NHPh} [2] were obtained as previously reported.

2. Syntheses and Characterization

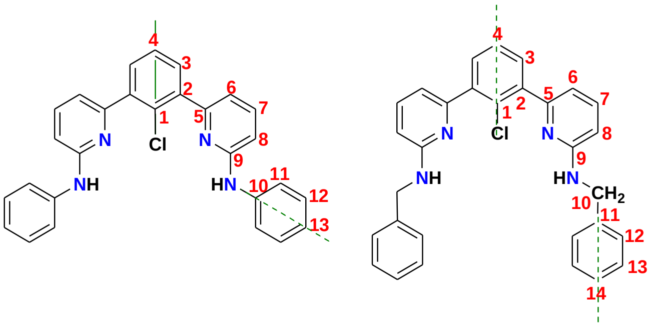

Scheme S1. Numbering schemes used for NMR assignments.

2.1 Synthesis of the Ligand Precursor ClL^{NHPh}.

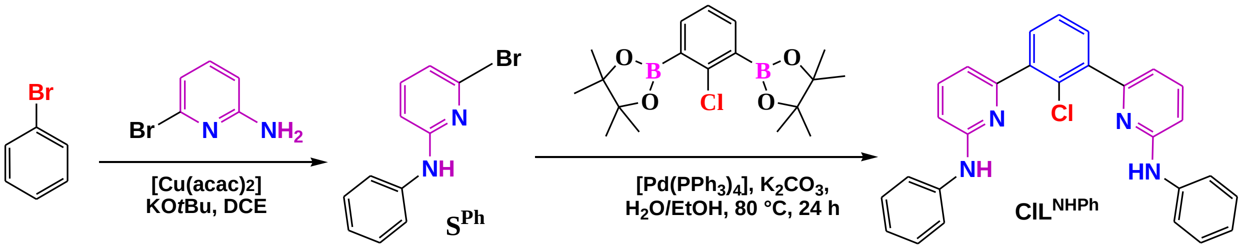

Scheme S2. Synthetic pathway for S^{Ph} and ClL^{NHPh}.

Synthesis of 2,2′-(2-chloro-1,3-phenylene)bis(4,4,5,5-tetramethyl-1,3,2-dioxaborolane). To a solution of 1,3-dibromo-2-chlorobenzene (1.0 mmol, 0.270 g), B_2Pin_2 (3.0 mmol, 0.761 g), and KOAc (5 mmol, 0.491 g), in 6 mL 1,4-dioxane was added $[Pd(PPh_3)_2Cl_2]$ (6 mol%, 0.042 g) under inert condition. After three freeze–thaw cycles to remove trace amount of oxygen, the reaction mixture stirred at 102 ˚C for 12 h. The mixture was cooled down and 10 mL EtOAc was added to quench the reaction. The organic phase was washed with brine solution (3 × 20 mL) and dried over anhydrous Na_2SO_4. After evaporation of the solvent, crude product was purified by column chromatography using CH_2Cl_2 as an eluent to give white crystals. Yield: 0.272 g (0.75 mmol, 75%). ^1H NMR (300 MHz, acetone-d_6, 298 K): δ /ppm = 7.68 (d, J = 7.4 Hz, 2H), 7.29 (t, J = 7.4 Hz, 1H), 1.35 (s, 24H).

Synthesis of S^{Ph}. To a solution of 2-amino-6-bromopyridine (17.34 mmol, 3.0 g), and KO*t*Bu (19.0 mmol, 2.14 g) in 1,2-dichlorethane (50 mL) were added phenylboronic acid (27.74 mmol, 3.4 g) and Cu(II) acetyl acetonate (17.34 mmol, 4.54 g). The solution was heated under reflux (80 °C) for 1 d. Then the reaction mixture was washed with brine (3 × 50 mL). The combined organic layers were dried over Na_2SO_4 and the product (S^{Ph}) was purified by column chromatography with *n*-Hex:EtOAc (9:1 v/v). Yield: 0.660 g (2.6 mmol, 15%). ^1H NMR

(300 MHz, acetone-d_6, 298 K): δ /ppm = 8.43 (s, 1H, NH), 7.65 (dd, *J* = 8.7, 1.1 Hz, 2H), 7.38 (dd, *J* = 8.2, 7.5 Hz, 1H), 7.34–7.26 (m, 2H), 7.02–6.95 (m, 1H), 6.88 (dd, *J* = 7.5, 0.6 Hz, 1H), 6.81 (d, *J* = 8.2 Hz, 1H).

Synthesis of ClL^{NHPh}. 2,2′-(2-chloro-1,3-phenylene)bis(4,4,5,5-tetramethyl-1,3,2-dioxaborolane) (0.958 g, 2.63 mmol), S^{Ph} (0.935 g, 3.75 mmol), [Pd(PPh$_3$)$_4$] (0.130 g, 3 mol%), and K_2CO_3 (1.04 g, 7.51 mmol) were mixed in 40 mL H_2O/EtOH (1:1, degassed). The solution was stirred for 24 h at 80 °C under an argon atmosphere. After allowing the reaction mixture to cool to room temperature, the mixture was extracted with CH_2Cl_2. The extract was washed with brine (2 × 40 mL), separated and dried over anhydrous Na_2SO_4. The crude product was purified by column chromatography with *n*-Hex:EtOAc (7:3 v/v). Yield: 0.547 g (1.22 mmol, 65%). ^{1}H NMR (600 MHz, DMSO-d_6, 298 K): δ /ppm = 9.16 (s, 2H, NH), 7.72 (dd, *J* = 8.6, 1.0 Hz, 4H, H11), 7.68 (dd, *J* = 8.2, 7.5 Hz, 2H, H7), 7.60–7.51 (m, 3H, H3 and H4), 7.25–7.20 (m, 4H, H12), 6.99 (d, *J* = 7.2 Hz, 2H, H6), 6.90–6.83 (m, 4H, H8 and H13). ^{13}C NMR (151 MHz, DMSO-d_6, 298 K): δ /ppm = 155.37 (C9), 154.63 (C5), 141.60 (C10), 140.69 (C2), 137.44 (C7), 130.83 (C3), 129.30 (C1), 128.61 (C12), 126.93 (C4), 120.41 (C13), 117.98 (C11), 115.01 (C6), 109.62 (C8). HR-ESI MS(+): calcd. for $[C_{28}H_{22}ClN_4]^+$ m/z = 449.15275; found m/z = 449.15248 $[M+H]^+$. Elemental analysis calcd. (%) for $C_{28}H_{21}ClN_4$, M = 448.95 g mol^{-1}: C, 74.91; H, 4.71; N, 12.48. Found C, 74.23; H, 4.68; N, 12.49.

2.2 Synthesis of the Ligand Precursor ClL^{NHBn}.

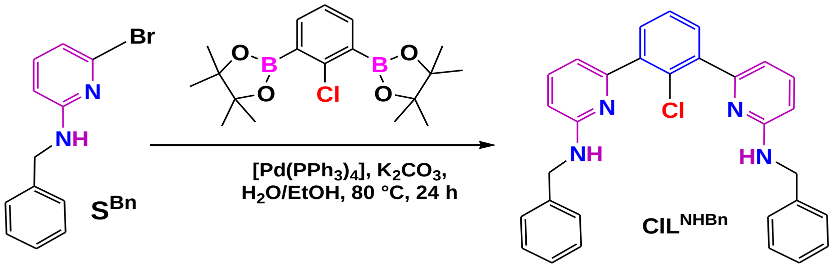

Scheme S3. Synthetic pathway for ClL^{NHBn}.

Synthesis of ClL^{NHBn}. 2,2′-(2-chloro-1,3-phenylene)bis(4,4,5,5-tetramethyl-1,3,2-dioxaborolane) (1.95 g, 5.34 mmol), S^{Bn} (2.0g, 7.6 mmol), [Pd(PPh$_3$)$_4$] (0.264 g, 3 mol%), and K_2CO_3 (2.1 g, 15.2 mmol) were mixed and dissolved in 50 mL H_2O/EtOH (1:1, degassed). The solution was stirred for 24 h at 80 °C under an argon atmosphere. After allowing the reaction mixture to cool to ambient temperature, the mixture was extracted with CH_2Cl_2. The extract was washed with brine (2 × 40 mL), separated and dried over anhydrous Na_2SO_4. The crude product was pvcurified by column chromatography with *n*-Hex:EtOAc (6:4 v/v). Yield: 1.17 g (2.5 mmol, 65%). ^{1}H NMR (600 MHz, DMSO-d_6, 298 K): δ /ppm = 7.46 (dd, *J* = 8.3, 7.3 Hz, 2H, H7), 7.41–7.38 (m, 3H, H4 and H3), 7.37–7.34 (m, 4H, H12), 7.33–7.27 (m, 4H, H13), 7.21 (t, *J* = 7.3 Hz, 2H, H14), 7.15 (t, *J* = 6.0 Hz, 2H, NH), 6.70 (dd, *J* = 7.3, 0.5 Hz, 2H, H6), 6.52 (d, *J* = 8.3 Hz, 2H, H8), 4.49 (d, *J* = 6.0 Hz, 4H, H10). ^{13}C NMR (151 MHz, DMSO-d_6, 298 K): δ /ppm = 158.26 (C9), 154.92 (C5), 140.86 (C11), 140.61 (C2), 136.79 (C7), 130.52 (C3), 129.32 (C1), 128.15 (C13), 127.39 (C12), 126.51 (C14), 126.37 (C4), 112.45 (C6), 107.00 (C8), 44.22 (C10). HR-ESI MS(+): calcd. for $[C_{30}H_{25}ClN_4]^+$ m/z = 477.18405; found m/z = 477.18403 $[M+H]^+$. Elemental analysis calcd. (%) for $C_{30}H_{25}ClN_4$, M = 477.0 g mol^{-1}: C, 75.54; H, 5.28; N, 11.75. Found C, 75.51; H, 5.27; N, 11.75.

2.3 Synthesis of [Ni(N^C^N)Cl] Complexes – General Description

Under an inert atmosphere, 1.0 eq. of the halogenated derivatives of the ligands ClL^{NHPh} or ClL^{NHBn} was dissolved in 8 mL of dry THF. The reaction mixture was transferred to an ice bath at 0°C and 1.3 eq. of [Ni(COD)$_2$] was added. The solution turned from yellow to dark orange with red-orange precipitation during 2 h. Then the solvent was removed and the residue was washed with dry MeCN to remove unreacted ligand and dried under vacuum.

[Ni(L^{NHPh})Cl]. From [Ni(COD)$_2$] 0.29 g (0.83 mmol) and ClL^{NHPh} 0.288 g (0.64 mmol). Yield 0.195 g (0.38 mmol, 59%) deep orange solid. ^{1}H NMR (300 MHz, DMSO-d_6, 298 K): δ /ppm = 9.48 (s, 2H, NH), 7.66 (t, *J* = 7.9 Hz, 2H, H7), 7.45–7.33 (m, 6H, H3 and H12), 7.29–7.14 (m, 7H, H6 and H11 and H4), 7.10 (t, *J* = 7.4 Hz, 2H, H13),

6.73 (d, *J* = 8.5 Hz, 2H, H8). HR-ESI MS(+): calcd. for $[C_{28}H_{20}N_4Ni]^+$ m/z = 470.10300; found m/z = 470.10360 [M-H-Cl]$^+$. Elemental analysis calcd. (%) for $C_{28}H_{21}ClN_4Ni$, M = 507.64 g mol^{-1}: C, 66.25; H, 4.17; N, 11.04. Found C, 66.21; H, 4.15; N, 11.07.

[Ni(L^{NHBn})Cl]. From $[Ni(COD)_2]$ 0.242 g (0.88 mmol) and ClL^{NHBn} 0.322 g (0.68 mmol). Yield 0.244 g (0.46 mmol, 67%) deep orange solid. ^{1}H NMR (600 MHz, DMSO-*d*$_6$, 298 K): δ /ppm = 8.12 (t, *J* = 5.6 Hz, 2H, NH), 7.54 (t, *J* = 8.0 Hz, 2H, H7), 7.45 (d, *J* = 7.4 Hz, 4H, H12), 7.37–7.31 (m, 4H, H13), 7.30–7.22 (m, 4H, H14 and H3), 7.09 (t, *J* = 7.6 Hz, 1H, H4), 6.98 (d, *J* = 7.2 Hz, 2H, H6), 6.26 (d, *J* = 8.5 Hz, 2H, H8), 4.42 (d, *J* = 6.2 Hz, 4H, H10). HR-ESI MS(+): calcd. for $[C_{30}H_{24}N_4Ni]^+$ m/z = 498.13494; found m/z = 498.13490 [M-H-Cl]$^+$. Elemental analysis calcd. (%) for $C_{30}H_{25}ClN_4Ni$, M = 535.69 g mol^{-1}: C, 67.26; H, 4.70; N, 10.46. Found C, 67.18; H, 4.71; N, 10.47.

Supporting Figures

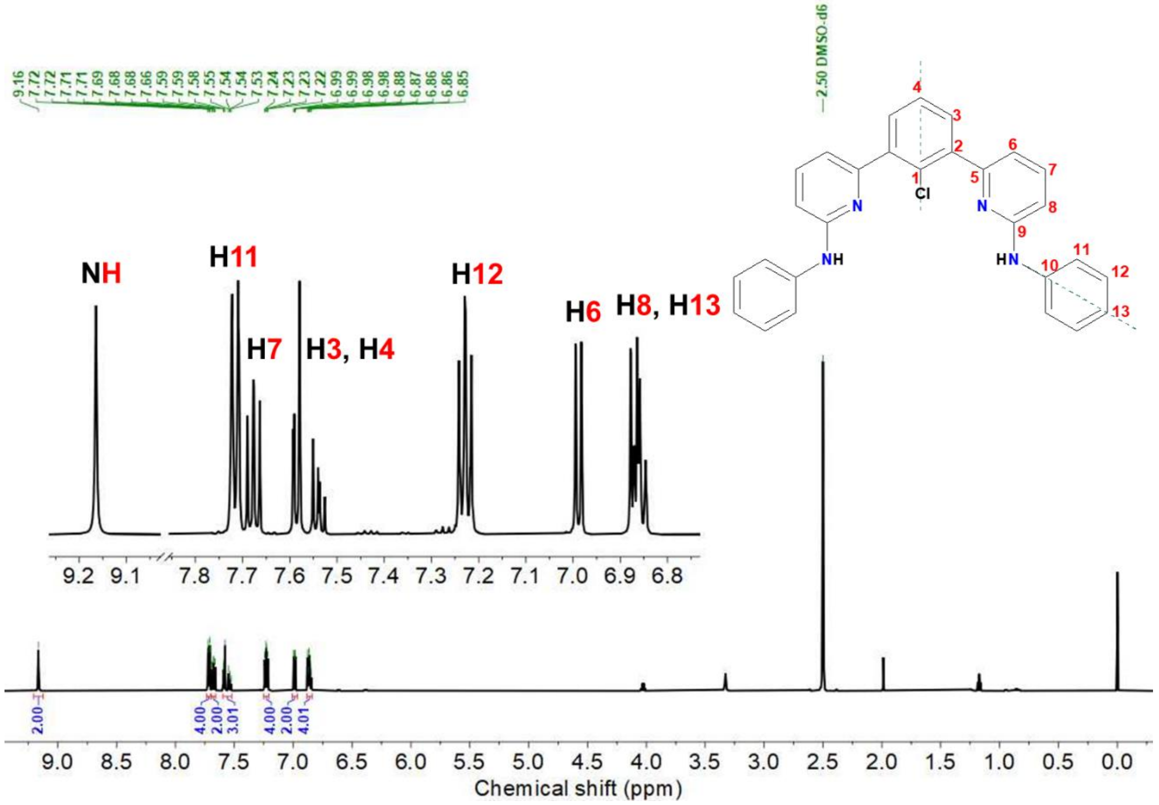

Figure S1. 600 MHz ^{1}H NMR spectrum of ClL^{NHPh} in DMSO-d_6 at RT.

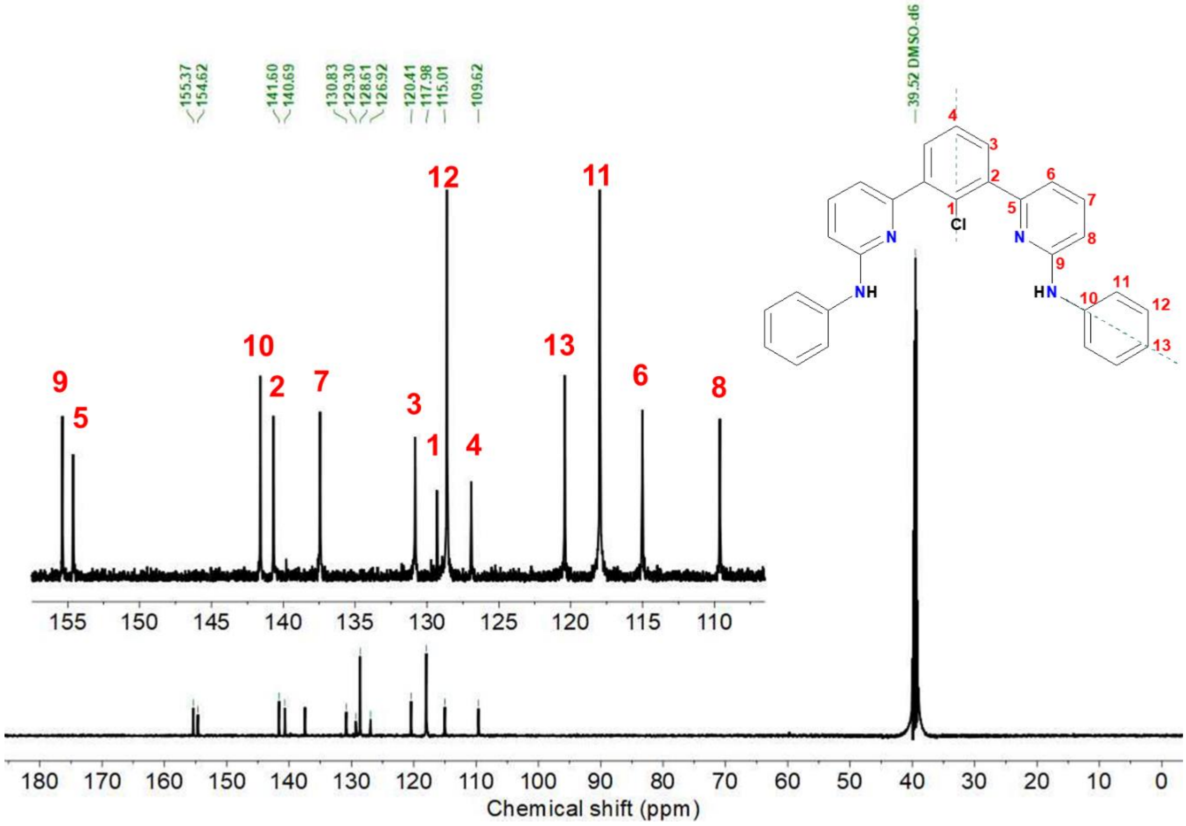

Figure S2. 151 MHz ^{13}C NMR spectrum of ClL^{NHPh} in DMSO-d_6 at RT.

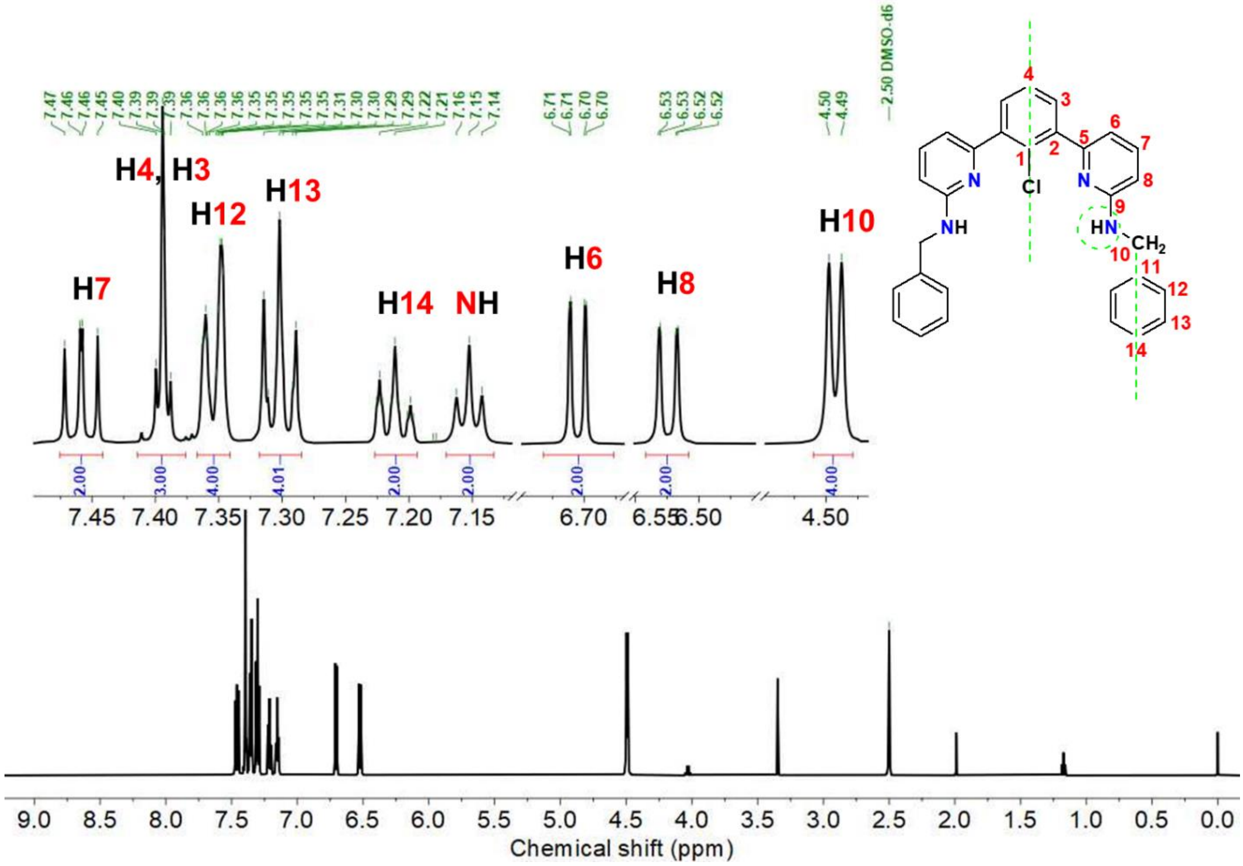

Figure S3. 600 MHz ^{1}H NMR spectrum of ClL^{NHBn} in DMSO-d_6 at RT.

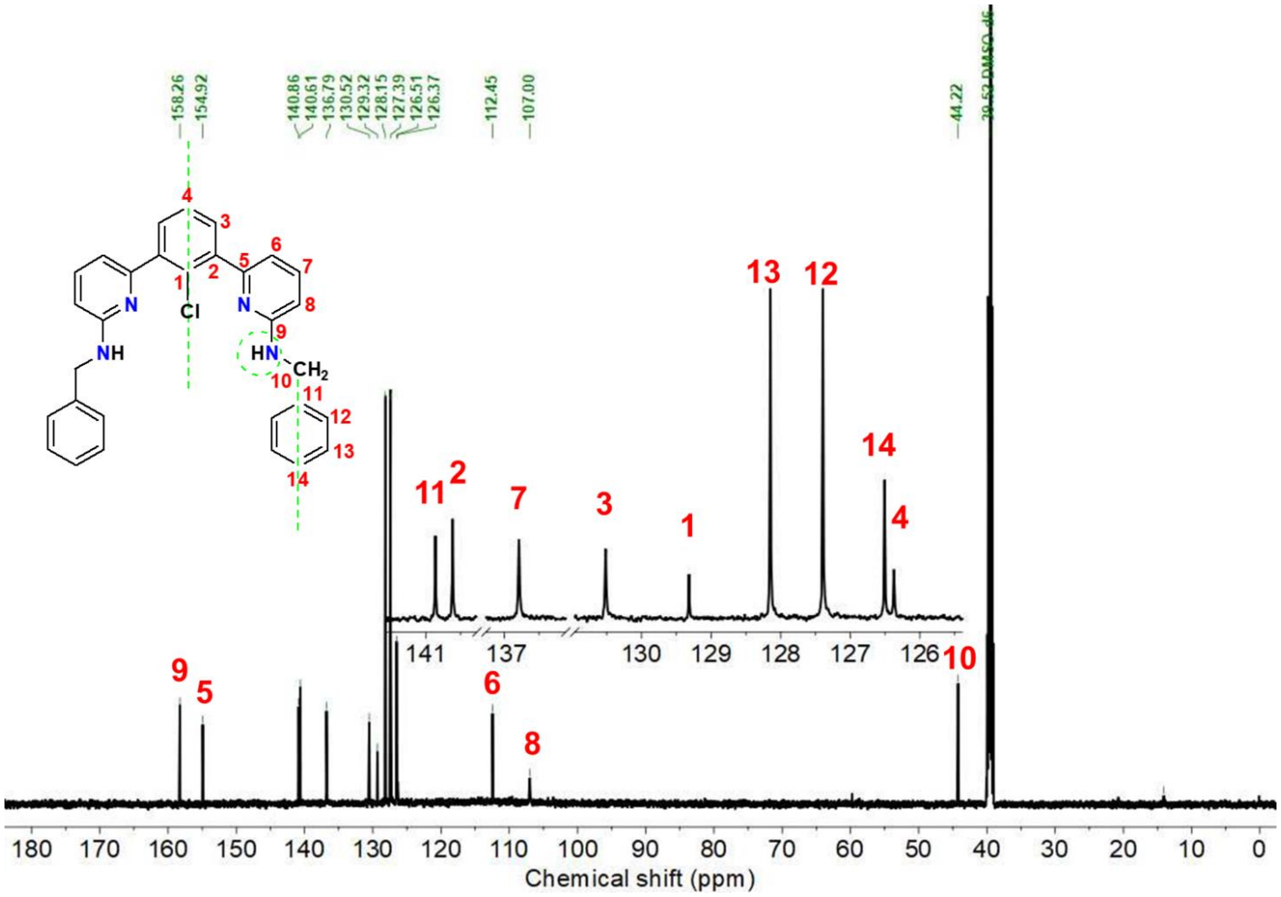

Figure S4. 151 MHz ^{13}C NMR spectrum of ClL^{NHBn} in DMSO-d_6 at RT.

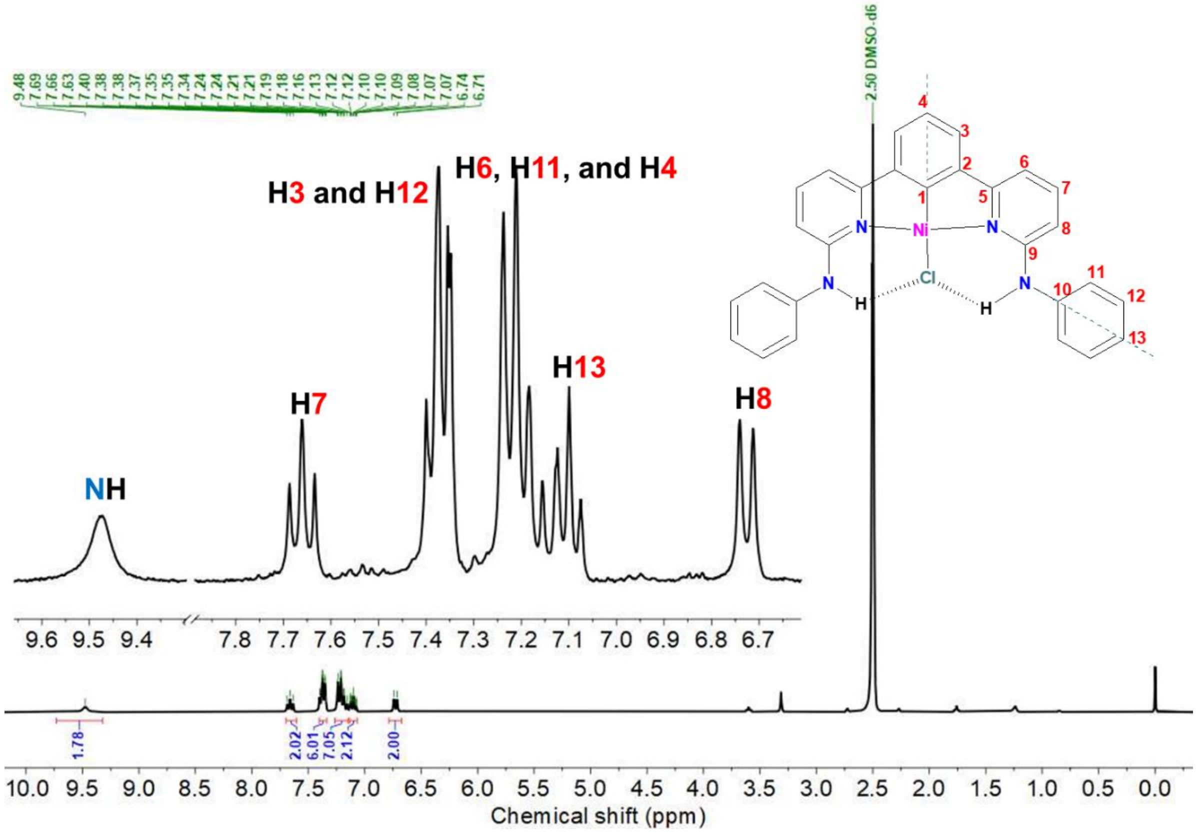

Figure S5. 300 MHz ^{1}H NMR spectrum of [Ni(L^{NHPh})Cl] in DMSO-d_6 at RT.

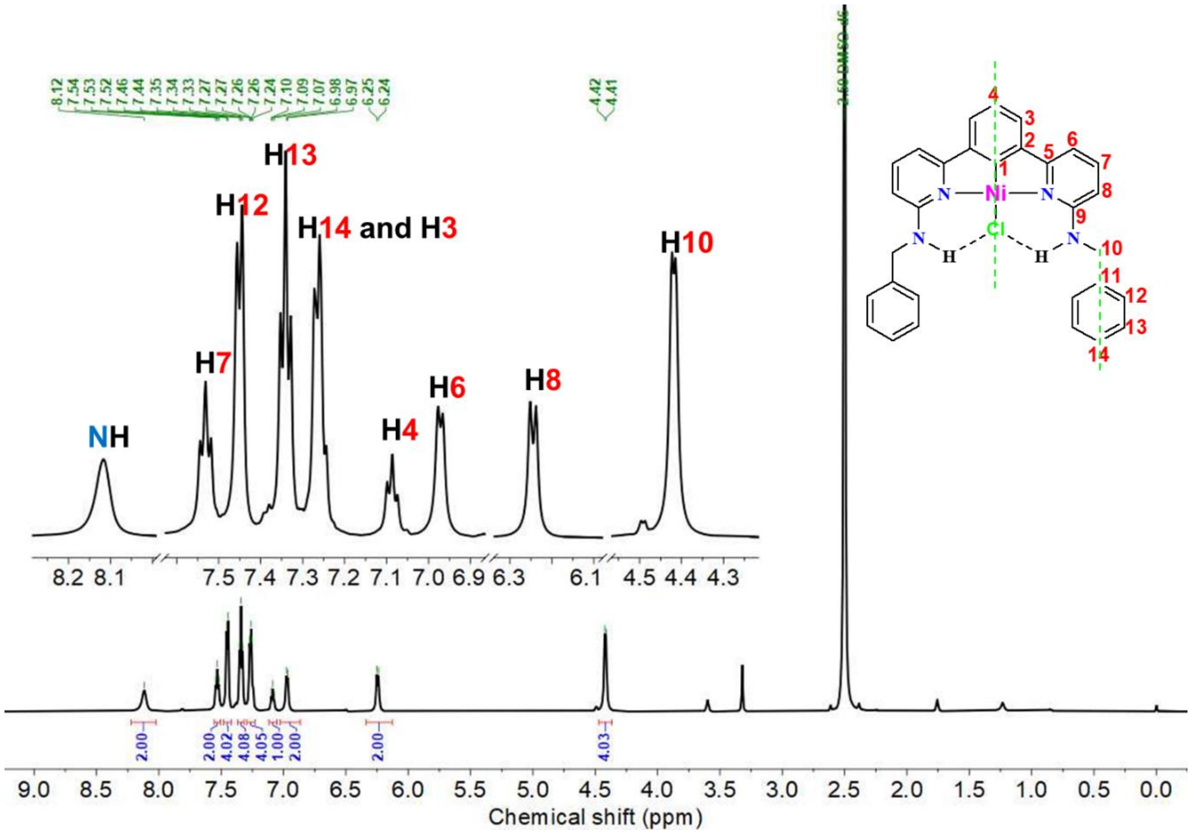

Figure S6. 600 MHz ^{1}H NMR spectrum of [Ni(L^{NHBn})Cl] in DMSO-d_6 at RT.

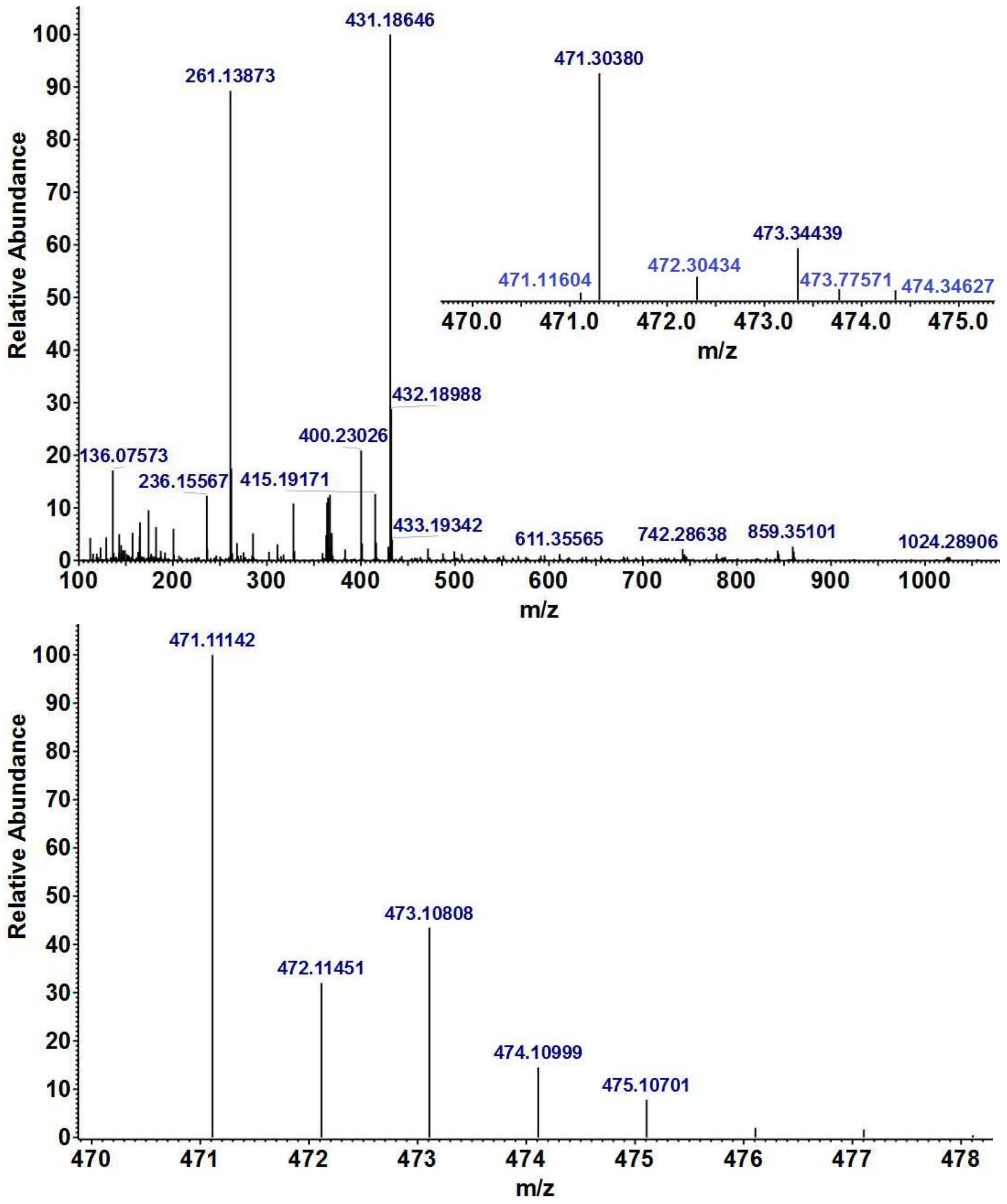

Figure S7. High-resolution ESI-MS(+) spectrum of [Ni(L^{NHPh})Cl] (top) and calculated pattern for $[C_{28}H_{21}N_4Ni]^+$ = $[M–Cl]^+$ (bottom).

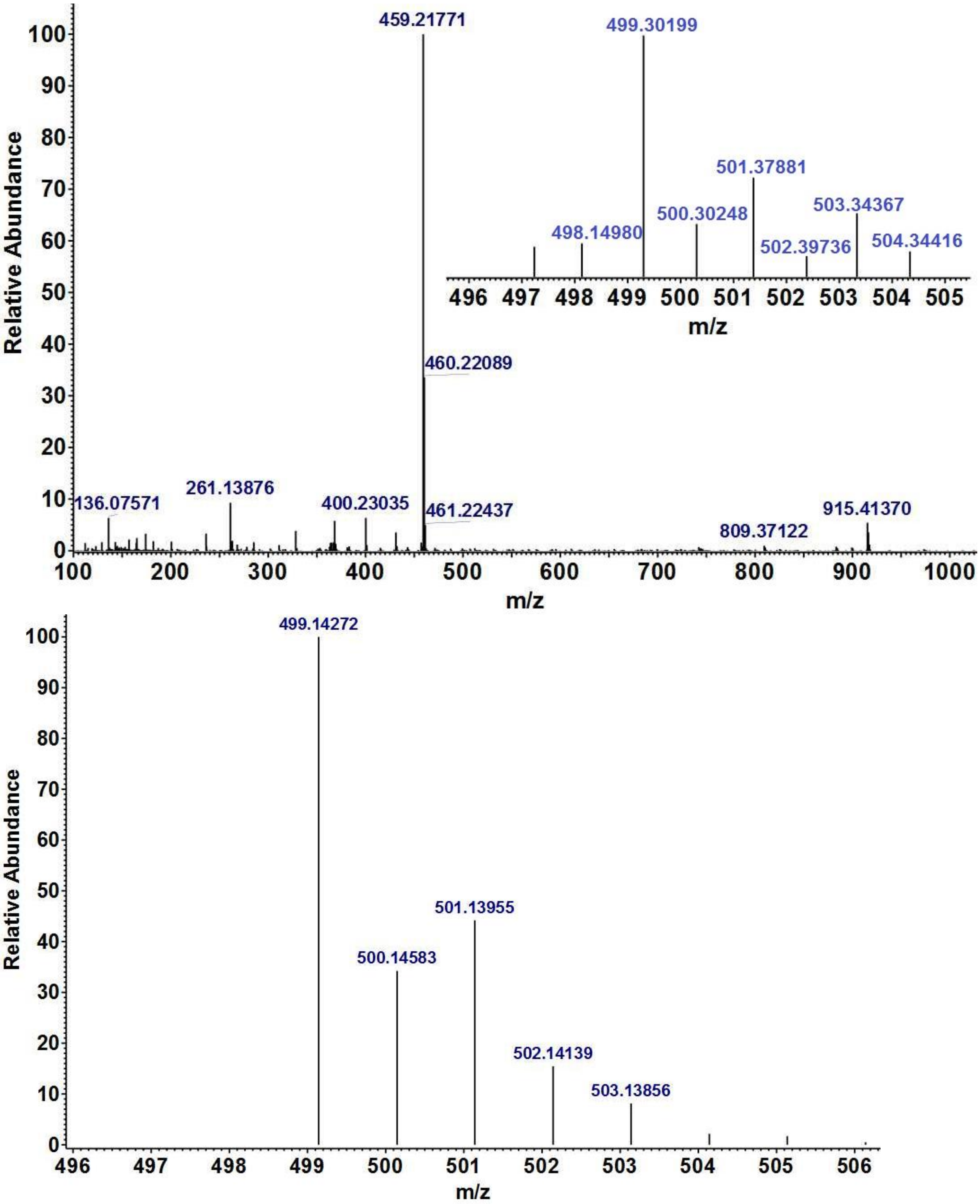

Figure S8. High-resolution ESI-MS(+) spectrum of [Ni(L^{NHBn})Cl] (top) and calculated pattern for $[C_{30}H_{25}N_4Ni]^+$ = $[M–Cl]^+$ (bottom).

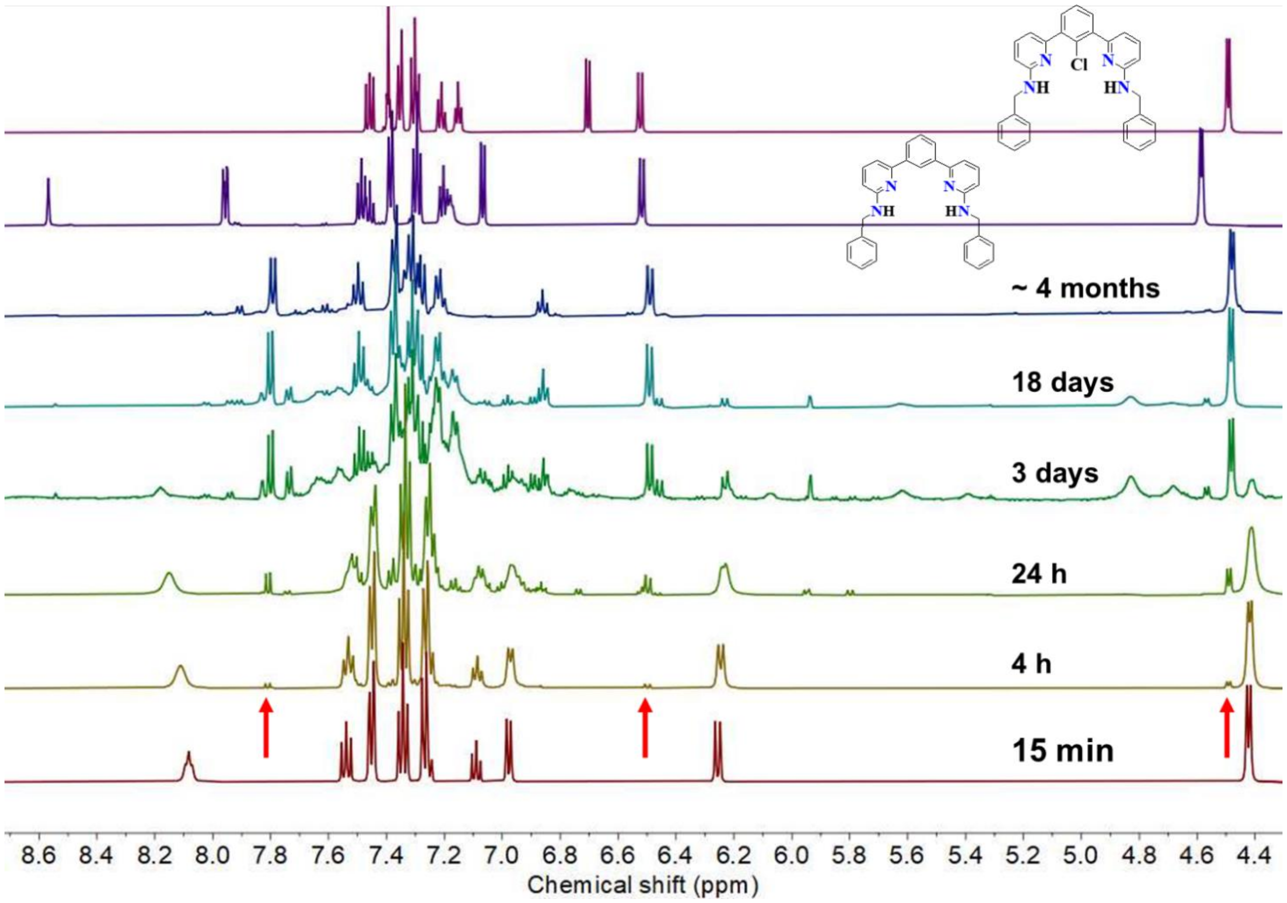

Figure S9. ^{1}H NMR spectra of [Ni(L^{NHBn})Cl] over time, alongside with ^{1}H NMR spectra of HL^{NHBn} (from Ref. 2) and ClL^{NHBn}, in DMSO-d_6 at RT.

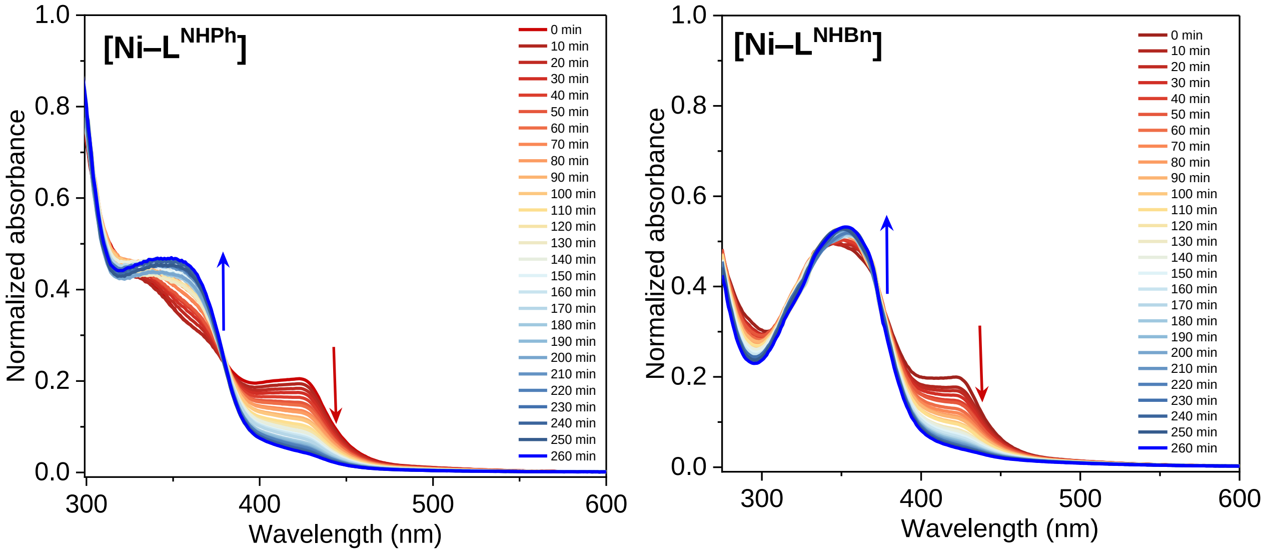

Figure S10. UV-vis absorption spectra of the [Ni(N^C^N)Cl] complexes over time in THF solution at 298 K.

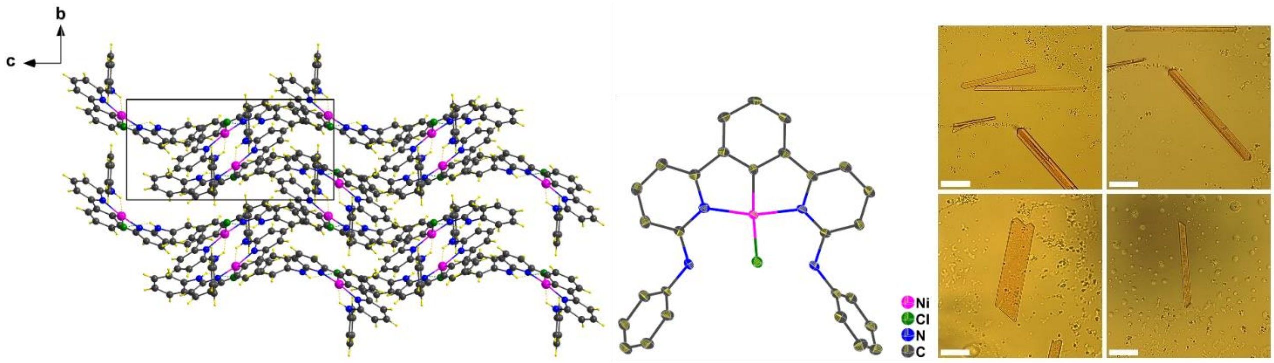

Figure S11. Crystal structure of [Ni(L^{NHPh})Cl] from single crystal X-ray diffractometry (XRD), viewed along the crystallographic *a* axis (left), molecular structure with atoms as 50% ellipsoids, H atoms omitted for clarity (center), and photoluminescence micrographs of the complex in the crystal state at 298 K, scale bar = 20 μm (right).

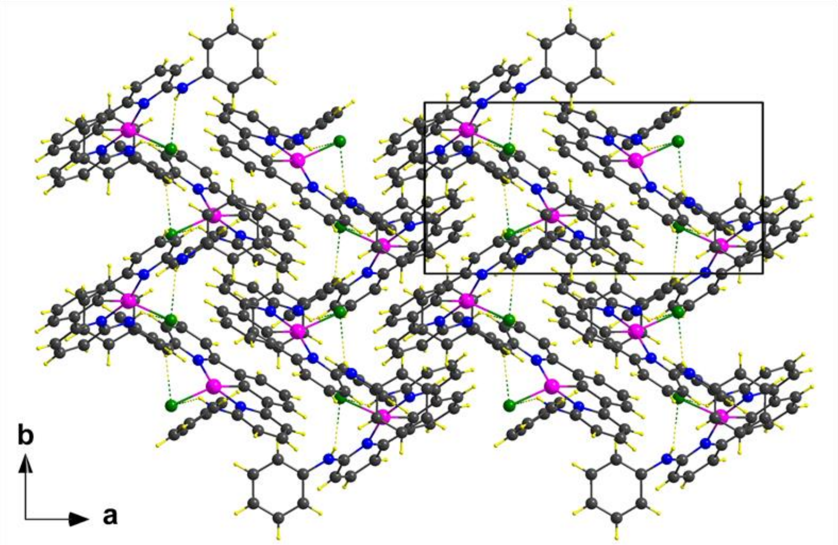

Figure S12. Crystal structure of [Ni(L^{NHPh})Cl] from single crystal X-ray diffractometry (XRD), viewed along the crystallographic *c* axis.

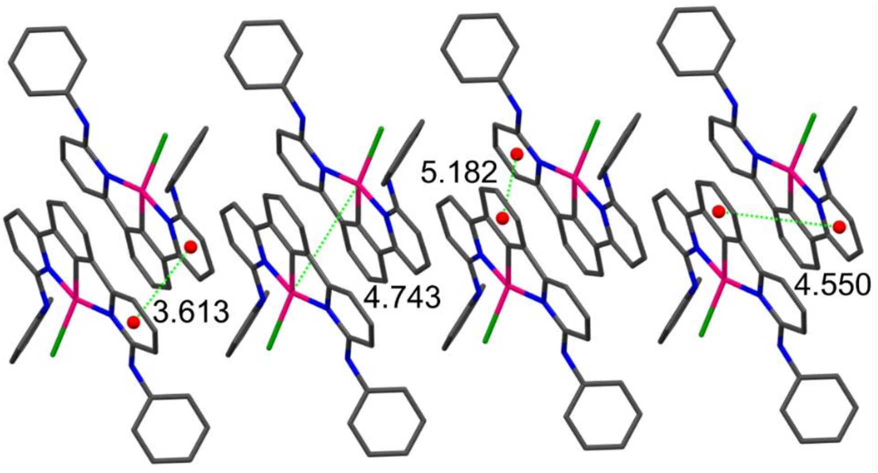

Figure S13. Intermolecular contacts and Ni···Ni distances (Å) in [Ni(L^{NHPh})Cl].

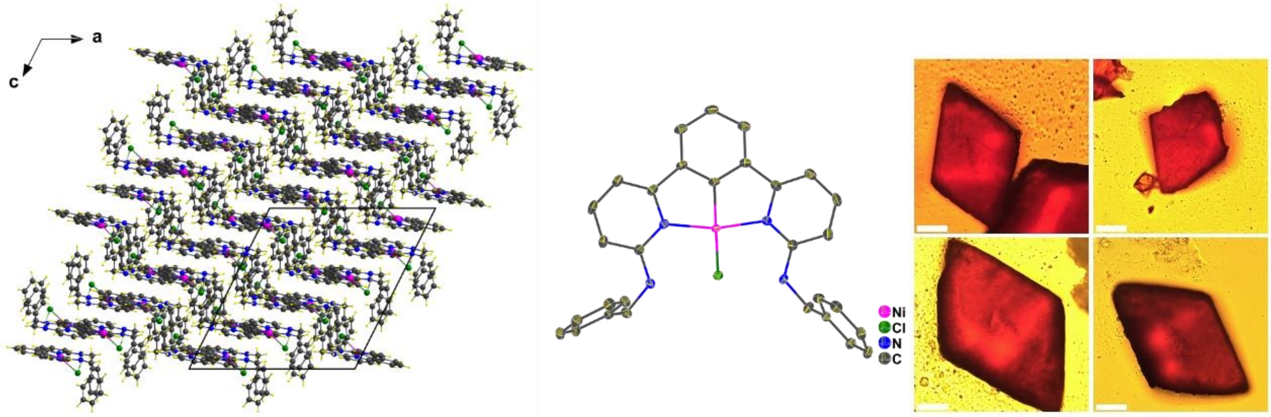

Figure S14. Crystal structure of [Ni(L^{NHBn})Cl] from single crystal XRD, viewed along the crystallographic *b* axis (left), molecular structure with atoms as 50% ellipsoids, H atoms omitted for clarity (center), and photoluminescence micrographs of the complex in the crystal state at 298 K, scale bar = 20 μm (right).

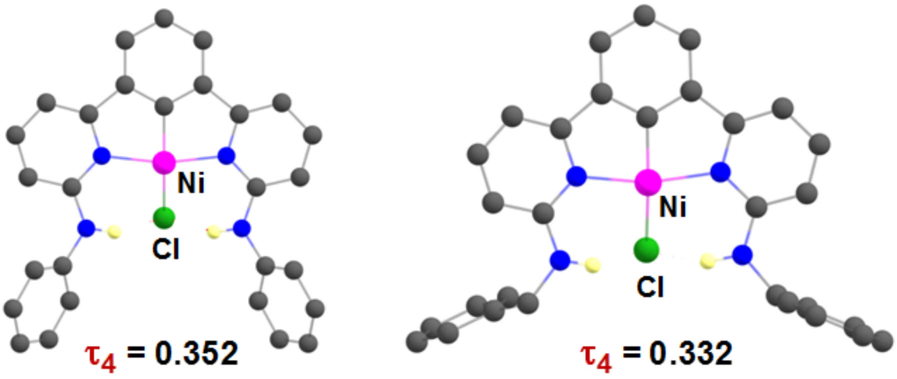

Figure S15. DFT-calculated molecular structures of [Ni(L^{NHPh})Cl] (left), [Ni(L^{NHBn})Cl] (right), in the S_0 ground state, featuring the deviation τ_4 from square planar coordination (for square planar, τ_4 = 0; for tetrahedral, τ_4 = 1).

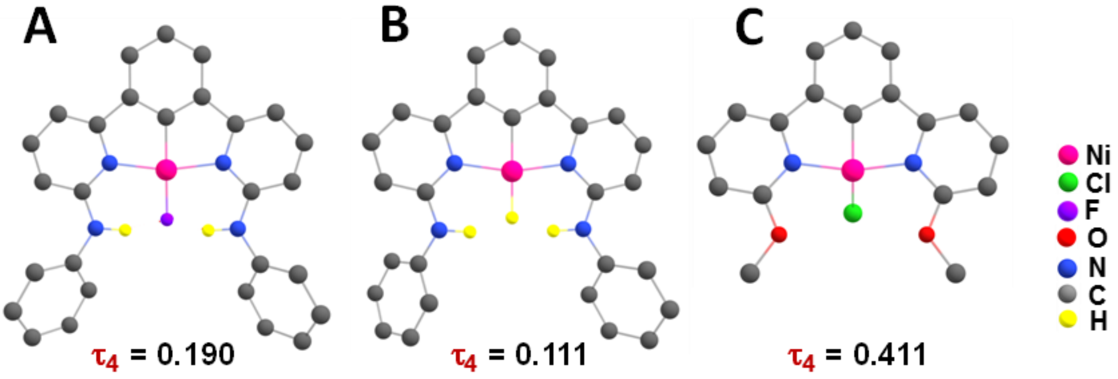

Figure S16. DFT-calculated molecular structures of [Ni(L^{NHPh})F] (A), [Ni(L^{NHPh})H] (B), and [Ni(L^{OMe})Cl] (C) in the S_0 ground state, featuring the deviation τ_4 from square planar coordination (for square planar, τ_4 = 0; for tetrahedral, τ_4 = 1).

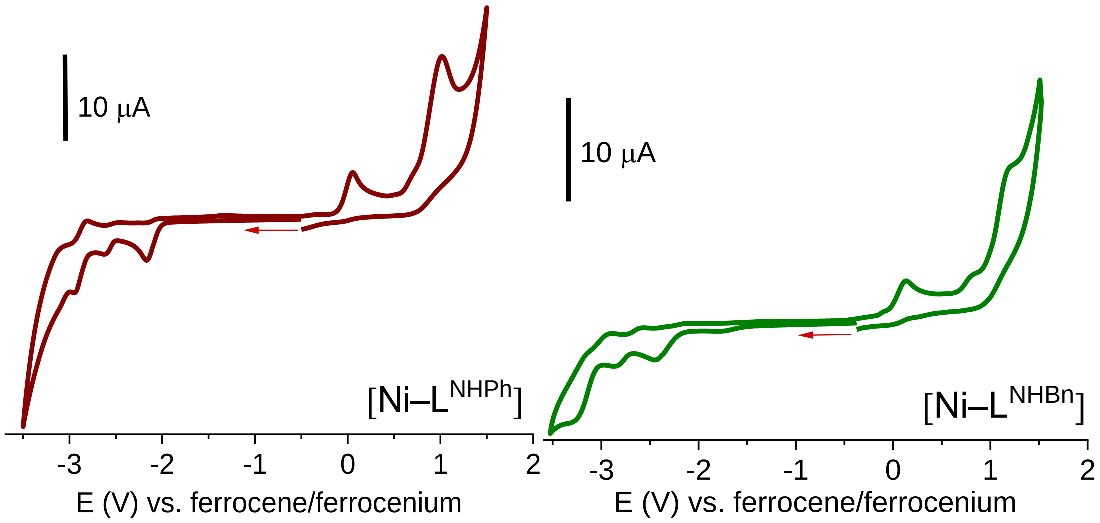

Figure S17. Cyclic voltammograms of the complexes [Ni(N^C^N)Cl] in *n*-Bu_4NPF_6/THF.

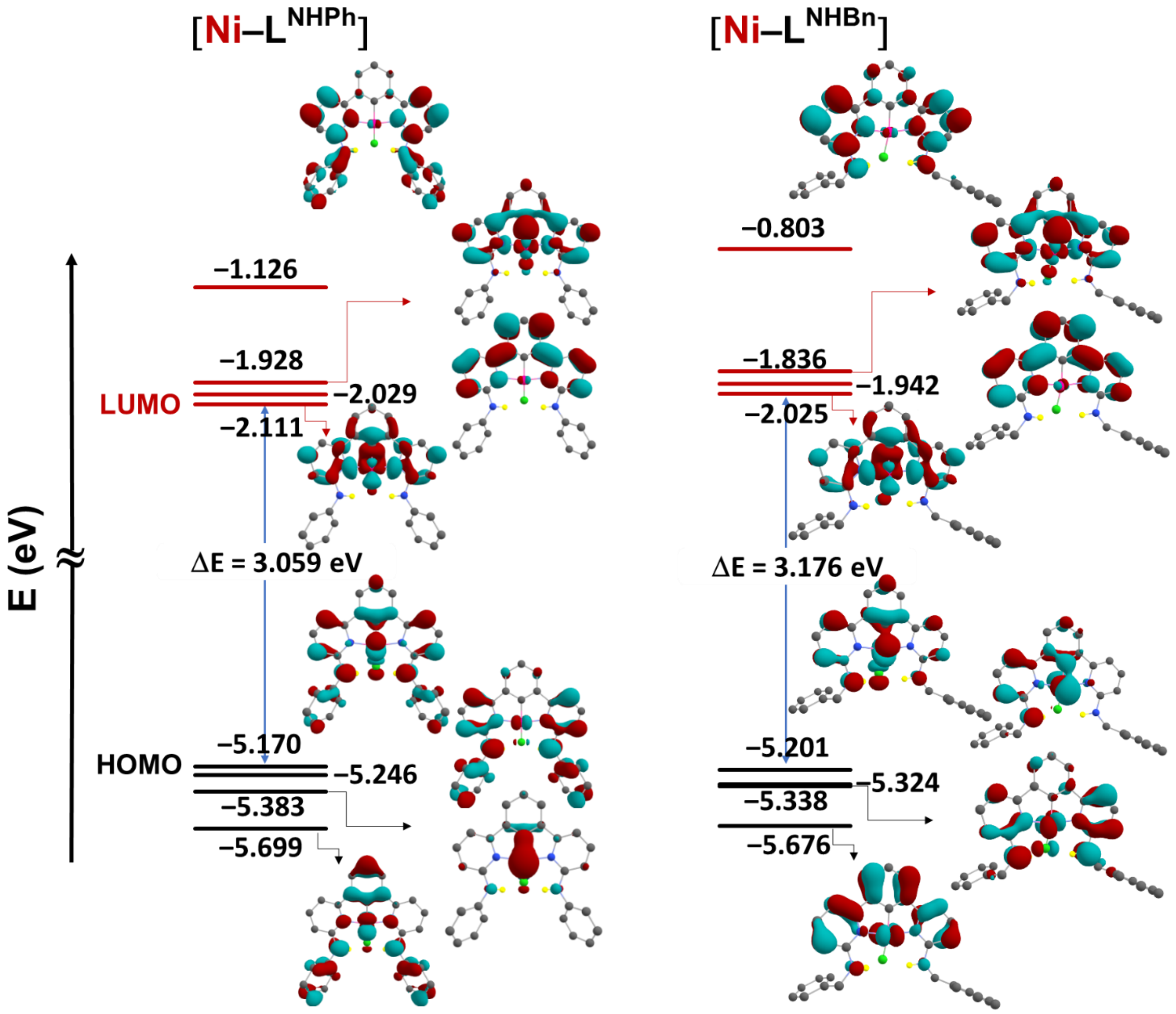

Figure S18. DFT-calculated energies of the highest occupied (HOMO, black) and lowest unoccupied (LUMO, red) molecular orbitals of [Ni(L^{NHPh})Cl] and [Ni(L^{NHBn})Cl].

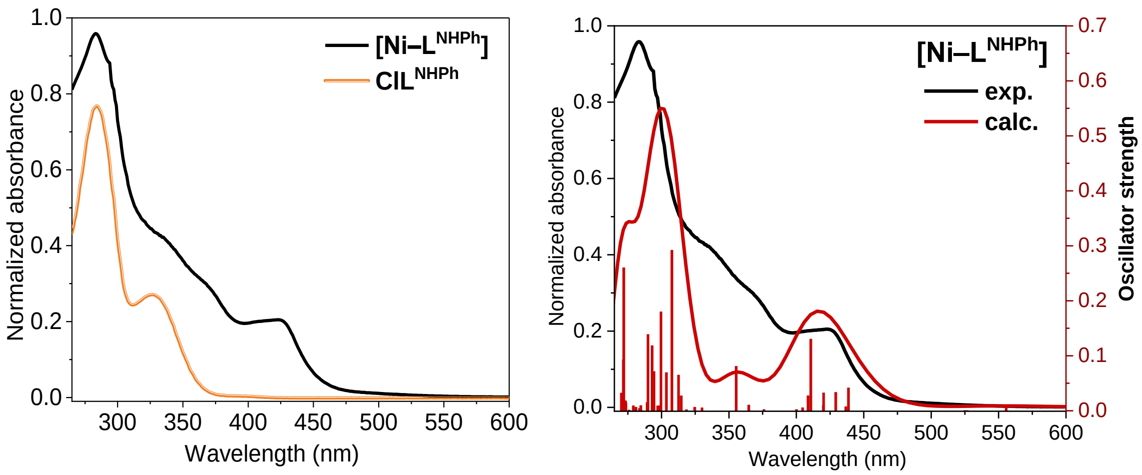

Figure S19. Experimental UV-vis absorption spectra of ClL^{NHPh}, and [Ni(L^{NHPh})Cl] measured in THF at 298 K with TD-DFT-calculated transitions (spectrum as line, individual transitions as sticks, in red).

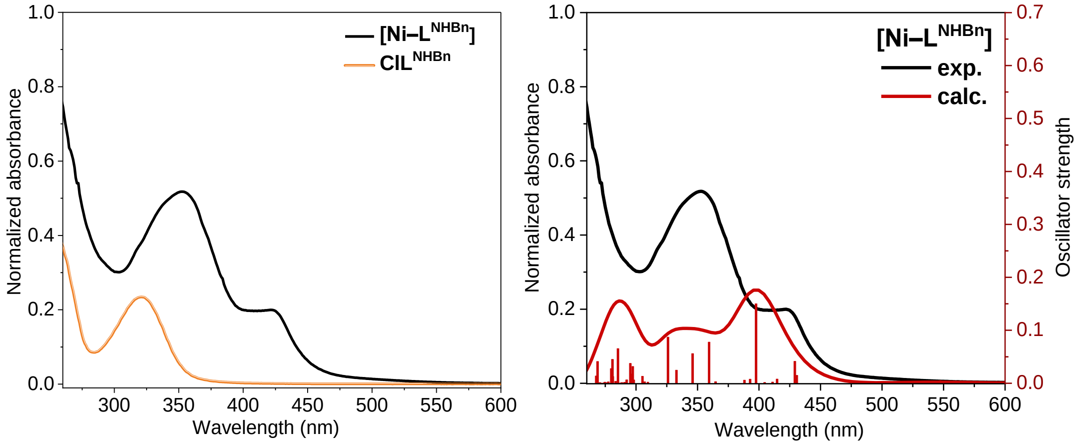

Figure S20. Experimental UV-vis absorption spectra of ClL^{NHBn}, and [Ni(L^{NHBn})Cl] measured in THF at 298 K with TD-DFT-calculated transitions (spectrum as line, individual transitions as sticks, in red).

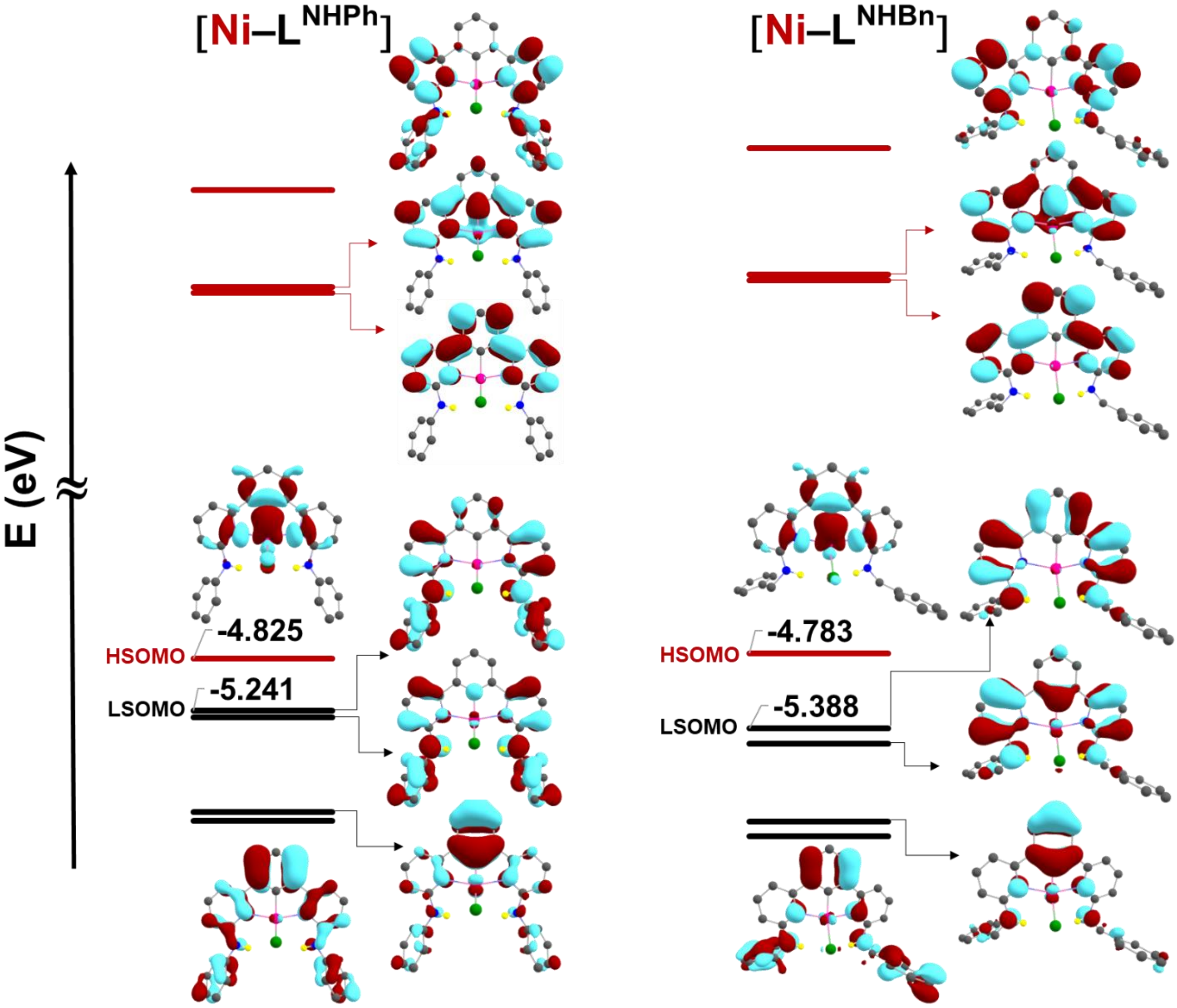

Figure S21. DFT-calculated energies of the lowest singly occupied (LSOMO, black) and highest singly occupied (HSOMO, red) molecular orbitals for [Ni(N^C^N)Cl], N^C^N = L^{NHPh} and L^{NHBn} .

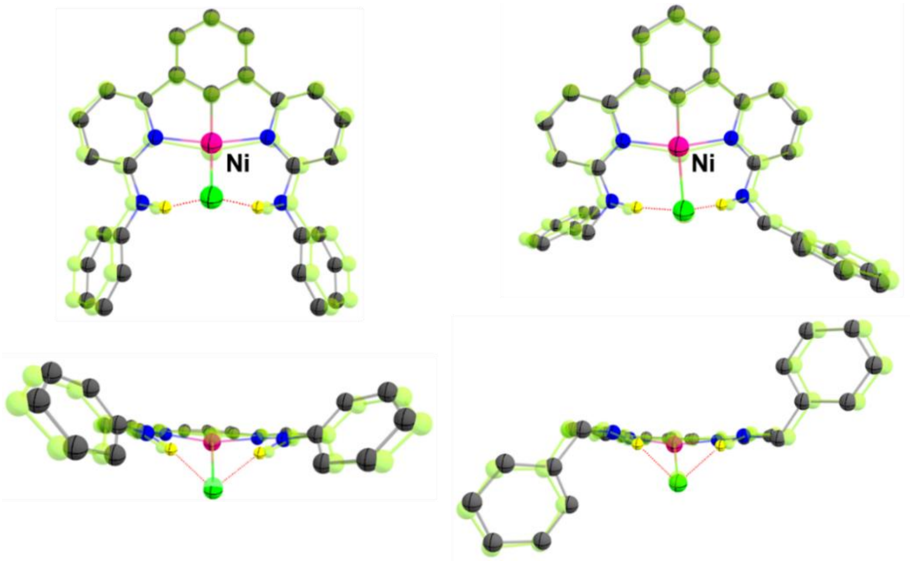

Figure S22. DFT-calculated geometries of [Ni(N^C^N)Cl] (N^C^N = L^{NHPh} and L^{NHBn}) in the S_0 (black) and T_1 (green) states.

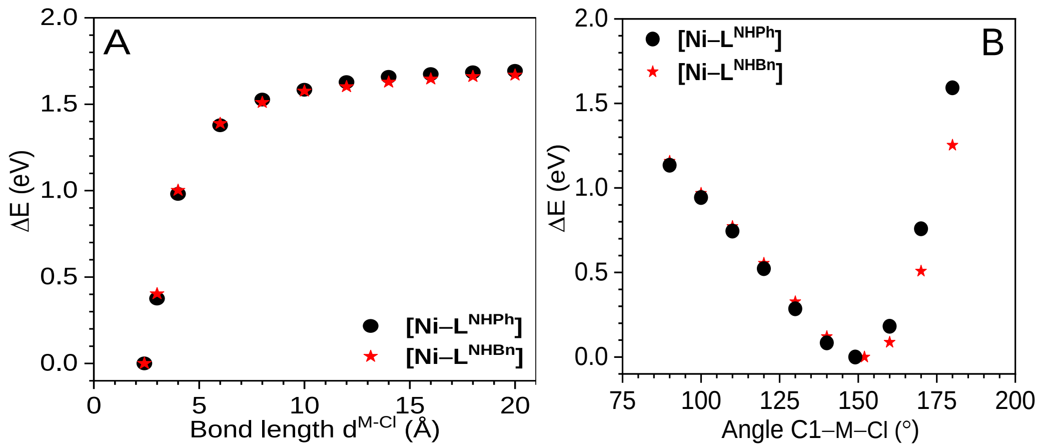

Figure S23. DFT-calculated energies as a function of the Ni–Cl bond length (A) and the C1–Ni–Cl angle (B) in [Ni(N^C^N)Cl], N^C^N = L^{NHPh} and L^{NHBn}, calculated in THF.

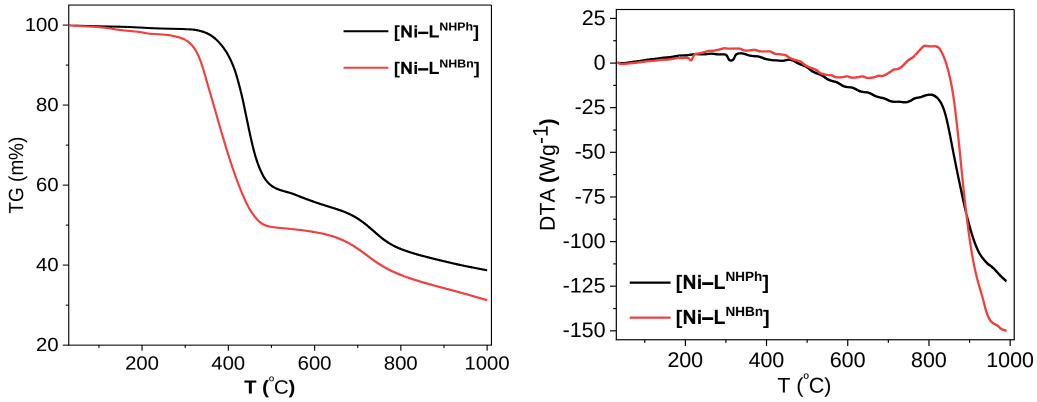

Figure S24. TG (left) and DTA (right) of [Ni(N^C^N)Cl] (N^C^N = L^{NHPh} and L^{NHBn}) under argon atmosphere.

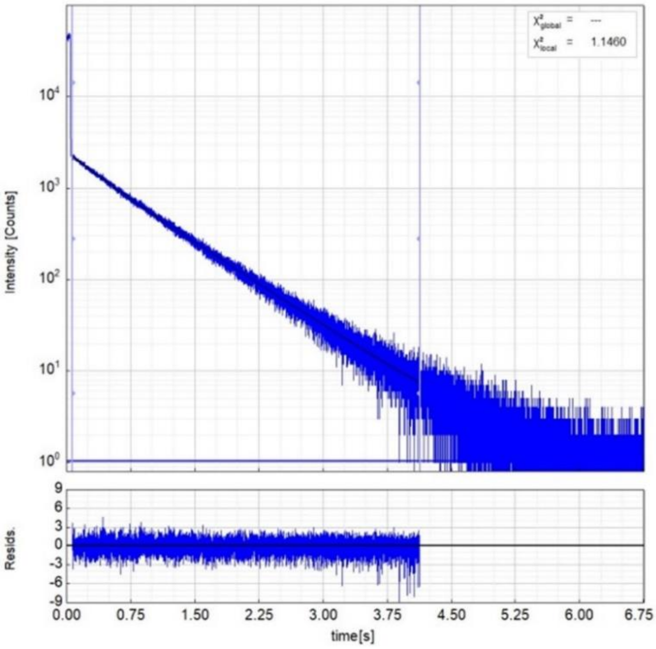

Parameter	Value	Δ	δ
A_1[kCnts/Chnl]	1.856	±0.084	4.5%
τ_1[ns]	718 000 000	±11 000 000	1.5%
I_1[kCnts]	5 080	±160	3.1%
A_{Rel1}[%]	82.2	±3.5	4.2%
I_{Rel1}[%]	91.1	±2.9	3.2%
A_2[kCnts/Chnl]	0.404	±0.077	19%
τ_2[ns]	326 000 000	±40 000 000	12%
I_2[kCnts]	510	±170	32%
A_{Rel2}[%]	17.9	±3.5	19%
I_{Rel2}[%]	9.0	±2.9	32%
$Bkgr_{Dec}$[kCnts]	0.0011	±0.0005	40%
τ_{AvInt}[ns]	682 500 000	±2 500 000	0.4%
τ_{AvAmp}[ns]	647 700 000	±2 700 000	0.4%

Figure S25. Left: Raw (experimental) time-resolved phosphorescence decay of [Ni(L^{NHPh})Cl] in a frozen glassy matrix of 2MeTHF at 77 K, including the residuals (λ_{ex} = 376 nm, λ_{em} = 525 nm, c = 10^{-5} M). **Right**: Fitting parameters including pre-exponential factors and confidence limits.

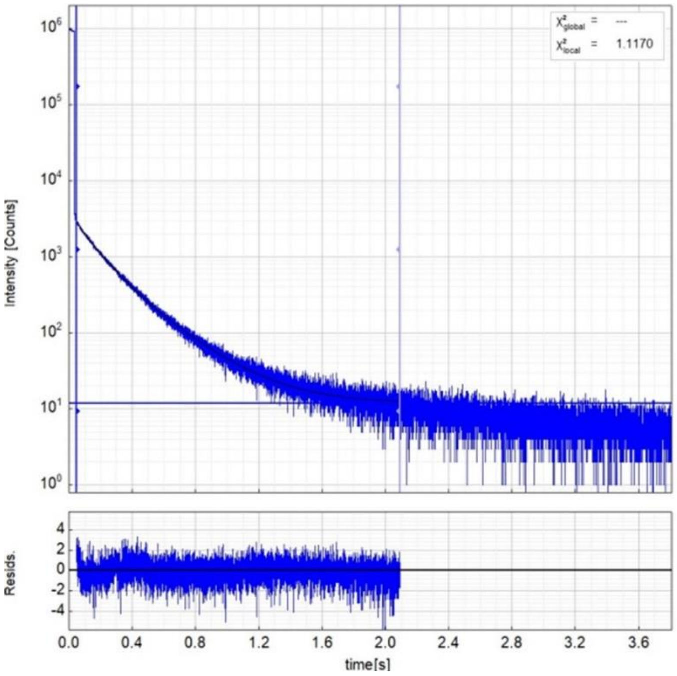

Parameter	Value	Δ	δ
A_1[kCnts/Chnl]	0.990	±0.082	8.2%
τ_1[ns]	281 000 000	±7 700 000	2.7%
I_1[kCnts]	1 061	±60	5.6%
A_{Rel1}[%]	35.0	±2.8	7.9%
I_{Rel1}[%]	55.4	±3.2	5.7%
A_2[kCnts/Chnl]	1.841	±0.072	3.9%
τ_2[ns]	122 000 000	±4 000 000	3.2%
I_2[kCnts]	857	±62	7.2%
A_{Rel2}[%]	65.1	±2.8	4.3%
I_{Rel2}[%]	44.7	±3.2	7.1%
$Bkgr_{Dec}$[kCnts]	0.0120	±0.0004	2.6%
τ_{AvInt}[ns]	210 000 000	±1 100 000	0.5%
τ_{AvAmp}[ns]	177 560 000	±930 000	0.5%

Figure S26. Left: Raw (experimental) time-resolved phosphorescence decay of [Ni(L^{NHBn})Cl] in a frozen glassy matrix of 2MeTHF at 77 K, including the residuals (λ_{ex} = 376 nm, λ_{em} = 525 nm, c = 10^{-5} M). **Right**: Fitting parameters including pre-exponential factors and confidence limits.

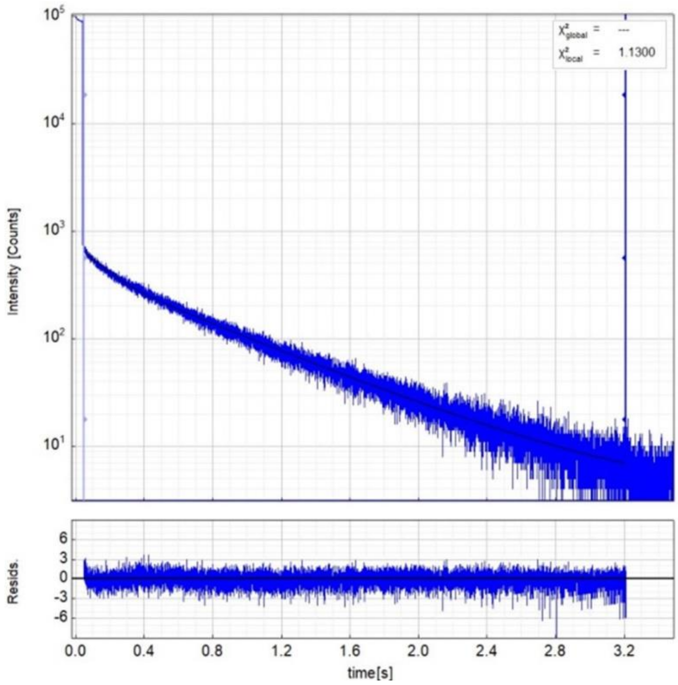

Parameter	Value	Δ	δ
A_1[kCnts/Chnl]	0.4051	±0.0076	1.9%
τ_1[ns]	676 500 000	±9 000 000	1.3%
I_1[kCnts]	1 045.4	±7.3	0.7%
A_{Rel1}[%]	62.7	±1.0	1.5%
I_{Rel1}[%]	89.0	±0.9	0.9%
A_2[kCnts/Chnl]	0.2420	±0.0060	2.5%
τ_2[ns]	140 100 000	±9 200 000	6.6%
I_2[kCnts]	129.4	±10.0	7.7%
A_{Rel2}[%]	37.4	±1.0	2.5%
I_{Rel2}[%]	11.1	±0.9	7.4%
$Bkgr_{Dec}$[kCnts]	0.0031	±0.0004	11%
τ_{AvInt}[ns]	617 500 000	±5 100 000	0.8%
τ_{AvAmp}[ns]	476 000 000	±5 500 000	1.1%

Figure S27. Left: Raw (experimental) time-resolved phosphorescence decay of [Ni(L^{NHPh})Cl] within a PMMA film at 6 K, including the residuals (λ_{ex} = 376 nm, λ_{em} = 525 nm). **Right**: Fitting parameters including pre-exponential factors and confidence limits.

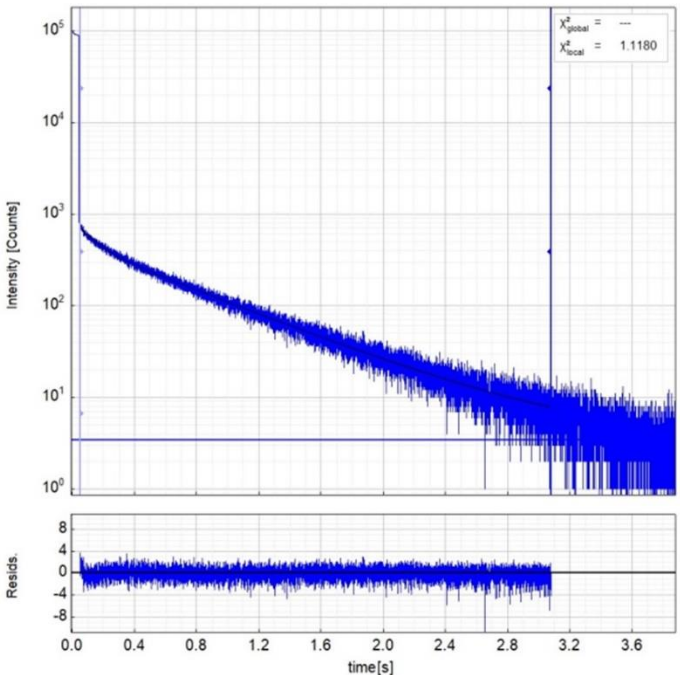

Parameter	Value	Δ	δ
A_1[kCnts/Chnl]	0.4545	±0.0063	1.4%
τ_1[ns]	652 600 000	±6 800 000	1.0%
I_1[kCnts]	1 131.3	±5.8	0.5%
A_{Rel1}[%]	64.8	±0.8	1.1%
I_{Rel1}[%]	91.1	±0.7	0.7%
A_2[kCnts/Chnl]	0.2473	±0.0061	2.5%
τ_2[ns]	117 400 000	±8 200 000	7.0%
I_2[kCnts]	110.7	±7.7	7.0%
A_{Rel2}[%]	35.3	±0.8	2.0%
I_{Rel2}[%]	9.0	±0.7	6.8%
$Bkgr_{Dec}$[kCnts]	0.0035	±0.0004	8.8%
τ_{AvInt}[ns]	604 900 000	±3 900 000	0.6%
τ_{AvAmp}[ns]	464 000 000	±5 600 000	1.2%

Figure S28. Left: Raw (experimental) time-resolved phosphorescence decay of [Ni(L^{NHPh})Cl] within a PMMA film at 100 K, including the residuals (λ_{ex} = 376 nm, λ_{em} = 525 nm). **Right**: Fitting parameters including pre-exponential factors and confidence limits.

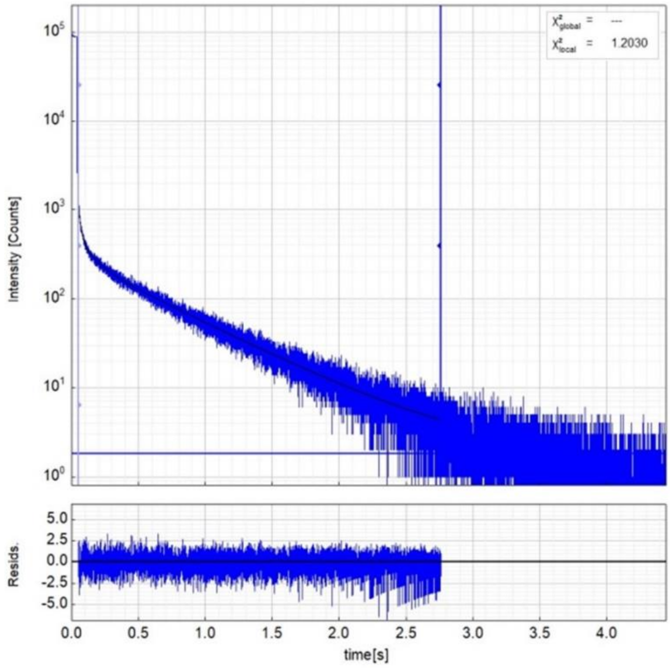

Parameter	Value	Δ	δ
A_1[kCnts/Chnl]	0.2662	±0.0088	3.3%
τ_1[ns]	583 000 000	±13 000 000	2.1%
I_1[kCnts]	591.7	±7.8	1.3%
A_{Rel1}[%]	22.9	±0.7	2.8%
I_{Rel1}[%]	82.5	±1.4	1.7%
A_2[kCnts/Chnl]	0.693	±0.027	3.8%
τ_2[ns]	16 700 000	±2 000 000	12%
I_2[kCnts]	44.0	±6.1	14%
A_{Rel2}[%]	59.5	±2.5	4.1%
I_{Rel2}[%]	6.2	±0.9	13%
A_3[kCnts/Chnl]	0.207	±0.030	14%
τ_3[ns]	104 000 000	±21 000 000	20%
I_3[kCnts]	81.8	±5.6	6.8%
A_{Rel3}[%]	17.8	±2.2	12%
I_{Rel3}[%]	11.4	±0.8	6.5%
$Bkgr_{Dec}$[kCnts]	0.0019	±0.0003	16%
τ_{AvInt}[ns]	493 500 000	±5 900 000	1.2%
τ_{AvAmp}[ns]	161 400 000	±5 300 000	3.2%

Figure S29. Left: Raw (experimental) time-resolved phosphorescence decay of [Ni(L^{NHPh})Cl] within a PMMA film at 200 K, including the residuals (λ_{ex} = 376 nm, λ_{em} = 525 nm). **Right**: Fitting parameters including pre-exponential factors and confidence limits.

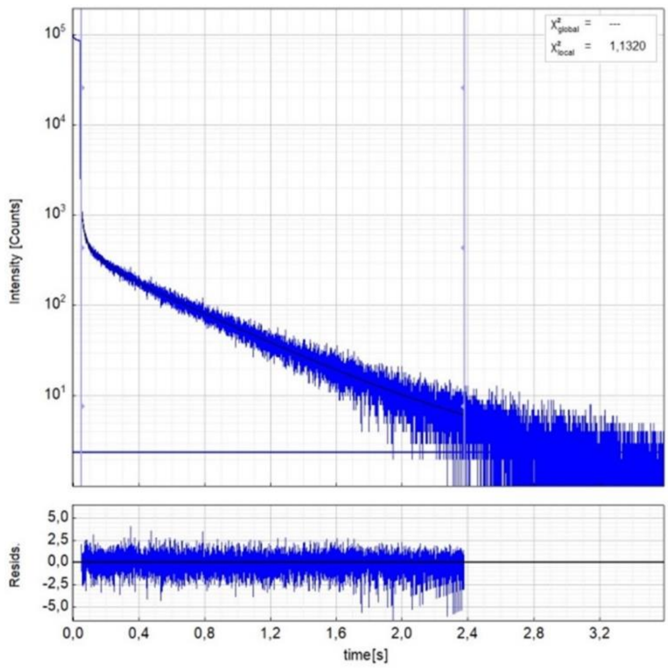

Parameter	Value	Δ	δ
A_1[kCnts/Chnl]	0,3325	±0,0096	2,9%
τ_1[ns]	523 000 000	±11 000 000	2,0%
I_1[kCnts]	662,2	±7,1	1,1%
A_{Rel1}[%]	26,8	±0,8	2,8%
I_{Rel1}[%]	85,8	±1,2	1,4%
A_2[kCnts/Chnl]	0,198	±0,017	8,5%
τ_2[ns]	101 000 000	±16 000 000	16%
I_2[kCnts]	76,3	±7,7	10,0%
A_{Rel2}[%]	16,0	±1,2	7,2%
I_{Rel2}[%]	9,9	±1,0	9,4%
A_3[kCnts/Chnl]	0,713	±0,026	3,5%
τ_3[ns]	12 500 000	±1 300 000	9,9%
I_3[kCnts]	33,8	±3,3	9,7%
A_{Rel3}[%]	57,4	±1,6	2,6%
I_{Rel3}[%]	4,4	±0,5	9,4%
$Bkgr_{Dec}$[kCnts]	0,0024	±0,0004	16%
τ_{AvInt}[ns]	458 300 000	±5 200 000	1,1%
τ_{AvAmp}[ns]	162 900 000	±4 500 000	2,7%

Figure S30. Left: Raw (experimental) time-resolved phosphorescence decay of [Ni(L^{NHPh})Cl] within a PMMA film at 250 K, including the residuals (λ_{ex} = 376 nm, λ_{em} = 525 nm). **Right**: Fitting parameters including pre-exponential factors and confidence limits.

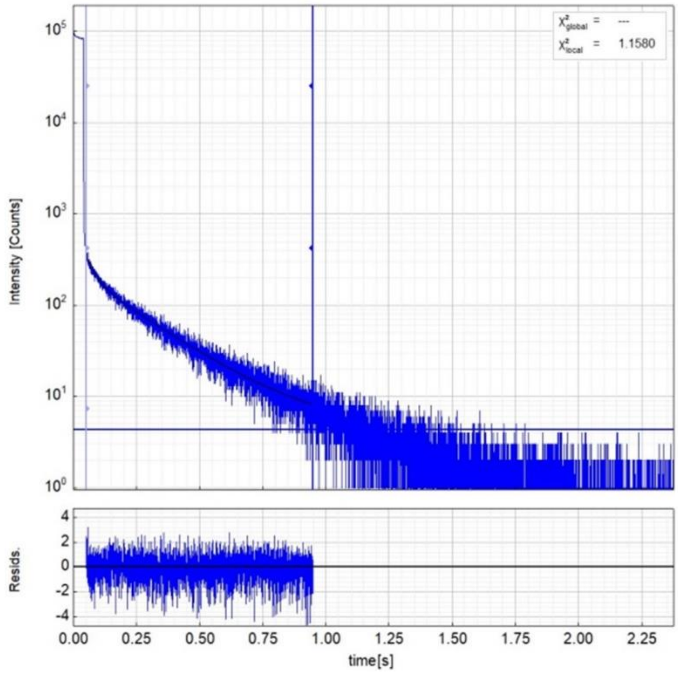

Parameter	Value	Δ	δ
A_1[kCnts/Chnl]	0.1894	±0.0069	3.6%
τ_1[ns]	230 200 000	±8 000 000	3.4%
I_1[kCnts]	166.3	±1.8	1.1%
A_{Rel1}[%]	55.5	±1.7	3.0%
I_{Rel1}[%]	89.1	±1.3	1.4%
A_2[kCnts/Chnl]	0.1520	±0.0071	4.7%
τ_2[ns]	35 300 000	±4 400 000	12%
I_2[kCnts]	20.5	±2.7	13%
A_{Rel2}[%]	44.6	±1.7	3.7%
I_{Rel2}[%]	11.0	±1.3	12%
$Bkgr_{Dec}$[kCnts]	0.0044	±0.0007	16%
τ_{AvInt}[ns]	208 900 000	±5 400 000	2.6%
τ_{AvAmp}[ns]	143 400 000	±4 700 000	3.2%

Figure S31. Left: Raw (experimental) time-resolved phosphorescence decay of [Ni(L^{NHPh})Cl] within a PMMA film at 300 K, including the residuals (λ_{ex} = 376 nm, λ_{em} = 525 nm). **Right**: Fitting parameters including pre-exponential factors and confidence limits.

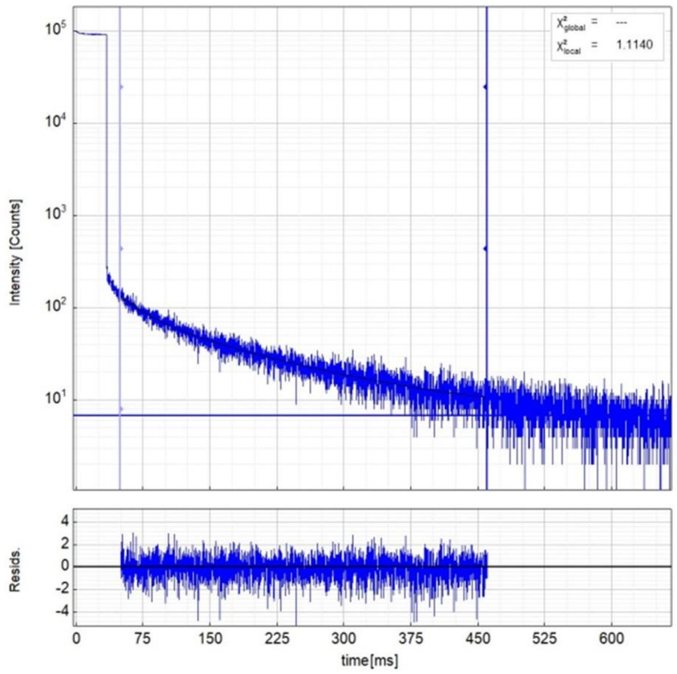

Parameter	Value	Δ	δ
A_1[kCnts/Chnl]	0.0716	±0.0036	4.9%
τ_1[ns]	140 500 000	±8 500 000	6.0%
I_1[kCnts]	76.8	±2.1	2.6%
A_{Rel1}[%]	57.0	±2.5	4.4%
I_{Rel1}[%]	85.8	±2.2	2.5%
A_2[kCnts/Chnl]	0.0542	±0.0030	5.4%
τ_2[ns]	30 800 000	±3 600 000	12%
I_2[kCnts]	12.8	±2.2	17%
A_{Rel2}[%]	43.1	±2.5	5.8%
I_{Rel2}[%]	14.3	±2.2	15%
$Bkgr_{Dec}$[kCnts]	0.0069	±0.0008	11%
τ_{AvInt}[ns]	124 900 000	±6 500 000	5.2%
τ_{AvAmp}[ns]	93 300 000	±3 100 000	3.3%

Figure S32. Left: Raw (experimental) time-resolved phosphorescence decay of [Ni(L^{NHBn})Cl] within a PMMA film at 6 K, including the residuals (λ_{ex} = 376 nm, λ_{em} = 525 nm). **Right**: Fitting parameters including pre-exponential factors and confidence limits.

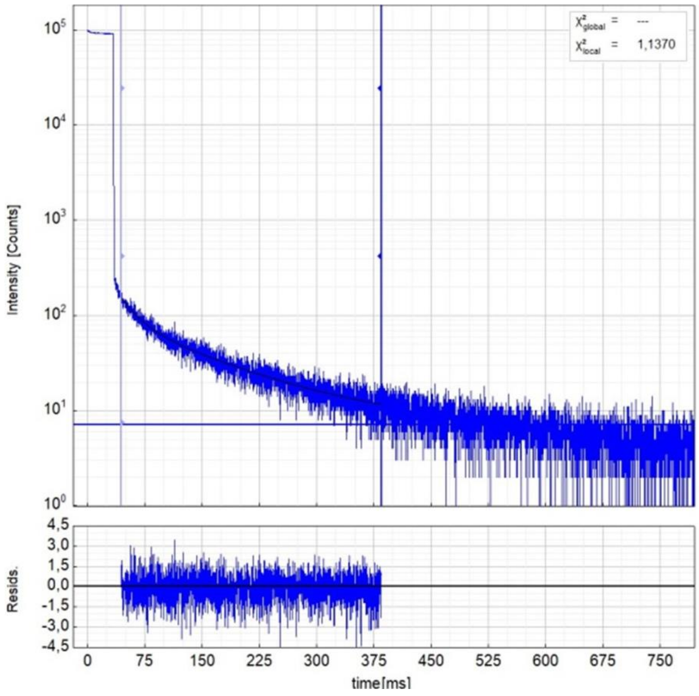

Parameter	Value	Δ	δ
A_1[kCnts/Chnl]	0,0749	±0,0044	5,8%
τ_1[ns]	121 900 000	±8 000 000	6,6%
I_1[kCnts]	69,7	±2,2	3,1%
A_{Rel1}[%]	51,9	±2,6	4,9%
I_{Rel1}[%]	85,1	±1,7	2,0%
A_2[kCnts/Chnl]	0,0696	±0,0043	6,2%
τ_2[ns]	23 200 000	±2 200 000	9,2%
I_2[kCnts]	12,3	±1,6	13%
A_{Rel2}[%]	48,2	±2,6	5,3%
I_{Rel2}[%]	15,0	±1,7	11%
$Bkgr_{Dec}$[kCnts]	0,0071	±0,0011	14%
τ_{AvInt}[ns]	107 100 000	±6 600 000	6,1%
τ_{AvAmp}[ns]	74 400 000	±3 400 000	4,6%

Figure S33. Left: Raw (experimental) time-resolved phosphorescence decay of [Ni(L^{NHBn})Cl] within a PMMA film at 100 K, including the residuals (λ_{ex} = 376 nm, λ_{em} = 525 nm). **Right**: Fitting parameters including pre-exponential factors and confidence limits.

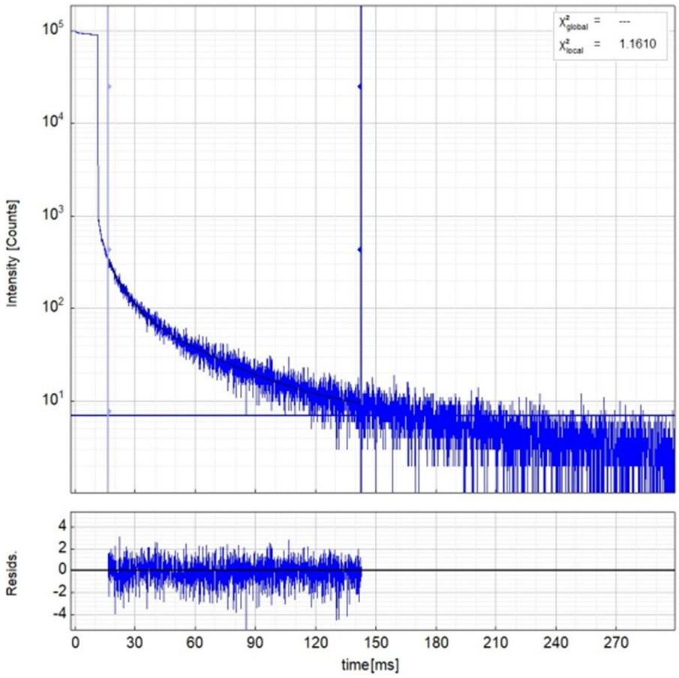

Parameter	Value	Δ	δ
A_1[kCnts/Chnl]	0.110	±0.012	11%
τ_1[ns]	33 800 000	±3 500 000	10%
I_1[kCnts]	56.4	±1.6	2.8%
A_{Rel1}[%]	34.8	±3.4	9.6%
I_{Rel1}[%]	72.0	±3.2	4.3%
A_2[kCnts/Chnl]	0.206	±0.011	5.1%
τ_2[ns]	7 000 000	±750 000	11%
I_2[kCnts]	22.0	±3.1	14%
A_{Rel2}[%]	65.3	±3.4	5.1%
I_{Rel2}[%]	28.1	±3.2	11%
$Bkgr_{Dec}$[kCnts]	0.0070	±0.0012	16%
τ_{AvInt}[ns]	26 300 000	±2 000 000	7.4%
τ_{AvAmp}[ns]	16 300 000	±820 000	5.0%

Figure S34. Left: Raw (experimental), time-resolved phosphorescence decay of [Ni(L^{NHBn})Cl] within a PMMA film at 200 K, including the residuals (λ_{ex} = 376 nm, λ_{em} = 525 nm). **Right**: Fitting parameters including pre-exponential factors and confidence limits.

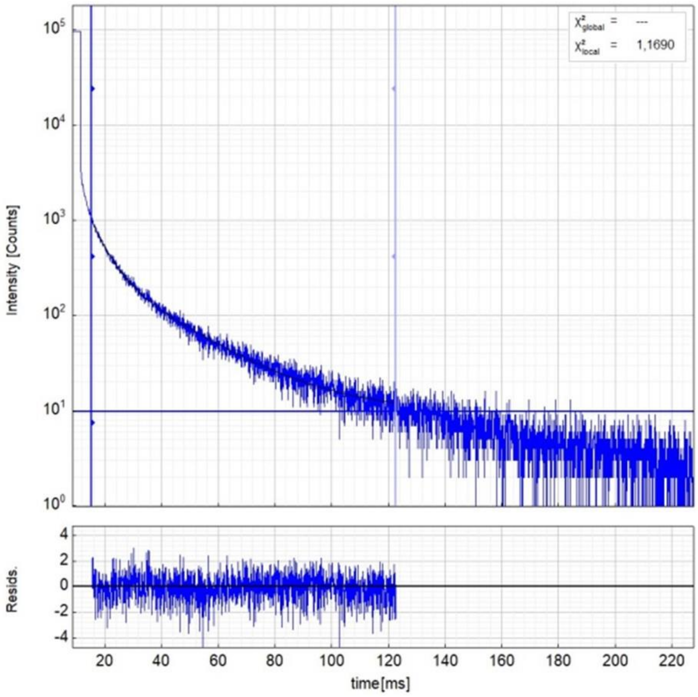

Parameter	Value	Δ	δ
A_1[kCnts/Chnl]	0,299	±0,019	6,2%
τ_1[ns]	22 260 000	±920 000	4,1%
I_1[kCnts]	101,5	±2,5	2,4%
A_{Rel1}[%]	30,8	±1,7	5,3%
I_{Rel1}[%]	66,1	±2,0	2,9%
A_2[kCnts/Chnl]	0,672	±0,016	2,3%
τ_2[ns]	5 100 000	±250 000	4,9%
I_2[kCnts]	52,3	±3,3	6,1%
A_{Rel2}[%]	69,3	±1,7	2,4%
I_{Rel2}[%]	34,0	±2,0	5,6%
$Bkgr_{Dec}$[kCnts]	0,0098	±0,0008	7,2%
τ_{AvInt}[ns]	16 430 000	±400 000	2,4%
τ_{AvAmp}[ns]	10 380 000	±200 000	1,9%

Figure S35. Left: Raw (experimental) time-resolved phosphorescence decay of [Ni(L^{NHBn})Cl] within a PMMA film at 250 K, including the residuals (λ_{ex} = 376 nm, λ_{em} = 525 nm). **Right**: Fitting parameters including pre-exponential factors and confidence limits.

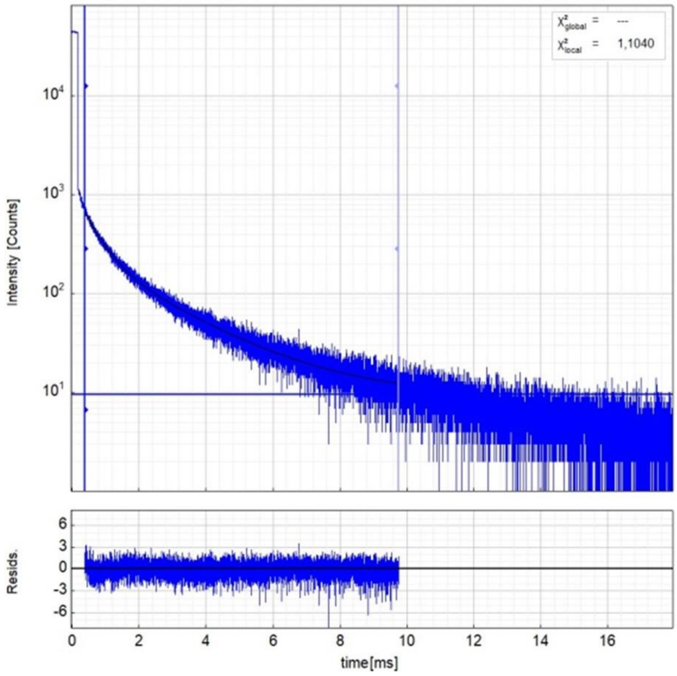

Parameter	Value	Δ	δ
A_1[kCnts/Chnl]	0,4426	±0,0059	1,3%
τ_1[ns]	498 000	±14 000	2,7%
I_1[kCnts]	215,2	±7,7	3,6%
A_{Rel1}[%]	66,7	±1,0	1,4%
I_{Rel1}[%]	31,6	±1,0	3,1%
A_2[kCnts/Chnl]	0,2212	±0,0072	3,2%
τ_2[ns]	2 160 000	±49 000	2,3%
I_2[kCnts]	466,4	±4,9	1,0%
A_{Rel2}[%]	33,4	±1,0	2,9%
I_{Rel2}[%]	68,5	±1,0	1,4%
$Bkgr_{Dec}$[kCnts]	0,0096	±0,0004	3,9%
τ_{AvInt}[ns]	1 635 000	±23 000	1,4%
τ_{AvAmp}[ns]	1 052 000	±11 000	1,0%

Figure S36. Left: Raw (experimental) time-resolved phosphorescence decay of [Ni(L^{NHBn})Cl] within a PMMA film at 300 K, Including the residuals (λ_{ex} = 376 nm, λ_{em} = 525 nm). **Right**: Fitting parameters including pre-exponential factors and confidence limits.

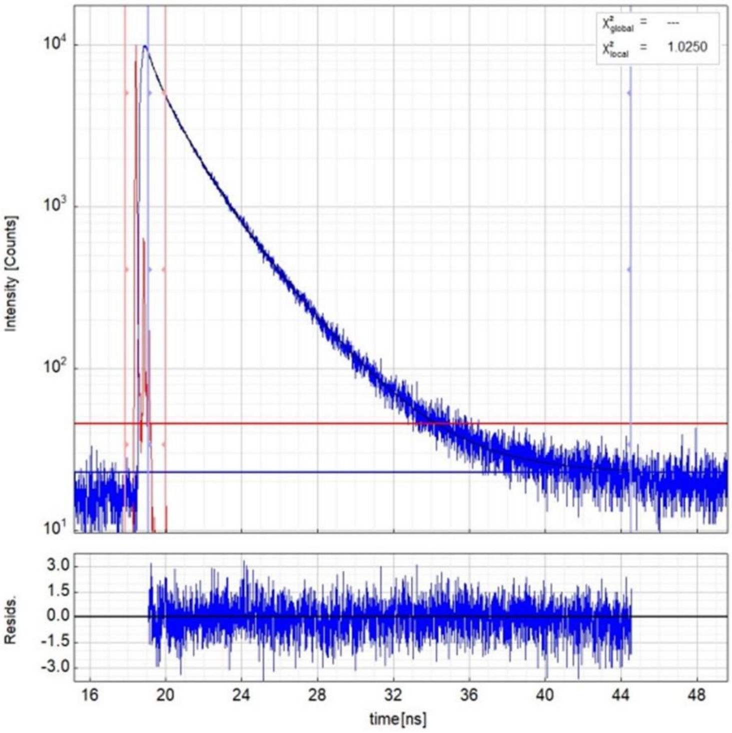

Parameter	Value	Δ	δ
A_1[kCnts/Chnl]	4.82	±0.27	5.5%
τ_1[ns]	3.024	±0.062	2.0%
I_1[kCnts]	1 818	±66	3.6%
A_{Rel1}[%]	36.1	±1.9	5.1%
I_{Rel1}[%]	57.1	±2.7	4.7%
A_2[kCnts/Chnl]	8.52	±0.27	3.1%
τ_2[ns]	1.287	±0.067	5.2%
I_2[kCnts]	1 370	±120	8.0%
A_{Rel2}[%]	64.0	±1.9	2.9%
I_{Rel2}[%]	43.0	±2.7	6.2%
$Bkgr_{Dec}$[kCnts]	0.0227	±0.0005	2.1%
$Bkgr_{IRF}$[Cnts/Chnl]	45.7	±8.2	18%
$Shift_{IRF}$[ps]	- 268	±12	4.4%
A_{Scatt}[kCnts]	205	±70	34%
τ_{AvInt}[ns]	2.278	±0.017	0.7%
τ_{AvAmp}[ns]	1.914	±0.035	1.8%

Figure S37. Left: Raw (experimental) fluorescence decay of [Ni(L^{NHPh})Cl] within a PMMA film at 6 K (blue) and the instrumental response function (IRF, red) Including the residuals (λ_{ex} = 376 nm, λ_{em} = 525 nm). **Right**: Fitting parameters including pre-exponential factors and confidence limits.

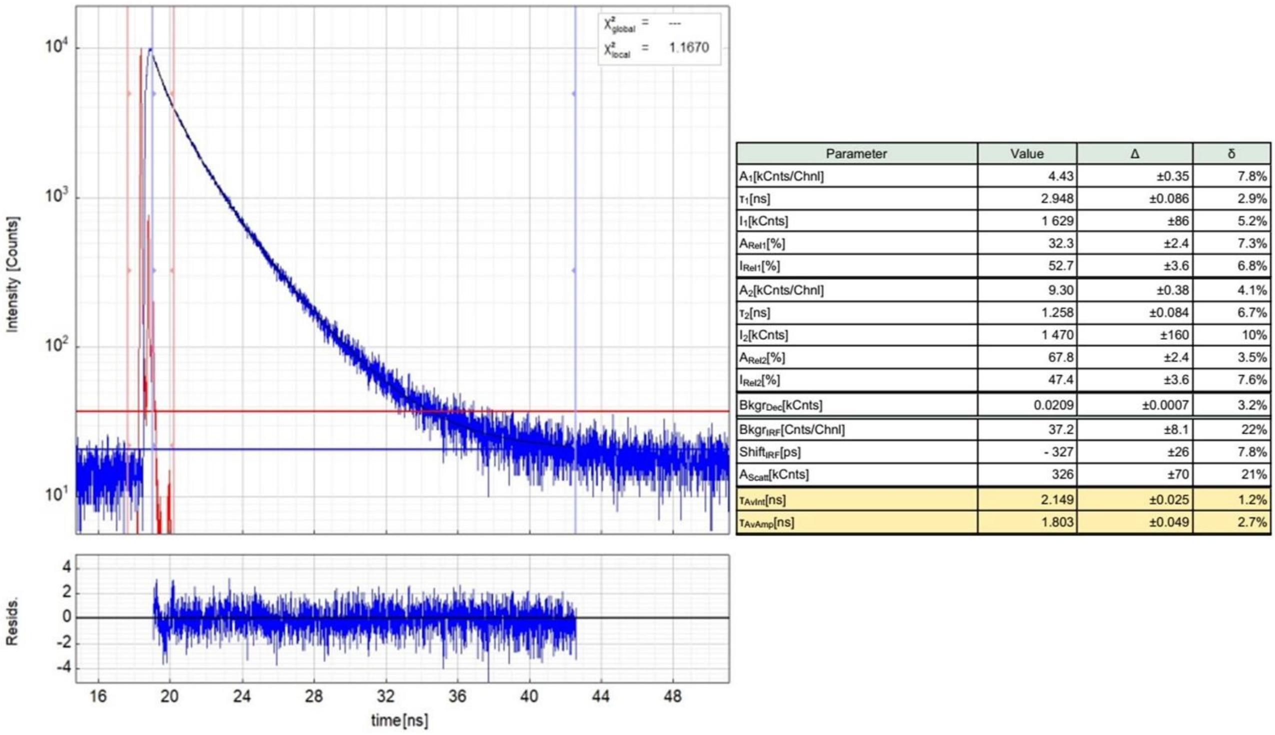

Parameter	Value	Δ	δ
A_1[kCnts/Chnl]	4.43	±0.35	7.8%
τ_1[ns]	2.948	±0.086	2.9%
I_1[kCnts]	1 629	±86	5.2%
A_{Rel1}[%]	32.3	±2.4	7.3%
I_{Rel1}[%]	52.7	±3.6	6.8%
A_2[kCnts/Chnl]	9.30	±0.38	4.1%
τ_2[ns]	1.258	±0.084	6.7%
I_2[kCnts]	1 470	±160	10%
A_{Rel2}[%]	67.8	±2.4	3.5%
I_{Rel2}[%]	47.4	±3.6	7.6%
$Bkgr_{Dec}$[kCnts]	0.0209	±0.0007	3.2%
$Bkgr_{IRF}$[Cnts/Chnl]	37.2	±8.1	22%
$Shift_{IRF}$[ps]	- 327	±26	7.8%
A_{Scatt}[kCnts]	326	±70	21%
τ_{AvInt}[ns]	2.149	±0.025	1.2%
τ_{AvAmp}[ns]	1.803	±0.049	2.7%

Figure S38. Left: Raw (experimental) fluorescence decay of [Ni(L^{NHPh})Cl] within a PMMA film at 100 K (blue) and the instrumental response function (IRF, red) Including the residuals (λ_{ex} = 376 nm, λ_{em} = 525 nm). **Right**: Fitting parameters including pre-exponential factors and confidence limits.

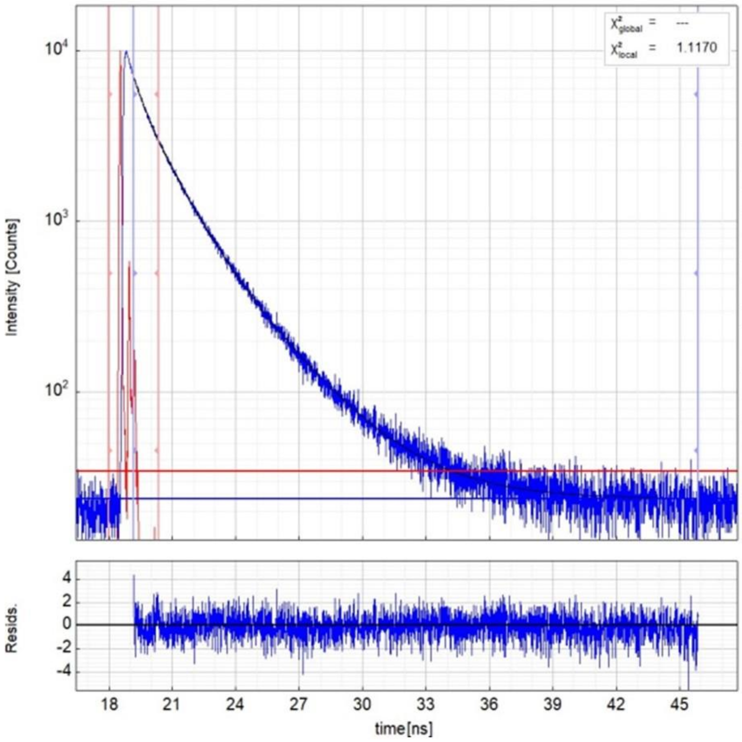

Parameter	Value	Δ	δ
A_1[kCnts/Chnl]	2.90	±0.31	11%
τ_1[ns]	2.86	±0.11	3.6%
I_1[kCnts]	1 035	±73	7.0%
A_{Rel1}[%]	27.3	±2.9	10%
I_{Rel1}[%]	46.2	±4.3	9.2%
A_2[kCnts/Chnl]	7.73	±0.34	4.4%
τ_2[ns]	1.250	±0.079	6.3%
I_2[kCnts]	1 210	±130	10%
A_{Rel2}[%]	72.8	±2.9	3.9%
I_{Rel2}[%]	53.9	±4.3	7.9%
$Bkgr_{Dec}$[kCnts]	0.0237	±0.0005	1.8%
$Bkgr_{IRF}$[Cnts/Chnl]	34.3	±8.2	24%
$Shift_{IRF}$[ps]	- 159	±24	15%
A_{Scatt}[kCnts]	286	±60	21%
τ_{AvInt}[ns]	1.993	±0.024	1.2%
τ_{AvAmp}[ns]	1.689	±0.043	2.5%

Figure S39. Left: Raw (experimental) fluorescence decay of [Ni(L^{NHPh})Cl] within a PMMA film at 200 K (blue) and the instrumental response function (IRF, red) Including the residuals (λ_{ex} = 376 nm, λ_{em} = 525 nm). **Right**: Fitting parameters including pre-exponential factors and confidence limits.

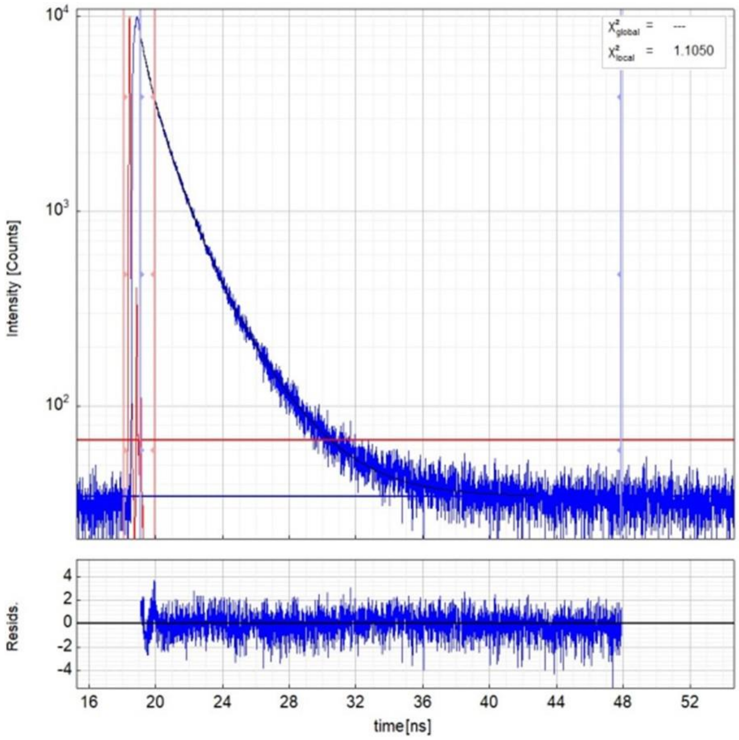

Parameter	Value	Δ	δ
A_1[kCnts/Chnl]	10.76	±0.29	2.6%
τ_1[ns]	1.172	±0.054	4.6%
I_1[kCnts]	1 580	±110	6.6%
A_{Rel1}[%]	79.7	±1.8	2.2%
I_{Rel1}[%]	62.1	±3.0	4.8%
A_2[kCnts/Chnl]	2.75	±0.25	9.0%
τ_2[ns]	2.815	±0.088	3.1%
I_2[kCnts]	966	±60	6.2%
A_{Rel2}[%]	20.4	±1.8	8.8%
I_{Rel2}[%]	38.0	±3.0	7.8%
$Bkgr_{Dec}$[kCnts]	0.0345	±0.0005	1.2%
$Bkgr_{IRF}$[Cnts/Chnl]	67.1	±8.6	13%
$Shift_{IRF}$[ps]	- 262	±12	4.6%
A_{Scatt}[kCnts]	168	±69	41%
τ_{AvInt}[ns]	1.796	±0.019	1.0%
τ_{AvAmp}[ns]	1.506	±0.033	2.1%

Figure S40. Left: Raw (experimental) fluorescence decay of [Ni(L^{NHPh})Cl] within a PMMA film at 250 K (blue) and the instrumental response function (IRF, red) Including the residuals (λ_{ex} = 376 nm, λ_{em} = 525 nm). **Right**: Fitting parameters including pre-exponential factors and confidence limits.

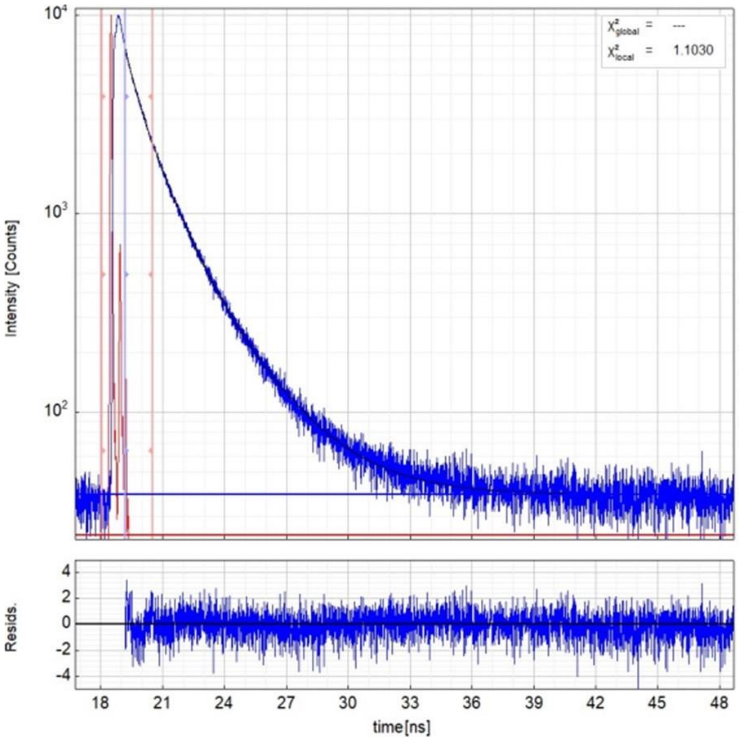

Parameter	Value	Δ	δ
A_1[kCnts/Chnl]	2.23	±0.34	15%
τ_1[ns]	2.65	±0.14	5.0%
I_1[kCnts]	736	±76	10%
A_{Rel1}[%]	20.3	±2.9	14%
I_{Rel1}[%]	38.0	±4.5	12%
A_2[kCnts/Chnl]	8.75	±0.24	2.7%
τ_2[ns]	1.101	±0.073	6.6%
I_2[kCnts]	1 210	±110	8.9%
A_{Rel2}[%]	79.8	±2.9	3.5%
I_{Rel2}[%]	62.1	±4.5	7.2%
$Bkgr_{Dec}$[kCnts]	0.0389	±0.0005	1.1%
$Bkgr_{IRF}$[Cnts/Chnl]	24.3	±6.9	28%
$Shift_{IRF}$[ps]	- 181	±12	6.5%
A_{Scatt}[kCnts]	432	±68	16%
τ_{AvInt}[ns]	1.686	±0.027	1.6%
τ_{AvAmp}[ns]	1.414	±0.043	3.0%

Figure S41. Left: Raw (experimental) fluorescence decay of [Ni(L^{NHPh})Cl] within a PMMA film at 300 K (blue) and the instrumental response function (IRF, red) Including the residuals (λ_{ex} = 376 nm, λ_{em} = 525 nm). **Right**: Fitting parameters including pre-exponential factors and confidence limits.

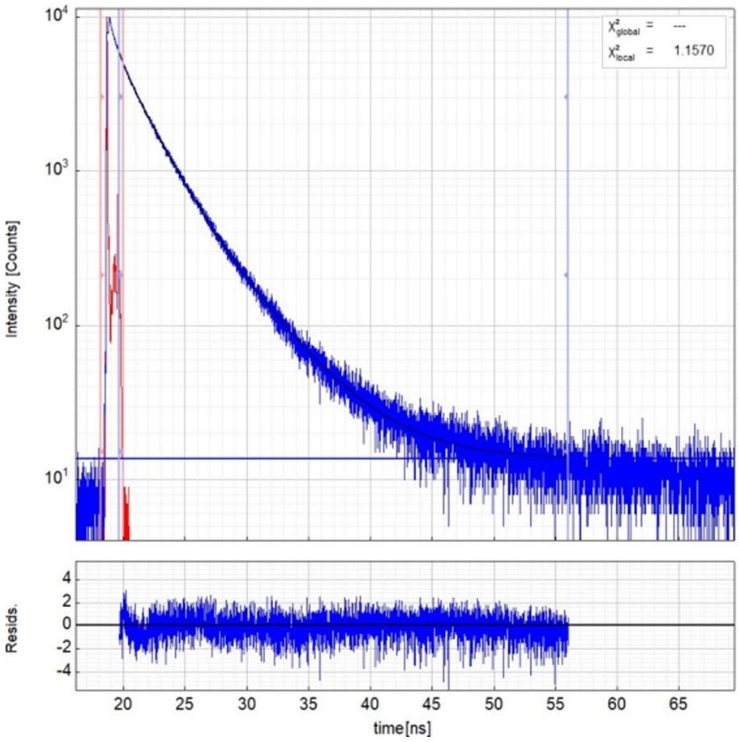

Parameter	Value	Δ	δ
A_1[kCnts/Chnl]	1.88	±0.25	13%
τ_1[ns]	4.29	±0.13	2.8%
I_1[kCnts]	1 010	±110	11%
A_{Rel1}[%]	29.9	±3.0	9.8%
I_{Rel1}[%]	47.0	±3.7	7.7%
A_2[kCnts/Chnl]	4.42	±0.30	6.8%
τ_2[ns]	2.067	±0.070	3.4%
I_2[kCnts]	1 140	±96	8.4%
A_{Rel2}[%]	70.2	±3.0	4.2%
I_{Rel2}[%]	53.1	±3.7	6.8%
$Bkgr_{Dec}$[kCnts]	0.0137	±0.0004	2.2%
$Bkgr_{IRF}$[Cnts/Chnl]	-95	±34	36%
$Shift_{IRF}$[ps]	-21.1	±10.0	47%
A_{Scatt}[kCnts]	240	±30	12%
τ_{AvInt}[ns]	3.110	±0.014	0.4%
τ_{AvAmp}[ns]	2.731	±0.022	0.8%

Figure S42. Left: Raw (experimental) fluorescence decay of [Ni(L^{NHBn})Cl] within a PMMA film at 6 K (blue) and the instrumental response function (IRF, red) Including the residuals (λ_{ex} = 376 nm, λ_{em} = 525 nm). **Right**: Fitting parameters including pre-exponential factors and confidence limits.

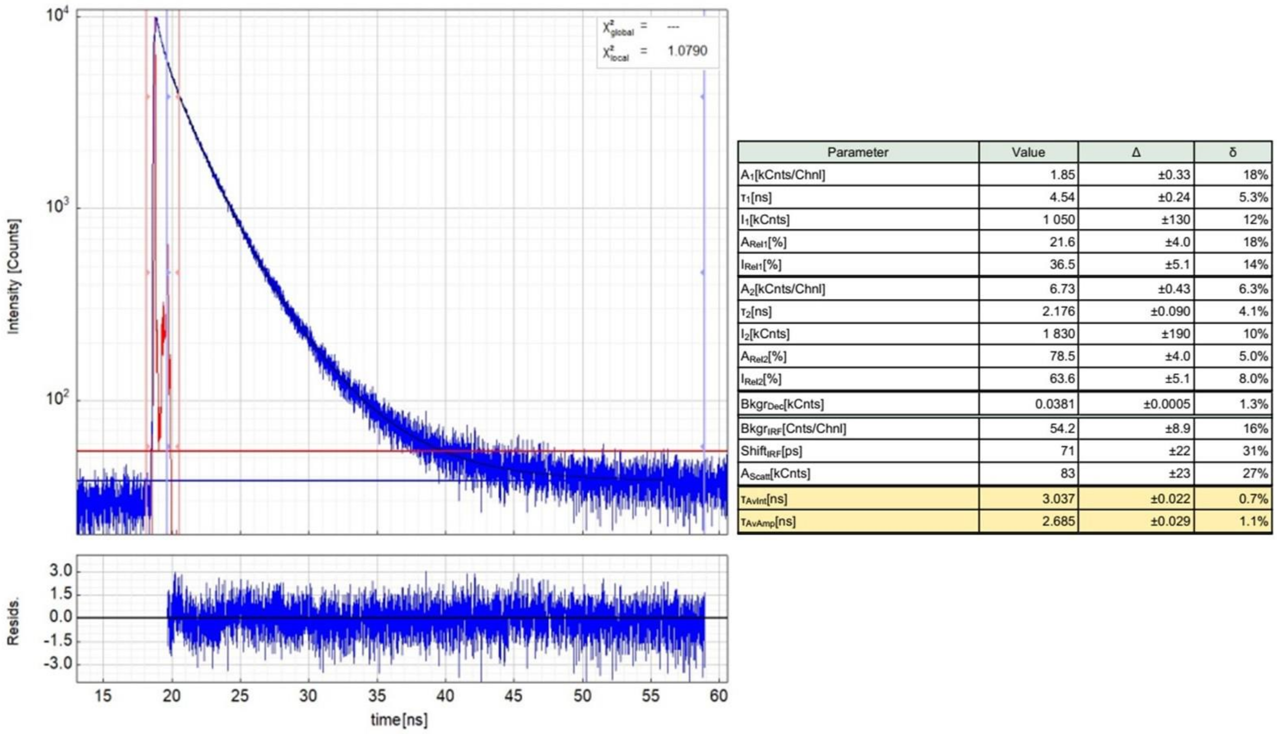

Parameter	Value	Δ	δ
A_1[kCnts/Chnl]	1.85	±0.33	18%
τ_1[ns]	4.54	±0.24	5.3%
I_1[kCnts]	1 050	±130	12%
A_{Rel1}[%]	21.6	±4.0	18%
I_{Rel1}[%]	36.5	±5.1	14%
A_2[kCnts/Chnl]	6.73	±0.43	6.3%
τ_2[ns]	2.176	±0.090	4.1%
I_2[kCnts]	1 830	±190	10%
A_{Rel2}[%]	78.5	±4.0	5.0%
I_{Rel2}[%]	63.6	±5.1	8.0%
$Bkgr_{Dec}$[kCnts]	0.0381	±0.0005	1.3%
$Bkgr_{IRF}$[Cnts/Chnl]	54.2	±8.9	16%
$Shift_{IRF}$[ps]	71	±22	31%
A_{Scatt}[kCnts]	83	±23	27%
τ_{AvInt}[ns]	3.037	±0.022	0.7%
τ_{AvAmp}[ns]	2.685	±0.029	1.1%

Figure S43. Left: Raw (experimental) fluorescence decay of [Ni(L^{NHBn})Cl] within a PMMA film at 100 K (blue) and the instrumental response function (IRF, red) Including the residuals (λ_{ex} = 376 nm, λ_{em} = 525 nm). **Right**: Fitting parameters including pre-exponential factors and confidence limits.

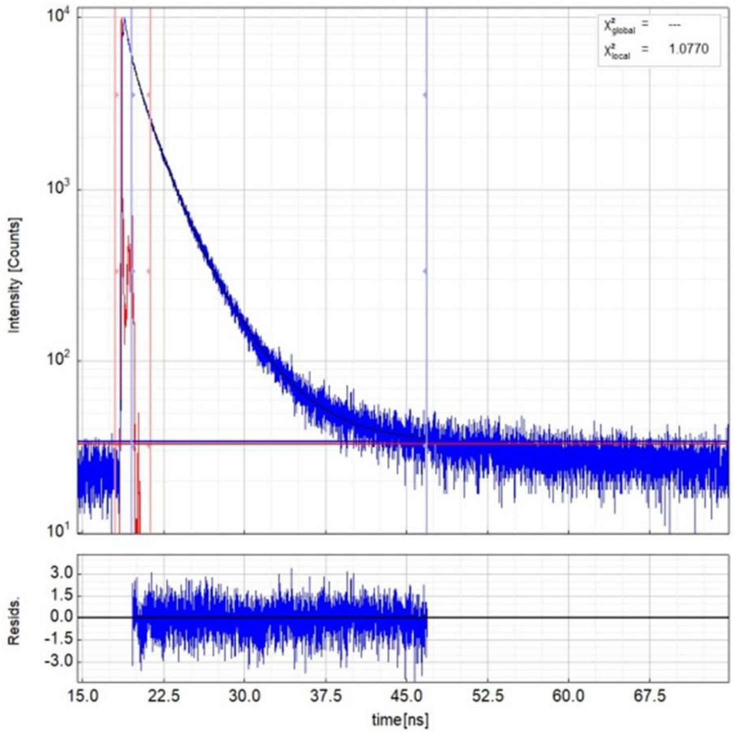

Parameter	Value	Δ	δ
A_1[kCnts/Chnl]	1.86	±0.41	22%
τ_1[ns]	4.12	±0.28	6.6%
I_1[kCnts]	960	±150	15%
A_{Rel1}[%]	23.7	±5.2	22%
I_{Rel1}[%]	39.6	±6.8	17%
A_2[kCnts/Chnl]	6.01	±0.40	6.5%
τ_2[ns]	1.96	±0.13	6.3%
I_2[kCnts]	1 470	±190	13%
A_{Rel2}[%]	76.4	±5.2	6.7%
I_{Rel2}[%]	60.5	±6.8	11%
$Bkgr_{Dec}$[kCnts]	0.0340	±0.0010	2.9%
$Bkgr_{IRF}$[Cnts/Chnl]	32.8	±6.4	19%
$Shift_{IRF}$[ps]	-90	±14	16%
A_{Scatt}[kCnts]	149	±38	25%
τ_{AvInt}[ns]	2.806	±0.037	1.3%
τ_{AvAmp}[ns]	2.462	±0.048	1.9%

Figure S44. Left: Raw (experimental) fluorescence decay of [Ni(L^{NHBn})Cl] within a PMMA film at 200 K (blue) and the instrumental response function (IRF, red) Including the residuals (λ_{ex} = 376 nm, λ_{em} = 525 nm). **Right**: Fitting parameters including pre-exponential factors and confidence limits.

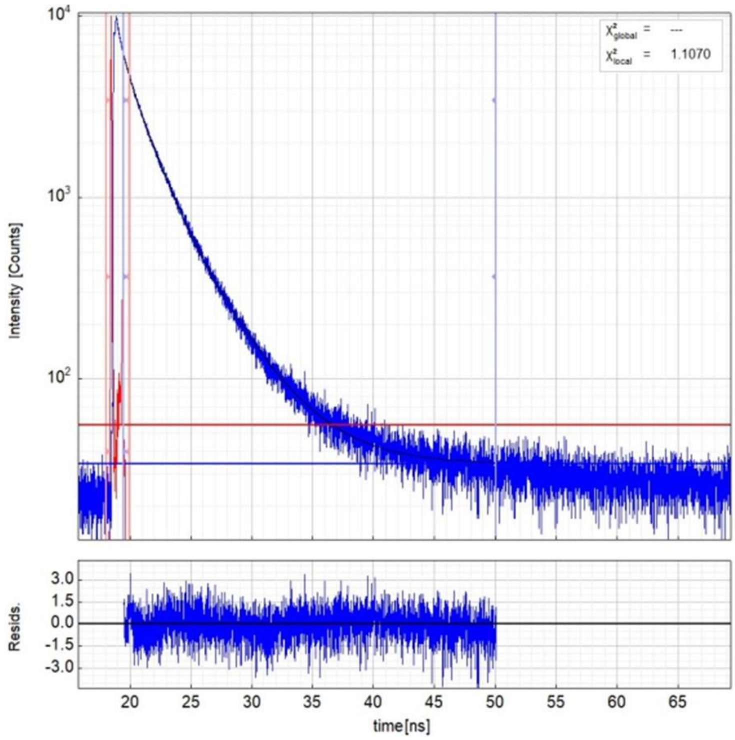

Parameter	Value	Δ	δ
A_1[kCnts/Chnl]	3.01	±0.31	10%
τ_1[ns]	3.80	±0.13	3.3%
I_1[kCnts]	1 425	±99	6.9%
A_{Rel1}[%]	27.3	±2.7	9.7%
I_{Rel1}[%]	46.2	±3.5	7.6%
A_2[kCnts/Chnl]	8.02	±0.40	4.9%
τ_2[ns]	1.660	±0.076	4.6%
I_2[kCnts]	1 670	±150	8.5%
A_{Rel2}[%]	72.8	±2.7	3.6%
I_{Rel2}[%]	53.9	±3.5	6.5%
$Bkgr_{Dec}$[kCnts]	0.0341	±0.0007	1.9%
$Bkgr_{IRF}$[Cnts/Chnl]	55.6	±9.1	16%
$Shift_{IRF}$[ps]	- 237	±34	14%
A_{Scatt}[kCnts]	-79	±73	92%
τ_{AvInt}[ns]	2.646	±0.025	0.9%
τ_{AvAmp}[ns]	2.242	±0.037	1.6%

Figure S45. Left: Raw (experimental) fluorescence decay of [Ni(L^{NHBn})Cl] within a PMMA film at 250 K (blue) and the instrumental response function (IRF, red) Including the residuals (λ_{ex} = 376 nm, λ_{em} = 525 nm). **Right**: Fitting parameters including pre-exponential factors and confidence limits.

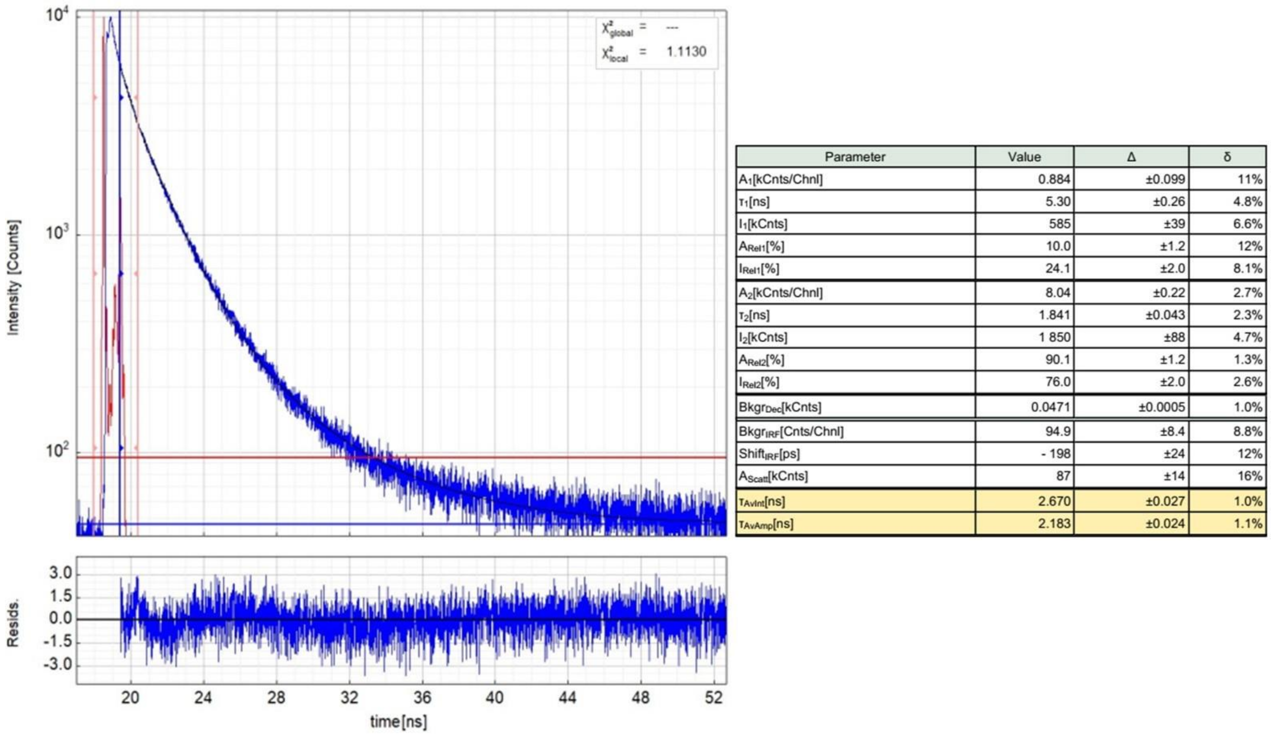

Parameter	Value	Δ	δ
A_1[kCnts/Chnl]	0.884	±0.099	11%
τ_1[ns]	5.30	±0.26	4.8%
I_1[kCnts]	585	±39	6.6%
A_{Rel1}[%]	10.0	±1.2	12%
I_{Rel1}[%]	24.1	±2.0	8.1%
A_2[kCnts/Chnl]	8.04	±0.22	2.7%
τ_2[ns]	1.841	±0.043	2.3%
I_2[kCnts]	1 850	±88	4.7%
A_{Rel2}[%]	90.1	±1.2	1.3%
I_{Rel2}[%]	76.0	±2.0	2.6%
$Bkgr_{Dec}$[kCnts]	0.0471	±0.0005	1.0%
$Bkgr_{IRF}$[Cnts/Chnl]	94.9	±8.4	8.8%
$Shift_{IRF}$[ps]	- 198	±24	12%
A_{Scatt}[kCnts]	87	±14	16%
τ_{AvInt}[ns]	2.670	±0.027	1.0%
τ_{AvAmp}[ns]	2.183	±0.024	1.1%

Figure S46. Left: Raw (experimental) fluorescence decay of [Ni(L^{NHBn})Cl] within a PMMA film at 300 K (blue) and the instrumental response function (IRF, red) Including the residuals (λ_{ex} = 376 nm, λ_{em} = 525 nm). **Right**: Fitting parameters including pre-exponential factors and confidence limits.

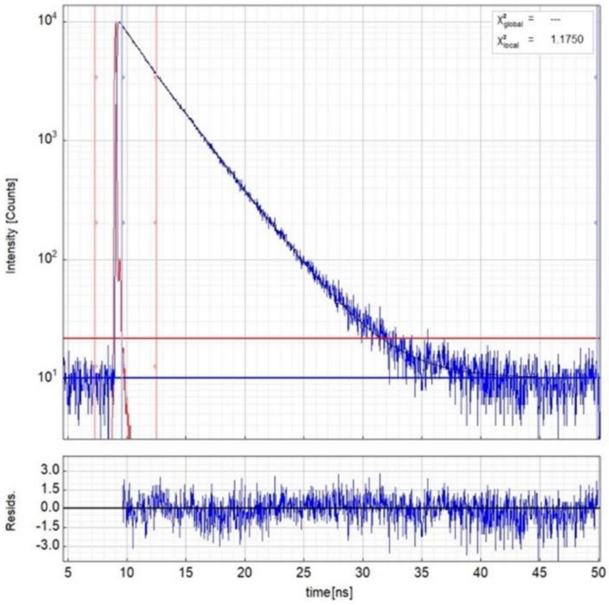

Parameter	Value	Δ	δ
A_1[kCnts/Chnl]	11.45	±0.88	7.6%
τ_1[ns]	3.338	±0.013	0.4%
I_1[kCnts]	1 194	±91	7.6%
A_{Rel1}[%]	100.0	---	---
I_{Rel1}[%]	100.0	---	---
$Bkgr_{Dec}$[kCnts]	0.0101	±0.0004	3.3%
$Bkgr_{IRF}$[Cnts/Chnl]	21.6	±1.5	6.7%
$Shift_{IRF}$[ps]	- 120	±240	185%
τ_{AvInt}[ns]	3.338	±0.013	0.4%
τ_{AvAmp}[ns]	3.338	±0.013	0.4%

Figure S47. Left: Raw (experimental) fluorescence decay of [Ni(L^{NHPh})Cl] within a glassy matrix (2MeTHF, c = 10^{-5} M) at 77 K (blue) and the instrumental response function (IRF, red) Including the residuals (λ_{ex} = 376 nm, λ_{em} = 525 nm). **Right**: Fitting parameters including pre-exponential factors and confidence limits.

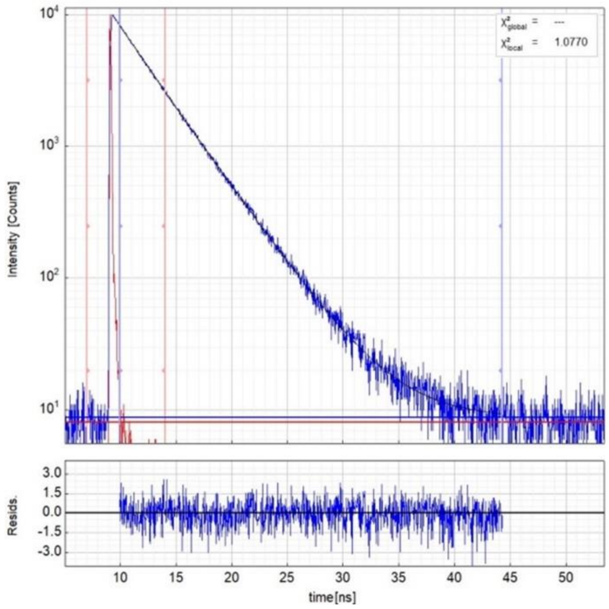

Parameter	Value	Δ	δ
A_1[kCnts/Chnl]	10.69	±0.17	1.5%
τ_1[ns]	3.634	±0.019	0.5%
I_1[kCnts]	1 214	±20	1.6%
A_{Rel1}[%]	100.0	---	---
I_{Rel1}[%]	100.0	---	---
$Bkgr_{Dec}$[kCnts]	0.0088	±0.0005	5.1%
$Bkgr_{IRF}$[Cnts/Chnl]	8.08	±0.92	11%
$Shift_{IRF}$[ps]	33	±50	153%
τ_{AvInt}[ns]	3.634	±0.019	0.5%
τ_{AvAmp}[ns]	3.634	±0.019	0.5%

Figure S48. Left: Raw (experimental) fluorescence photoluminescence decay of [Ni(L^{NHBn})Cl] within a glassy matrix (2MeTHF, c = 10^{-5} M) at 77 K (blue) and the instrumental response function (IRF, red) Including the residuals (λ_{ex} = 376 nm, λ_{em} = 525 nm). **Right**: Fitting parameters including pre-exponential factors and confidence limits.

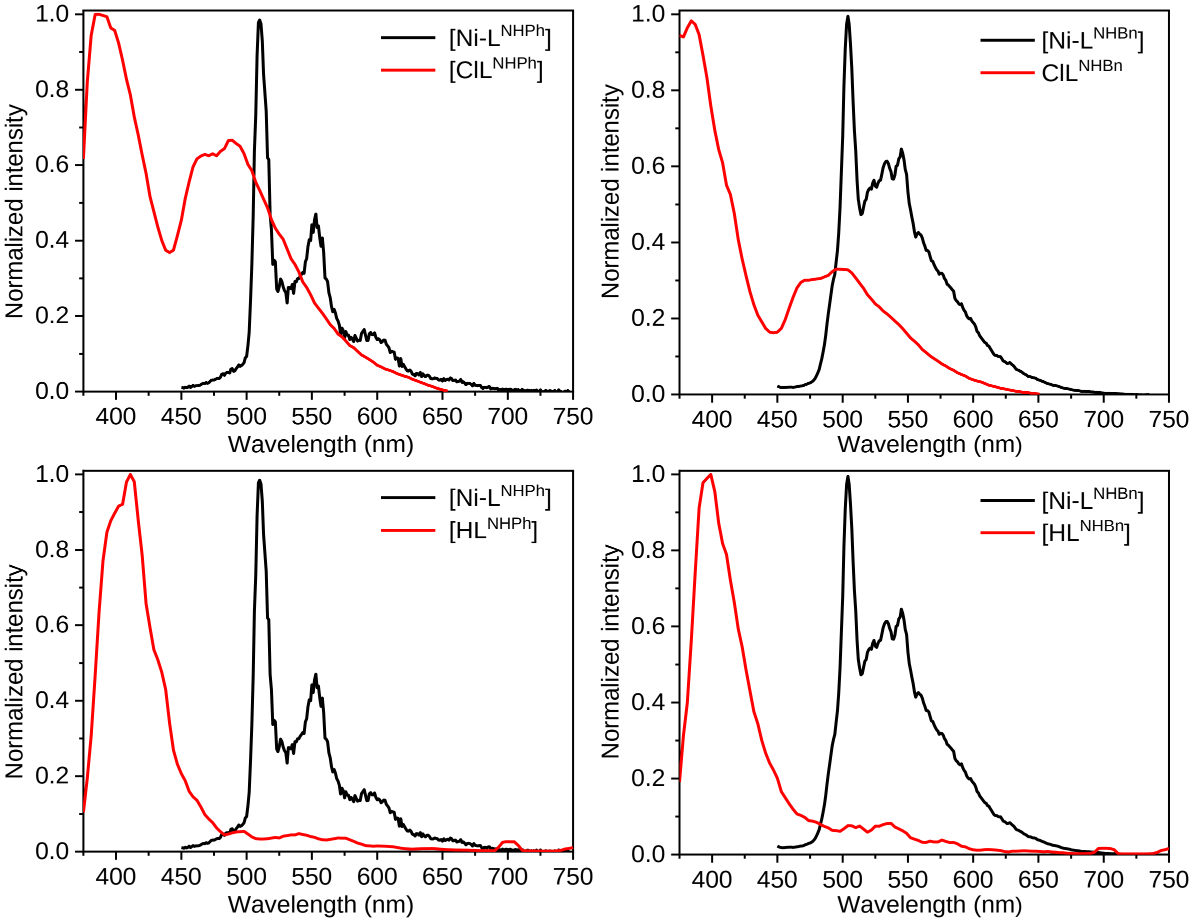

Figure S49. Emission spectra of [Ni(N^C^N)Cl] (N^C^N = L^{NHPh} and L^{NHBn}) complexes (black), their ligand precursors ClL^{NHPh} and ClL^{NHBn}, and the protonated ligands HL^{NHPh} and HL^{NHBn} (all in red) in a frozen glassy matrix (2MeTHF, *c* = 10^{-5} M), λ_{ex} = 350 nm.

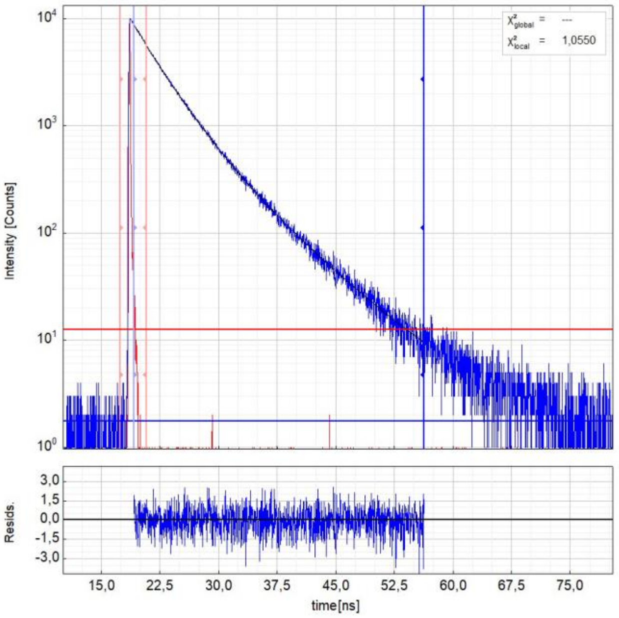

Parameter	Value	Δ	δ
A_1[kCnts/Chnl]	1,897	±0,073	3,8%
τ_1[ns]	6,884	±0,099	1,4%
I_1[kCnts]	409	±11	2,7%
A_{Rel1}[%]	18,3	±0,7	3,8%
I_{Rel1}[%]	31,8	±1,0	3,0%
A_2[kCnts/Chnl]	8,49	±0,12	1,4%
τ_2[ns]	3,308	±0,026	0,8%
I_2[kCnts]	877	±19	2,1%
A_{Rel2}[%]	81,8	±0,7	0,9%
I_{Rel2}[%]	68,3	±1,0	1,4%
$Bkgr_{Dec}$[kCnts]	0,0018	±0,0009	49%
$Bkgr_{IRF}$[Cnts/Chnl]	12,6	±3,4	27%
$Shift_{IRF}$[ps]	83	±24	28%
A_{Scatt}[kCnts]	- 169	±54	31%
τ_{AvInt}[ns]	4,444	±0,011	0,2%
τ_{AvAmp}[ns]	3,961	±0,016	0,4%

Figure S50. Left: Time-resolved fluorescence decay (blue) of **ClL^{NHPh}** and the instrument response function (IRF, red) in a frozen glassy matrix of 2MeTHF at 77 K, including the residuals (λ_{ex} = 376 nm, λ_{obs} = 475 nm). **Right**: Fitting parameters including pre-exponential factors and confidence limits.

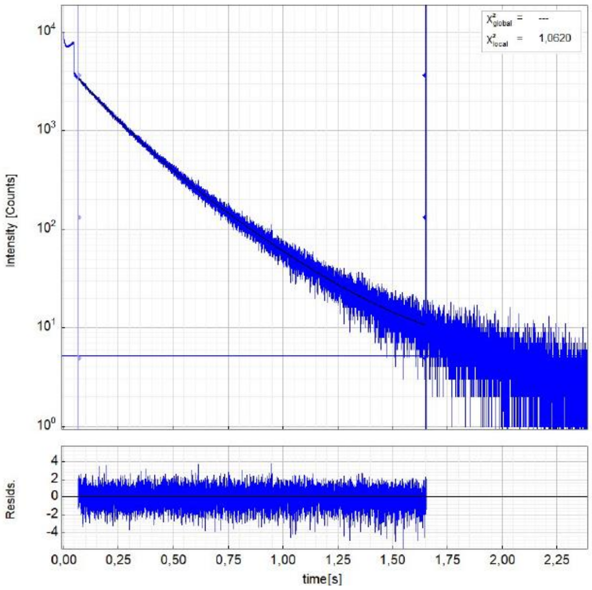

Parameter	Value	Δ	δ
A_1[kCnts/Chnl]	1,310	±0,096	7,3%
τ_1[ns]	289 000 000	±6 200 000	2,1%
I_1[kCnts]	2 890	±150	5,0%
A_{Rel1}[%]	38,8	±2,8	7,1%
I_{Rel1}[%]	56,1	±2,9	5,0%
A_2[kCnts/Chnl]	2,071	±0,090	4,3%
τ_2[ns]	143 500 000	±3 200 000	2,2%
I_2[kCnts]	2 270	±150	6,4%
A_{Rel2}[%]	61,3	±2,8	4,5%
I_{Rel2}[%]	44,0	±2,9	6,4%
$Bkgr_{Dec}$[kCnts]	0,0052	±0,0005	8,2%
τ_{AvInt}[ns]	224 960 000	±880 000	0,4%
τ_{AvAmp}[ns]	199 810 000	±520 000	0,3%

Figure S51. Left: Time-resolved phosphorescence decay (blue) of **ClL^{NHPh}** in a frozen glassy matrix of 2MeTHF at 77 K, including the residuals (λ_{ex} = 376 nm, λ_{obs} = 475 nm). **Right**: Fitting parameters including pre-exponential factors and confidence limits.

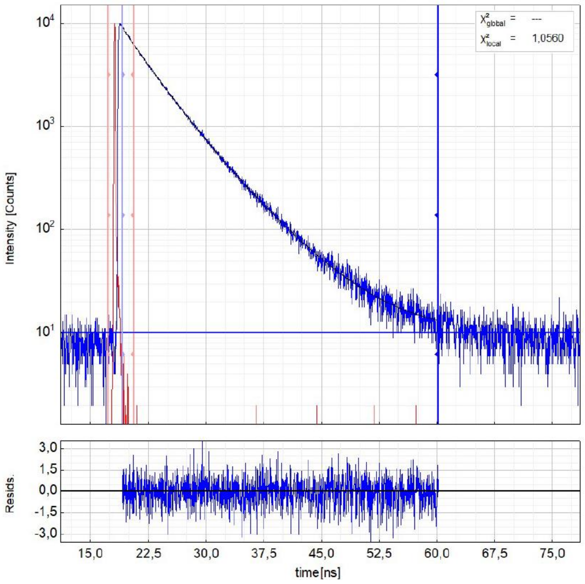

Parameter	Value	Δ	δ
A_1[kCnts/Chnl]	3,4	±1,1	33%
τ_1[ns]	6,04	±0,47	7,6%
I_1[kCnts]	640	±170	26%
A_{Rel1}[%]	29,0	±9,3	32%
I_{Rel1}[%]	42	±12	27%
A_2[kCnts/Chnl]	8,3	±1,1	12%
τ_2[ns]	3,47	±0,22	6,1%
I_2[kCnts]	900	±170	19%
A_{Rel2}[%]	71,1	±9,3	13%
I_{Rel2}[%]	59	±12	19%
$Bkgr_{Dec}$[kCnts]	0,0101	±0,0010	9,7%
$Bkgr_{IRF}$[Cnts/Chnl]	-1,0	±1,7	180%
$Shift_{IRF}$[ps]	- 491	±12	2,3%
A_{Scatt}[kCnts]	2 280	±810	35%
τ_{AvInt}[ns]	4,532	±0,030	0,6%
τ_{AvAmp}[ns]	4,211	±0,050	1,2%

Figure S53. Left: Time-resolved fluorescence decay (blue) of **ClL^{NHBn}** and the instrument response function (IRF, red) in a frozen glassy matrix of 2MeTHF at 77 K, including the residuals (λ_{ex} = 376 nm, λ_{obs} = 475 nm). **Right**: Fitting parameters including pre-exponential factors and confidence limits.

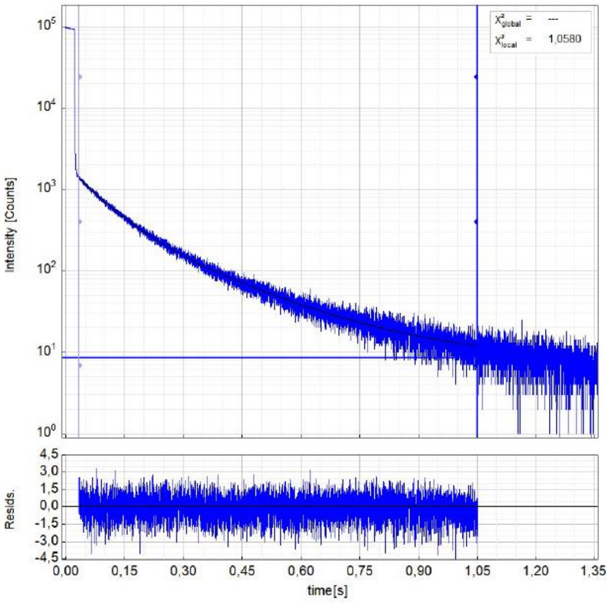

Parameter	Value	Δ	δ
A_1[kCnts/Chnl]	0,410	±0,023	5,5%
τ_1[ns]	214 900 000	±5 600 000	2,6%
I_1[kCnts]	672	±19	2,7%
A_{Rel1}[%]	30,3	±1,7	5,6%
I_{Rel1}[%]	55,5	±1,8	3,2%
A_2[kCnts/Chnl]	0,943	±0,024	2,5%
τ_2[ns]	75 000 000	±1 500 000	1,9%
I_2[kCnts]	540	±24	4,4%
A_{Rel2}[%]	69,8	±1,7	2,4%
I_{Rel2}[%]	44,6	±1,8	4,0%
$Bkgr_{Dec}$[kCnts]	0,0085	±0,0005	5,1%
τ_{AvInt}[ns]	152 600 000	±1 500 000	0,9%
τ_{AvAmp}[ns]	117 320 000	±520 000	0,4%

Figure S54. Left: Time-resolved phosphorescence decay (blue) of **ClL^{NHBn}** in a frozen glassy matrix of 2MeTHF at 77 K, including the residuals (λ_{ex} = 376 nm, λ_{obs} = 475 nm). **Right**: Fitting parameters including pre-exponential factors and confidence limits.

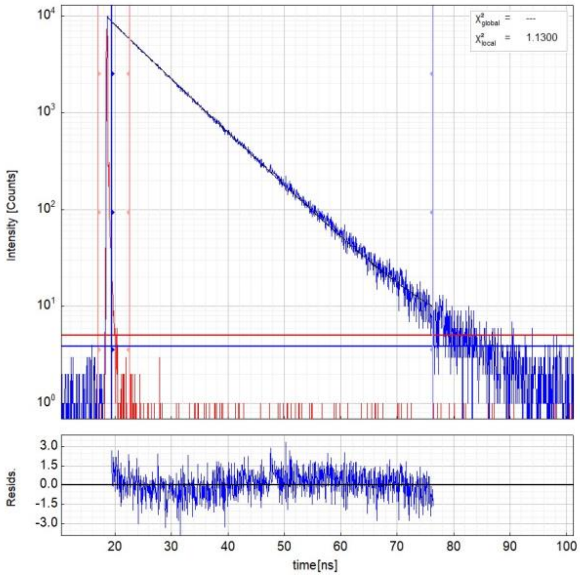

Parameter	Value	Δ	δ
A_1[kCnts/Chnl]	9.807	±0.055	0.6%
τ_1[ns]	7.848	±0.024	0.3%
I_1[kCnts]	1 202.5	±5.5	0.5%
A_{Rel1}[%]	100.0	---	---
I_{Rel1}[%]	100.0	---	---
$Bkgr_{Dec}$[kCnts]	0.0039	±0.0012	30%
$Bkgr_{IRF}$[Cnts/Chnl]	5.0000	---	---
$Shift_{IRF}$[ps]	0.1	±1.9	2830%
A_{Scatt}[kCnts]	- 170	±320	187%
τ_{AvInt}[ns]	7.848	±0.024	0.3%
τ_{AvAmp}[ns]	7.848	±0.024	0.3%

Figure S55. Left: Time-resolved fluorescence decay (blue) of **HL^{NHPh}** and the instrument response function (IRF, red) in a frozen glassy matrix of 2MeTHF at 77 K, including the residuals (λ_{ex} = 376 nm, λ_{obs} = 475 nm). **Right**: Fitting parameters including pre-exponential factors and confidence limits.

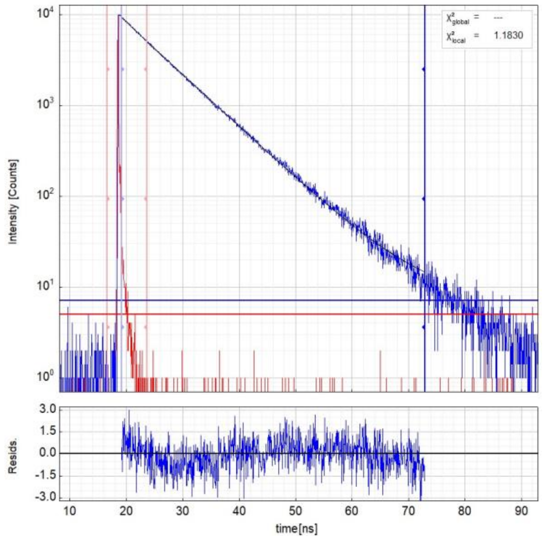

Parameter	Value	Δ	δ
A_1[kCnts/Chnl]	10.240	±0.053	0.5%
τ_1[ns]	7.554	±0.015	0.2%
I_1[kCnts]	1 208.6	±6.7	0.5%
A_{Rel1}[%]	100.0	---	---
I_{Rel1}[%]	100.0	---	---
$Bkgr_{Dec}$[kCnts]	0.0072	±0.0018	25%
$Bkgr_{IRF}$[Cnts/Chnl]	5.0000	---	---
$Shift_{IRF}$[ps]	-1.8	±5.4	313%
A_{Scatt}[kCnts]	150	±120	80%
τ_{AvInt}[ns]	7.554	±0.015	0.2%
τ_{AvAmp}[ns]	7.554	±0.015	0.2%

Figure S56. Left: Time-resolved fluorescence decay (blue) of HL^{NHBn} and the instrument response function (IRF, red) in a frozen glassy matrix of 2MeTHF at 77 K, including the residuals (λ_{ex} = 376 nm, λ_{obs} = 475 nm). **Right**: Fitting parameters including pre-exponential factors and confidence limits.

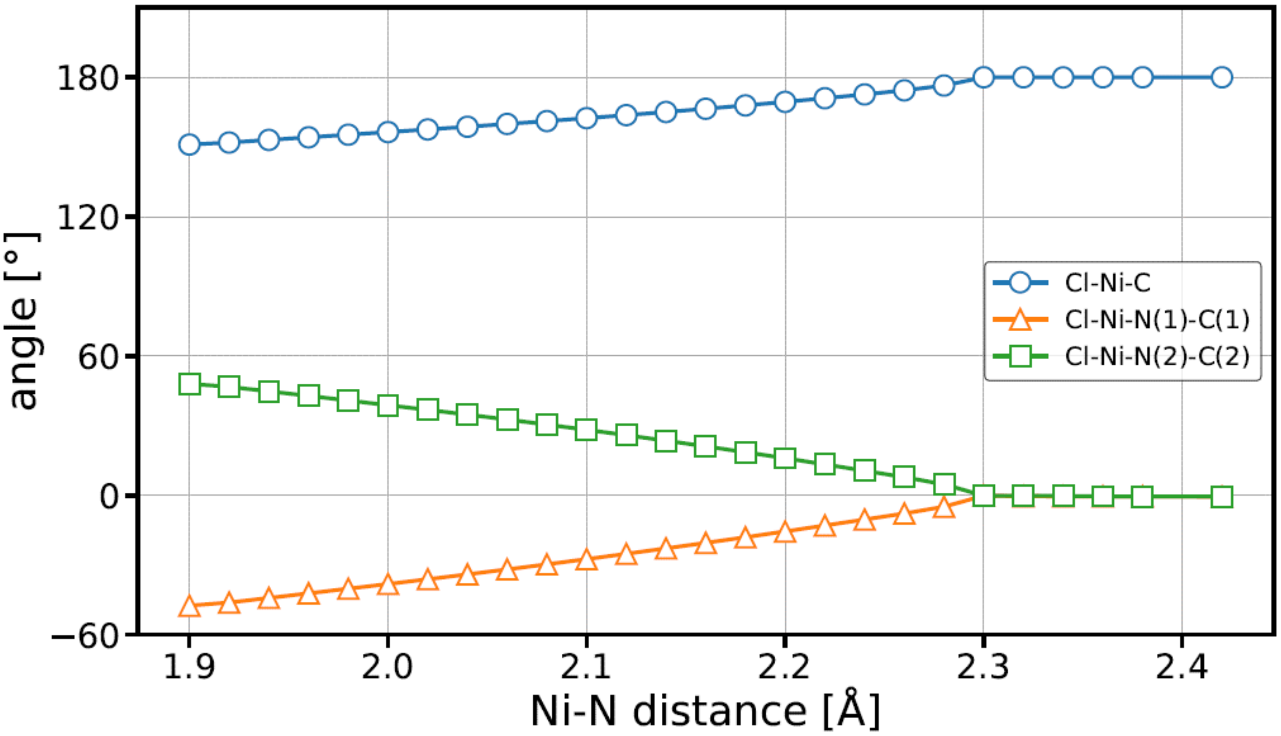

Figure S57. Dihedral angles Cl–Ni–N–C(1) and Cl–Ni–N–C(2), and bond angle Cl–Ni–C for the constraint optimized structures of [Ni(L^{NHBn})Cl] along the Ni–N coordinate (cf. Figure 6 of the main paper).

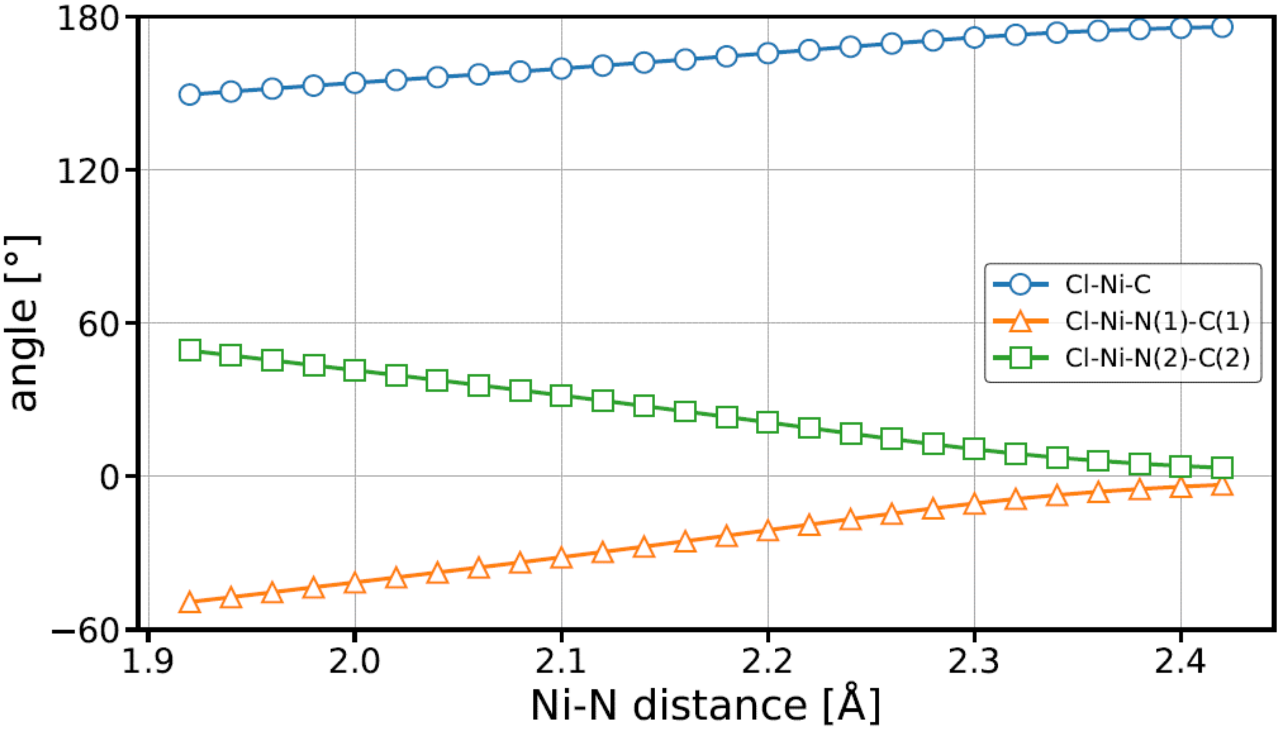

Figure S58. Dihedral angles Cl–Ni–N–C(1) and Cl–Ni–N–C(2), and bond angle Cl–Ni–C for the constraint optimized structures of [Ni(L^{NHPh})Cl] along the Ni–N coordinate (cf. Figure 6 of the main paper).

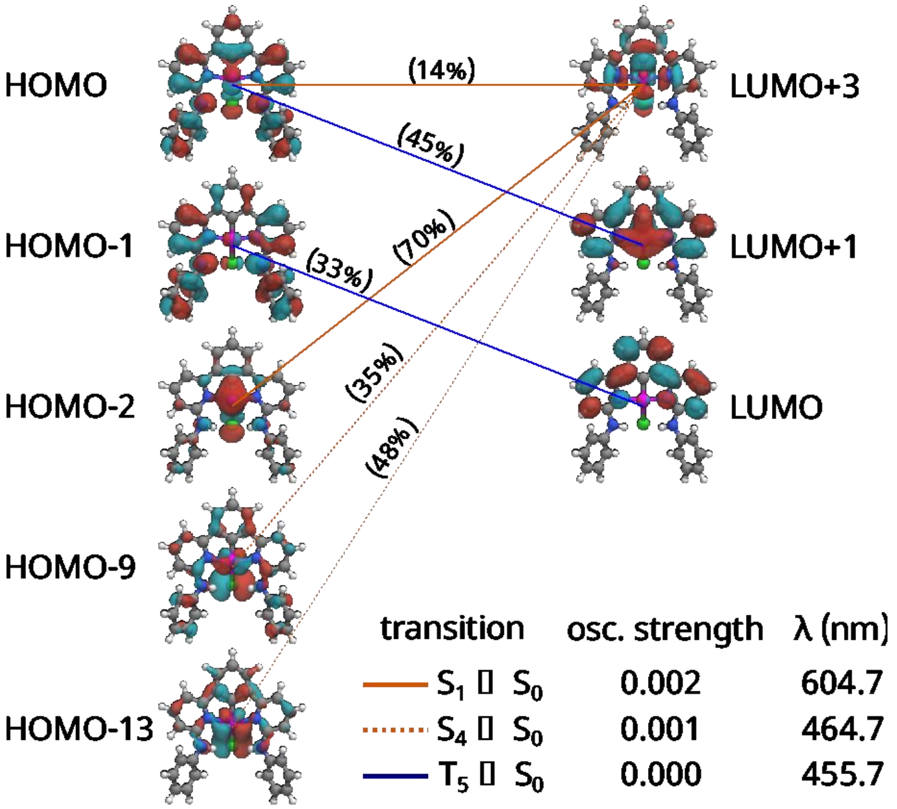

Figure S59. Main contributing orbitals to the $S_1 \rightarrow S_0$, $S_4 \rightarrow S_0$, and $T_5 \rightarrow S_0$ of [Ni(L^{NHPh})Cl] from TDA calculations as detailed in Table S16.

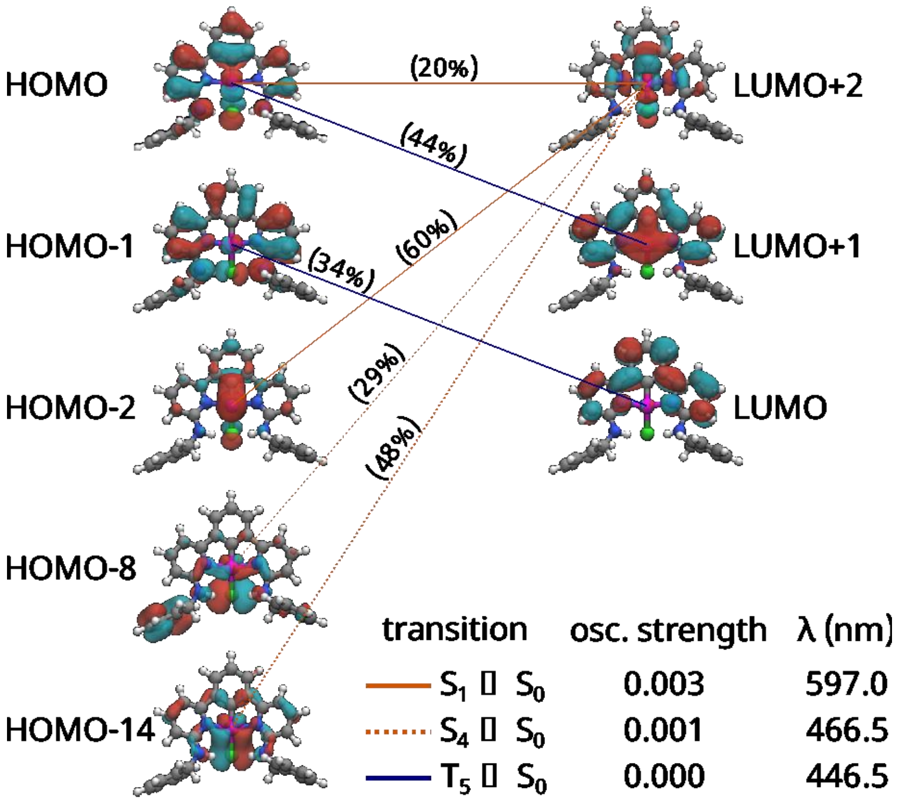

Figure S60. Main contributing orbitals to the $S_1 \rightarrow S_0$, $S_4 \rightarrow S_0$, and $T_5 \rightarrow S_0$ of [Ni(L^{NHBn})Cl] from TDA calculations as detailed in Table S17.

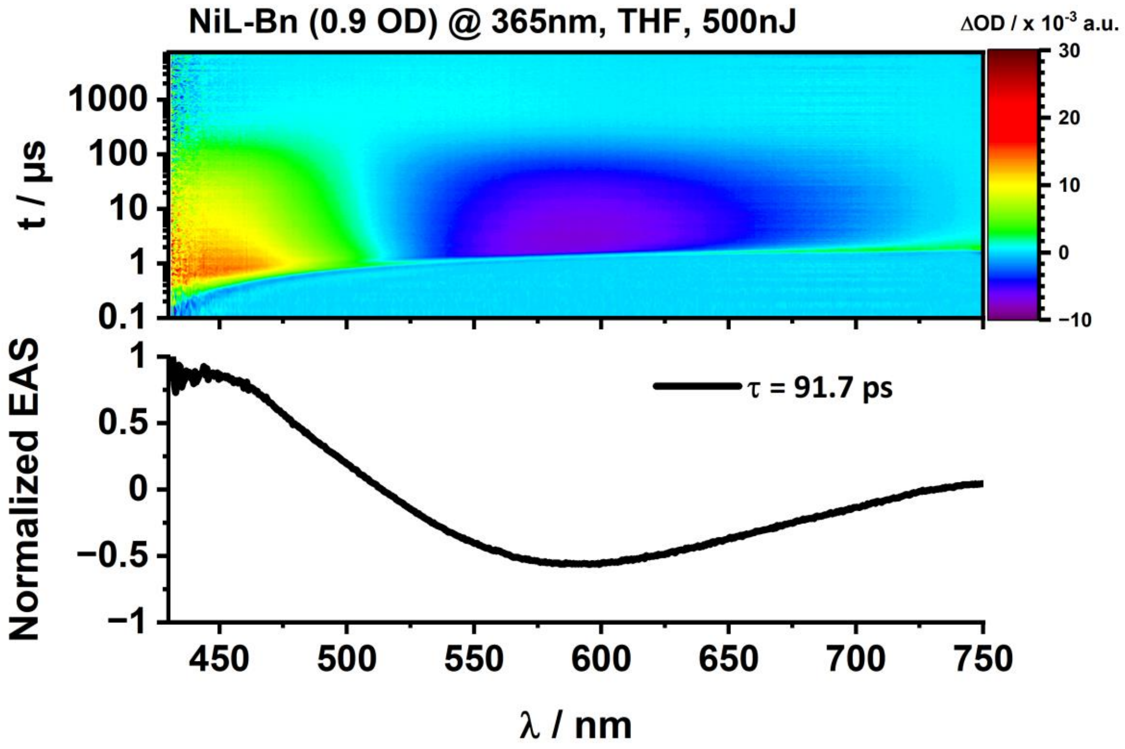

Figure S61. 3D heat map of [Ni(L^{NHBn})Cl] at 298 K obtained by cryostat supported fs-TAS measurements in THF (top). Evolution associated spectrum (bottom) of the fs-TA data of [Ni(L^{NHPh})Cl] with a lifetime of 91.7 ps.

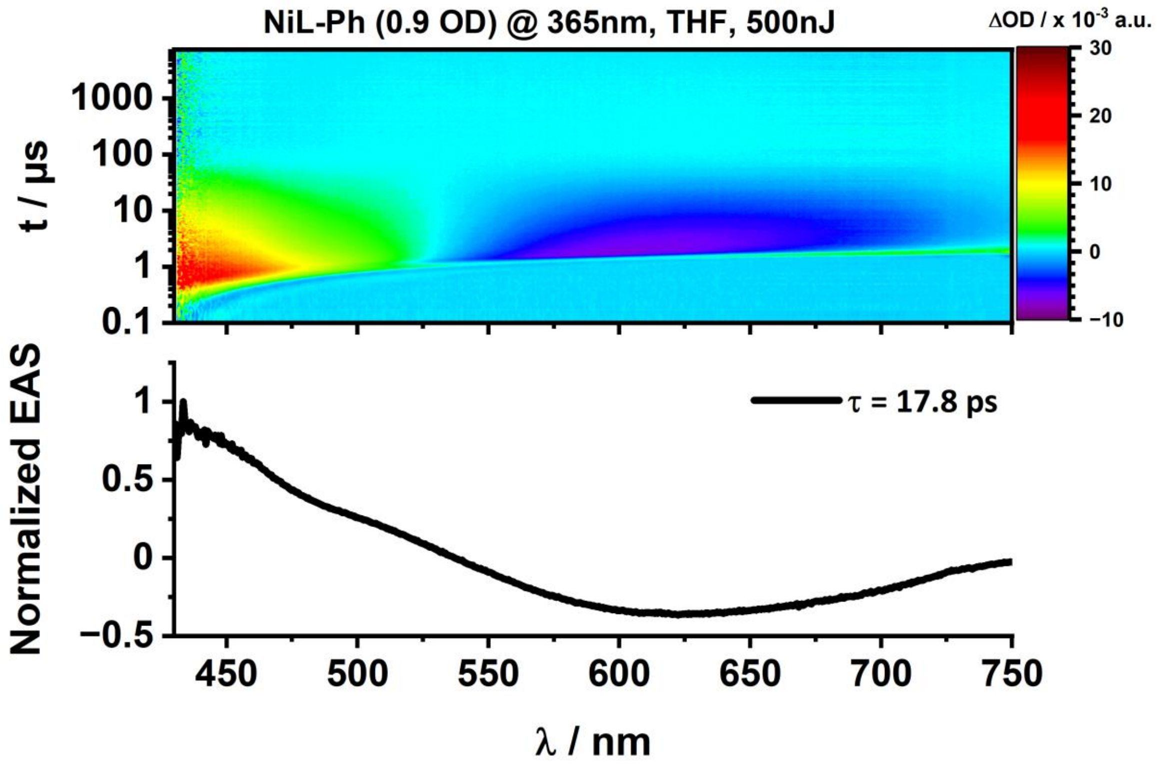

Figure S62. 3D heat map of [Ni(L^{NHPh})Cl] at 298 K obtained by cryostat supported fs-TAS measurements in THF (top). Evolution associated spectrum (bottom) of the fs-TA data of [Ni(L^{NHPh})Cl] with a lifetime of 17.8 ps.

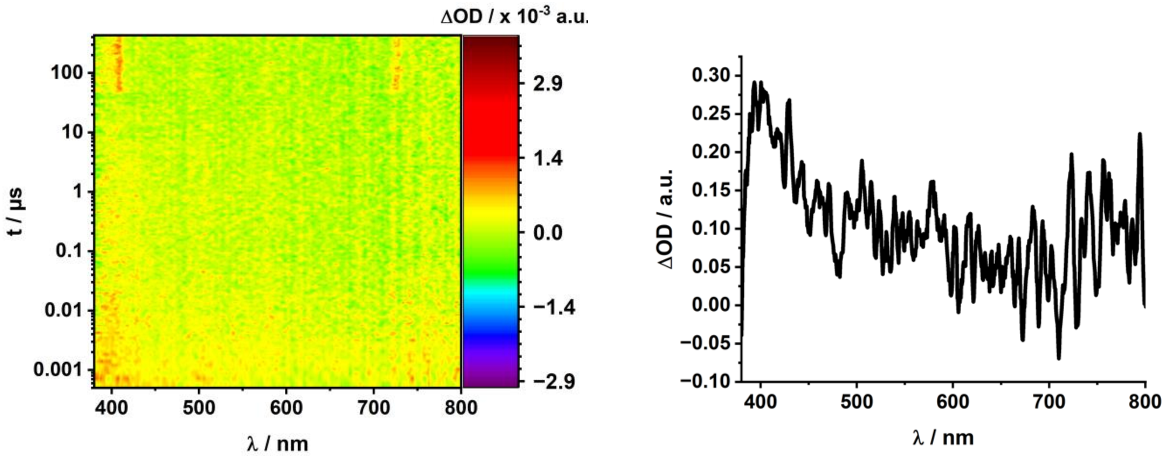

Figure S63. 3D heat map (left) of [Ni(L^{NHPh})Cl] at 298 K obtained by cryostat supported ns-TAS measurements in 2MeTHF. Evolution associated spectrum (right) of the ns-TA data of [Ni(L^{NHPh})Cl] with a lifetime of 722 ps.

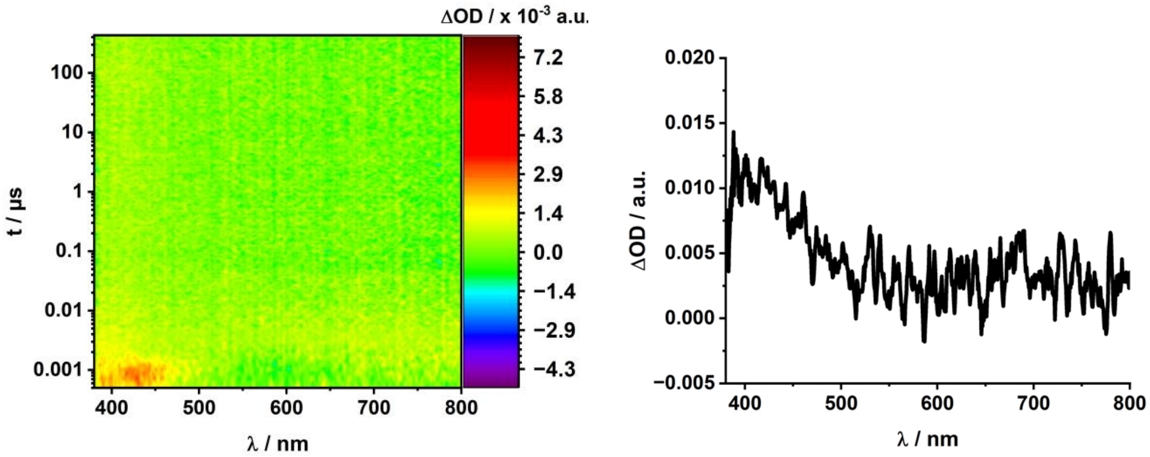

Figure S64. 3D heat map (left) of [Ni(L^{NHPh})Cl] at 150 K obtained by cryostat supported ns-TAS measurements in 2MeTHF. Evolution associated spectrum (right) of the ns-TA data of [Ni(L^{NHPh})Cl] with a lifetime of 10.1 ns.

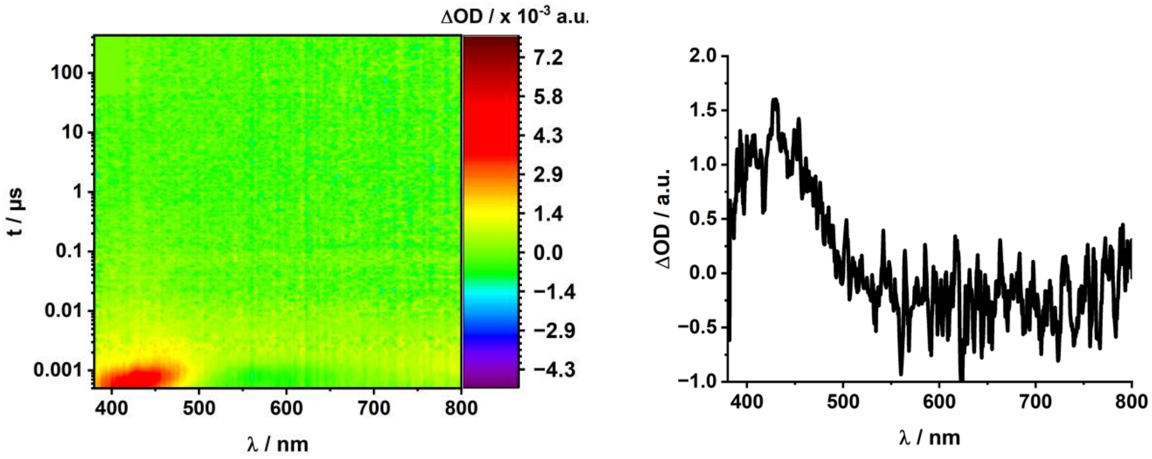

Figure S65. 3D heat map (left) of [Ni(L^{NHBn})Cl] at 298 K obtained by cryostat supported ns-TAS measurements in 2MeTHF. Evolution associated spectrum (right) of the ns-TA data of [Ni(L^{NHBn})Cl] with a lifetime of 1.94 ns.

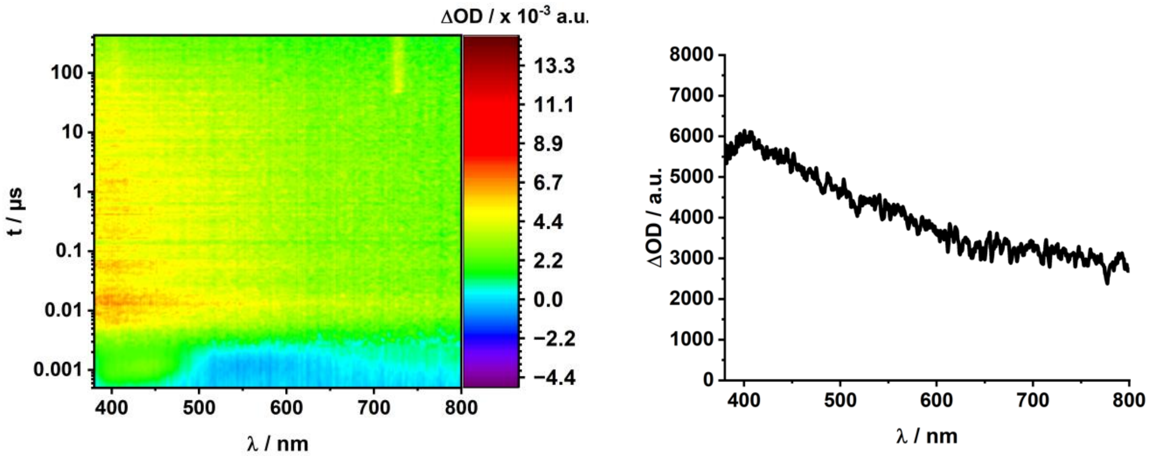

Figure S66. 3D heat map (left) of [Ni(L^{NHBn})Cl] at 180 K obtained by cryostat supported ns-TAS measurements in 2MeTHF. Evolution associated spectrum (right) of the ns-TA data of [Ni(L^{NHBn})Cl] with a lifetime of 500 fs. The fit was not successful because of the high intensity of the instrument response function (IRF).

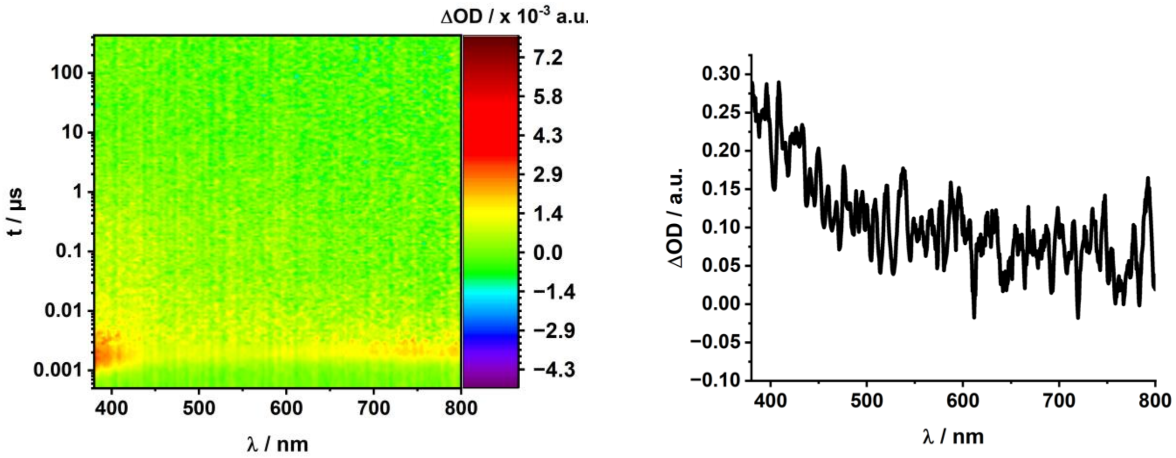

Figure S67. 3D heat map (left) of ClL^{NHPh} at 298 K obtained by ns-TAS measurements in 2MeTHF. Evolution associated spectrum (right) of the ns-TA data of ClL^{NHPh} with a lifetime of 8.3 ns.

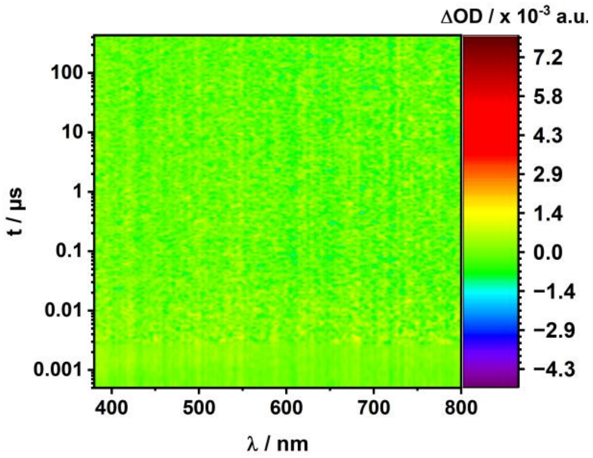

Figure S68. 3D heat map of ClL^{NHBn} at 298 K obtained by ns-TAS measurements in 2MeTHF. A fit was not possible due to the lack of signal.

Supporting Tables

Table S1. Crystal data and structure refinement for [Ni(N^C^N)Cl] (N^C^N = L^{NHPh} and L^{NHBn}).[a]

	[Ni(L^{NHPh})Cl]	[Ni(L^{NHBn})Cl]
Empirical formula	$C_{28}H_{21}ClN_4Ni$	$C_{30}H_{25}ClN_4Ni$
Formula weight	507.652	535.706
Temperature/K	100(2)	100(2)
Crystal system	monoclinic	monoclinic
Space group	$P2_1/n$	$P2_1/n$
a/Å	16.5402(2)	16.7842(1)
b/Å	8.0937(8)	26.3802(2)
c/Å	17.2587(2)	18.1847(1)
α/°	90	90
β/°	105.007(3)	116.691(3)
γ/°	90	90
Volume/$Å^3$	2231.6(4)	7193.7(9)
Z	4	12
ρ_{calc} g/cm^3	1.511	1.484
μ/mm^{-1}	1.015	0.949
F(000)	1048.0	3336.0
Crystal size/mm^3	0.1 × 0.02 × 0.02	0.4 × 0.4 × 0.2
2Θ range for data collection/°	4.88 to 53.464	3.978 to 54.202
Index ranges	$-20 \le h \le 20$, $-10 \le k \le 10$, $-21 \le l \le 21$	$-21 \le h \le 21$, $-33 \le k \le 33$, $-23 \le l \le 23$
Reflections collected	58685	193511
Independent reflections	4729 [R_{int} = 0.1310, R_{sigma} = 0.0566]	15873 [R_{int} = 0.0967, R_{sigma} = 0.0396]
Data/restraints/parameters	4729/0/307	15873/0/973
Goodness-of-fit on F^2	1.025	1.107
Final R indexes [I>=2σ (I)]	R_1 = 0.0468, wR_2 = 0.0988	R_1 = 0.0536, wR_2 = 0.1292
Final R indexes [all data]	R_1 = 0.0783, wR_2 = 0.1166	R_1 = 0.0674, wR_2 = 0.1388
Largest diff. peak/hole / e $Å^{-3}$	0.57/–0.64	2.06/–0.96
CCDC	2246584	2252984

[a] From X-ray diffraction on single crystals, measured using MoKα (λ = 0.71073) radiation.

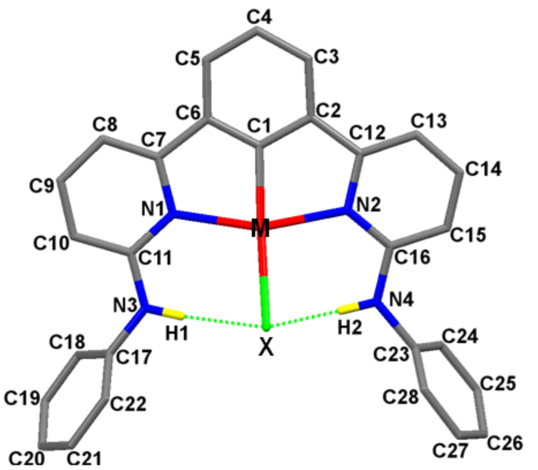

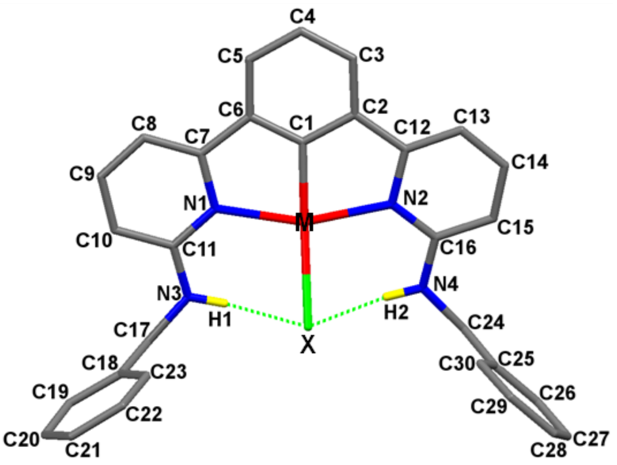

Numbering schemes of the [Ni(N^C^N)X] (X = Cl, F, or H) structures for Tables S2 to S4.

Table S2. Experimental and DFT-calculated geometries of the [Ni(N^C^N)Cl] (N^C^N = L^{NHPh} or L^{NHBn}) and [Ni(dpb)Cl] complexes.[a]

	[Ni(L^{NHPh})Cl]			[Ni(L^{NHBn})Cl]			[Ni(dpb)Cl]		
	XRD	DFT [a]		XRD [b]	DFT [a]		XRD [c]	DFT [a]	
bond lengths / Å		S_0	T_1		S_0	T_1		S_0	T_1
M–C1	1.821(4)	1.819	1.930	1.823(3)	1.820	1.932	1.836(7)	1.831	1.951
M–N1	1.987(3)	1.985	2.175	1.990(3)	1.992	2.191	1.932(6)	1.946	2.184
M–N2	1.980(3)	1.985	2.175	1.995(3)	1.993	2.183	1.936(5)	1.946	2.184
M–Cl	2.313(1)	2.397	2.352	2.278(3)	2.379	2.351	2.247(2)	2.292	2.329
Cl···H1	2.418(9)	2.147	2.366	2.479(9)	2.169	2.378	–	–	–
Cl···H2	2.358(8)	2.147	2.366	2.480(9)	2.174	2.414	–	–	–
angles / °									
C1–M–Cl	151.4(1)	149.1	130.2	154.9(1)	152.0	135.9	179.7(2)	179.9	175.7
C1–M–N1	82.0(1)	82.4	79.9	81.9(1)	82.2	79.6	81.6(3)	82.3	78.9
C1–M–N2	82.2(1)	82.4	79.9	82.1(1)	82.3	79.9	82.3(3)	82.3	78.9
N1–M–N2	161.2(1)	161.2	158.8	161.2(9)	161.2	158.4	163.9(2)	164.6	157.7
N1–M–Cl	98.2(9)	99.4	98.8	99.1(7)	99.2	99.7	98.2(2)	97.7	101.1
N2–M–Cl	100.6(1)	99.4	98.8	99.6(7)	99.5	99.7	97.9(2)	97.7	101.1
N3–H1···Cl	130.9(2)	158.8	159.9	125.2(4)	154.0	159.0	–	–	–
N4–H2···Cl	137.8(2)	158.8	159.9	124.3(4)	154.0	155.2	–	–	–
M–N1–C7–C6	6.5(3)	4.5	1.4	6.8(4)	4.7	1.4	0.4(7)	0.0	–0.04
M–N2–C12–C2	–5.1(3)	–4.5	–1.4	–4.9(4)	–3.5	–1.8	–1.5(7)	0.0	0.04
M–C1–C6–C7	–14.1(4)	–11.8	0.5	–10.0	–11.9	–0.2	–0.1(8)	0.001	–1.6
M–C1–C2–C12	10.0(4)	11.8	–0.5	9.9(4)	11.7	0.2	–0.0(8)	–0.001	1.6
τ_4	0.336	0.352	0.501	0.311	0.332	0.466	0.116	0.111	0.189

[a] DFT calculations on TPSSh/6-31G(d)/LanL2DZ ecp/CPCM(THF) level of theory. [b] Averaged values from three independent molecules in the unit cell. [c] From Ref. 3.

Table S3. Selected experimental structural data for [Ni(L^{NHBn})Cl].[a]

	XRD [1]	XRD [2]	XRD [3]
bond lengths / Å			
M–C1	1.821(3)	1.826(3)	1.821(3)
M–N1	1.987(3)	2.005(3)	1.978(3)
M–N2	1.990(3)	1.996(3)	1.999(3)
M–Cl	2.278(8)	2.280(8)	2.277(7)

Cl···H1	2.470(9)	2.515(9)	2.453(9)
Cl···H2	2.496(9)	2.457(9)	2.488(9)
angles / °			
C1–M–Cl	154.1(1)	155.2(1)	155.5(1)
C1–M–N1	81.9(1)	81.8(1)	82.1(1)
C1–M–N2	82.2(1)	82.1(1)	82.1(1)
N1–M–N2	161.0(9)	161.2(9)	161.5(9)
N1–M–Cl	98.8(7)	100.0(7)	98.6(7)
N2–M–Cl	100.1(7)	98.8(7)	99.8(7)
N3–H1···Cl	125.9(4)	122.7(4)	127.0(4)
N4–H2···Cl	122.8(4)	126.3(4)	123.9(4)
M–N1–C7–C6	6.2(4)	5.7(4)	8.7(4)
M–N2–C12–C2	–4.1(4)	–5.2(4)	–5.5(4)
M–C1–C6–C7	–11.9(4)	–7.6(4)	–10.5(4)
M–C1–C2–C12	9.5(3)	12.1(4)	8.2(4)
τ_4	0.318	0.309	0.305

[a] Three independent molecules in the unit cell.

Table S4. DFT-calculated geometries of the [Ni(N^C^N)Cl] (N^C^N = L^{NHPh} or L^{NHBn}).

	[Ni(L^{NHPh})Cl]				[Ni(L^{NHBn})Cl]			
	XRD	DFT [a]			XRD	DFT [a]		
bond lengths / Å		S_0 [a]	S_0 [b]	S_0 [c]		S_0	S_0 [b]	S_0 [d]
M–C1	1.821(4)	1.819	1.812	1.828	1.823(3)	1.820	1.812	1.817
M–N1	1.987(3)	1.985	1.992	2.032	1.990(3)	1.992	1.987	1.992
M–N2	1.980(3)	1.985	1.988	2.034	1.995(3)	1.993	1.988	1.991
M–Cl	2.313(1)	2.397	2.378	2.365	2.278(3)	2.379	2.363	2.365
Cl···H1	2.418(9)	2.147	2.188	2.135	2.479(9)	2.169	2.143	2.178
Cl···H2	2.358(8)	2.147	2.096	2.217	2.480(9)	2.174	2.152	2.168
angles / °								
C1–M–Cl	151.4(1)	149.1	153.2	154.3	154.9(1)	152.0	153.4	152.6
C1–M–N1	82.0(1)	82.4	82.2	81.9	81.9(1)	82.2	82.3	82.2
C1–M–N2	82.2(1)	82.4	82.6	82.0	82.1(1)	82.3	82.4	82.1
N1–M–N2	161.2(1)	161.2	160.6	160.4	161.2(9)	161.2	160.7	160.8
N1–M–Cl	98.2(9)	99.4	99.8	99.4	99.1(7)	99.2	99.5	99.6
N2–M–Cl	100.6(1)	99.4	98.6	100.0	99.6(7)	99.5	99.7	99.5
N3–H1···Cl	130.9(2)	158.8	143.5	160.0	125.2(4)	154.0	153.0	152.5
N4–H2···Cl	137.8(2)	158.8	158.7	146.4	124.3(4)	154.0	152.5	153.2
M–N1–C7–C6	6.5(3)	4.5	2.7	–5.7	6.8(4)	4.7	4.8	–4.0
M–N2–C12–C2	–5.1(3)	–4.5	–5.9	2.5	–4.9(4)	–3.5	–3.2	4.9
M–C1–C6–C7	–14.1(4)	–11.8	0.5	11.8	–10.0	–11.9	–11.8	12.2
M–C1–C2–C12	10.0(4)	11.8	–11.6	–11.4	9.9(4)	11.7	11.6	–12.2
τ_4	0.336	0.352	0.327	0.321	0.311	0.332	0.326	0.330

[a] DFT calculations on TPSSh/6-31G(d)/LanL2DZ ecp/CPCM(THF) level of theory. [b] DFT calculations on TPSSh/def2-TZVP/CPCM(THF) level of theory. [c] DFT calculations on B3LYP/6-31G(d)/def2-TZVP/CPCM(THF) level of theory. [d] DFT calculations on TPSSh/6-31G(d)/def2-TZVP/CPCM(THF) level of theory.

Table S5. Selected DFT-calculated geometries of the [Ni(N^C^N)X] Complexes in the S_0 ground state.[a]

	[Ni(L^{NHPh})F]	[Ni(L^{NHPh})H]	[Ni(L^{OMe})Cl]
bond lengths / Å	S_0	S_0	S_0
M–C1	1.817	1.843	1.822
M–N1	1.977	1.970	1.948
M–N2	1.977	1.970	1.948
M–X	1.903	1.590	2.360
X···H1	1.571	1.511	–
X···H2	1.571	1.511	–
angles / °			
C1–M–X	168.7	180.0	141.2
C1–M–N1	82.7	82.2	82.0
C1–M–N2	82.7	82.2	82.0
N1–M–N2	164.4	164.3	160.9
N1–M– X	97.7	97.8	99.5
N2–M– X	97.7	97.8	99.5
N3–H1···X	162.3	157.7	–
N4–H2···X	162.3	157.7	–
M–N1–C7–C6	3.3	2.1	7.8
M–N2–C12–C2	–3.3	2.1	–7.8
M–C1–C6–C7	–6.0	–0.2	–12.0
M–C1–C2–C12	6.0	–0.2	12.0
τ_4	0.190	0.111	0.411

[a] DFT calculations on TPSSh/6-31G(d)/LanL2DZ ecp/CPCM(THF) level of theory.

Table S6. Redox potentials of the complexes [Ni(N^C^N)Cl].[a]

complex	E_{pc} (Red3)	E_{pc} (Red2)	E_{pc} (Red1)	E_{pa} (Ox1)	E_{pa} (Ox2)	E_{pa} (Ox3)	ΔE (Red1–Red2)	ΔE (Ox1–Red1)
[Ni(L^{NHPh})Cl]	–2.94 [b]	–2.63	–2.17	0.06	1.02	–	0.46	2.23
[Ni(L^{NHBn})Cl]	–3.30	–2.77	–2.38	0.13	0.81	1.20	0.39	2.51

[a] From cyclic voltammetry in *n*-Bu_4NPF_6/THF. Potentials in V vs. ferrocene/ferrocenium; cathodic peak potentials (E_{pc}) for irreversible reductions; anodic peak potentials (E_{pa}) for irreversible oxidations.

Table S7. DFT-calculated energies and compositions of frontier MOs in the S_0 ground state for [Ni(L^{NHPh})Cl].[a]

MO	energy (eV)	component %			
		Ni	N^C^N = L	NHPh = L′	Cl = X
LUMO+12	0.974	35	53	12	0
LUMO+11	0.965	79	9	8	4
LUMO+10	0.768	79	13	6	2
LUMO+9	0.086	7	48	44	1
LUMO+8	–0.114	3	78	19	0
LUMO+7	–0.174	0	32	68	0
LUMO+6	–0.479	0	6	94	0
LUMO+5	–0.493	2	6	92	0
LUMO+4	–0.915	1	60	39	0
LUMO+3	–1.126	1	73	26	0
LUMO+2	–1.928	26	67	4	3
LUMO+1	–2.029	1	97	2	0
LUMO	–2.111	35	60	3	2
HOMO	–5.170	38	29	27	6
HOMO-1	–5.246	14	31	54	1

HOMO-2	–5.383	84	10	4	2
HOMO-3	–5.699	38	24	36	2
HOMO-4	–5.722	70	25	2	3
HOMO-5	–6.332	9	53	35	3
HOMO-6	–6.591	20	41	32	7
HOMO-7	–6.630	46	7	27	20
HOMO-8	–6.643	31	5	51	13
HOMO-9	–6.654	9	19	69	3
HOMO-10	–7.072	4	42	35	19
HOMO-11	–7.310	6	21	9	64
HOMO-12	–7.418	7	49	44	0

[a] LUMO: Lowest Unoccupied Molecular Orbital, and HOMO: Highest Occupied Molecular Orbital. Major contributions (>30%) are marked in grey.

Table S8. DFT-calculated energies and compositions of frontier MOs in the T_1 excited state for [Ni(L^{NHPh})Cl].[a]

MO	energy (eV)	component %			
		Ni	N^C^N = L	NHPh = L′	Cl = X
HSOMO+3	–1.047	0	73	27	0
HSOMO+2	–1.827	7	87	5	1
HSOMO+1	–1.872	0	98	2	0
HSOMO	–4.825	31	66	1	2
LSOMO	–5.241	1	39	60	0
LSOMO-1	–5.297	2	34	63	1
LSOMO-2	–6.061	9	75	15	1
LSOMO-3	–6.134	1	73	26	0

[a] LSOMO: Lowest Singly Occupied Molecular Orbital, and HSOMO: Highest Singly Occupied Molecular Orbital. Major contributions (>30%) are marked in grey.

Table S9. DFT-calculated energies and compositions of frontier MOs in the S_0 ground state for [Ni(L^{NHBn})Cl].[a]

MO	energy (eV)	component %			
		Ni	N^C^N = L	NHBn = L′	Cl = X
LUMO+12	1.005	7	84	9	0
LUMO+11	0.884	77	8	11	4
LUMO+10	0.711	72	14	13	1
LUMO+9	0.030	5	91	4	0
LUMO+8	–0.355	1	33	66	0
LUMO+7	–0.397	1	18	81	0
LUMO+6	–0.422	1	6	93	0
LUMO+5	–0.445	2	9	89	0
LUMO+4	–0.468	1	37	62	0
LUMO+3	–0.803	1	82	17	0
LUMO+2	–1.836	23	71	3	3
LUMO+1	–1.942	2	97	1	0
LUMO	–2.025	38	58	2	2
HOMO	–5.201	59	25	8	8
HOMO-1	–5.324	75	18	6	1
HOMO-2	–5.338	40	30	26	4
HOMO-3	–5.676	52	43	4	1
HOMO-4	–5.909	24	37	38	1
HOMO-5	–6.348	2	6	88	4

HOMO-6	–6.358	5	7	86	2
HOMO-7	–6.504	24	5	61	10
HOMO-8	–6.533	46	7	27	20
HOMO-9	–6.544	2	2	95	1
HOMO-10	–6.660	20	56	13	11
HOMO-11	–6.784	12	52	30	6
HOMO-12	–7.206	5	19	6	70

[a] LUMO: Lowest Unoccupied Molecular Orbital, and HOMO: Highest Occupied Molecular Orbital. Major contributions (>30%) are marked in grey.

Table S10. DFT-calculated energies and compositions of frontier MOs in the T_1 excited state for [Ni(L^{NHBn})Cl].[a]

MO	energy (eV)	component %			
		Ni	N^C^N = L	NHBn = L′	Cl = X
HSOMO+3	–0.709	1	80	19	0
HSOMO+2	–1.727	8	87	4	1
HSOMO+1	–1.776	0	98	1	0
HSOMO	–4.783	31	66	1	2
LSOMO	–5.388	0	62	36	1
LSOMO-1	–5.511	3	58	38	2
LSOMO-2	–6.135	7	63	30	1
LSOMO-3	–6.258	3	36	60	1

[a] LSOMO: Lowest Singly Occupied Molecular Orbital, and HSOMO: Highest Singly Occupied Molecular Orbital. Major contributions (>30%) are marked in grey.

Table S11. Selected experimental UV-vis absorption maxima of the complexes [Ni(N^C^N)Cl].[a]

Complex	[Ni(L^{NHPh})Cl]	[Ni(L^{NHBn})Cl]
λ_3	283	250
λ_2	368	340
λ_1	427	423

[a] Measured in THF at 298 K, absorption maxima λ in nm.

Table S12. Selected TD-DFT-calculated vertical $S_0 \rightarrow S_n$ transitions for [Ni(L^{NHPh})Cl].[a]

S_n	osc. strength f_{osc}	λ (nm)	transitions (major contribution)	assignment [b]
S_1	0.004	555.6	H-2→LUMO (37%)	MLCT, d–d
			HOMO→LUMO (37%)	MLCT, d–d, π–π* (L), LMCT, L′LCT, L′MCT
			HOMO→L+2 (16%)	MLCT, d–d, π–π* (L), LMCT, L′LCT, L′MCT
S_3	0.000	524.9	H-4→LUMO (37%)	MLCT, d–d, π–π* (L), LMCT
			H-1→LUMO (32%)	L′LCT, L′MCT, LLCT, LMCT
			H-4→L+2 (17%)	MLCT, d–d, π–π* (L), LMCT
S_4	0.042	438.7	HOMO→L+1 (77%)	MLCT, LLCT, L′LCT
			H-2→L+1 (18%)	MLCT
S_6	0.033	429.2	H-1→LUMO (61%)	L′LCT, L′MCT, π–π* (L), LMCT
			H-4→LUMO (13%)	MLCT, d–d, π–π* (L), LMCT
S_9	0.130	410.7	H-1→L+2 (63%)	L′LCT, L′MCT, π–π* (L), LMCT
			H-4→LUMO (18%)	MLCT, d–d, π–π* (L), LMCT
S_{16}	0.081	355.4	H-3→L+2 (44%)	MLCT, d–d, L′LCT, L′MCT, π–π* (L), LMCT
			H-4→L+1 (41%)	MLCT, π–π* (L)
S_{22}	0.292	307.7	HOMO→L+4 (44%)	MLCT, ML′CT, π–π* (L), LL′CT, L′LCT, π–π* (L′)
			H-1→L+3 (26%)	L′LCT, π–π* (L′), π–π* (L), LL′CT

			H-2→L+4 (23%)	MLCT, ML′CT
S_{23}	0.118	293.0	H-5→L+1 (35%)	π–π* (L), L′LCT
			H-8→L+1 (23%)	L′LCT, MLCT
			H-4→L+3 (22%)	MLCT, ML′CT, π–π* (L), LL′CT
			H-7→L+1 (13%)	MLCT, L′LCT, XLCT
S_{43}	0.260	272.0	H-3→L+4 (77%)	MLCT, ML′CT, L′LCT, π–π* (L′), π–π* (L), LL′CT

[a] For oscillator strength f > 0.01 except for the lowest-energy transition. M = Ni; L = N^C^N core; L′ = NHPh; X = Cl. [b] Sorted from highest to lowest probability.

Table S13. Selected TD-DFT-calculated vertical $S_0 \rightarrow S_n$ transitions for [Ni(L^{NHBn})Cl].[a]

S_n	osc. strength f_{osc}	λ (nm)	transitions (major contribution)	assignment [b]
S_1	0.003	547.3	HOMO→LUMO (50%)	MLCT, d–d, π–π* (L), LMCT
			H-1→LUMO (25%)	MLCT, d–d, π–π* (L), LMCT
			HOMO→L+2 (17%)	MLCT, d–d
S_2	0.001	523.8	H-2→LUMO (34%)	MLCT, d–d, π–π* (L), LMCT, L′LCT, L′MCT
			H-3→LUMO (25%)	MLCT, d–d, π–π* (L), LMCT
			H-2→L+2 (12%)	MLCT, d–d, π–π* (L), LMCT, L′LCT, L′MCT
S_4	0.015	430.7	HOMO→L+1 (44%)	MLCT, LLCT
			HOMO→L+2 (15%)	MLCT, d–d, π–π* (L), LMCT
			H-1→L+1 (11%)	MLCT
S_9	0.220	397.6	H-2→LUMO (22%)	MLCT, d–d, π–π* (L), LMCT, L′LCT, L′MCT
			H-2→L+2 (18%)	MLCT, d–d, π–π* (L), LMCT, L′LCT, L′MCT
S_{14}	0.056	346.0	H-3→L+1 (77%)	MLCT, π–π* (L)
S_{16}	0.087	326.0	H-4→L+2 (74%)	L′LCT, L′MCT, LLCT, LMCT, MLCT, d–d
S_{31}	0.065	285.3	H-2→L+3 (61%)	MLCT, π–π* (L), L′LCT
S_{44}	0.041	268.8	H-2→L+4 (39%)	ML′CT, MLCT, LL′CT, π–π* (L), π–π* (L′), L′LCT
			H-11→L+2 (20%)	π–π* (L), LMCT, L′LCT, L′MCT

[a] For oscillator strength f > 0.01 except for the lowest-energy transition. M = Ni; L = N^C^N core; L′ = NHBn; X = Cl. [b] Sorted from highest to lowest probability.

Table S14. Excited state lifetimes of the ligand precursors ClL^{NHPh} and ClL^{NHBn} and the protonated ligands HL^{NHPh}, and HL^{NHBn} [a]

	ClL^{NHPh}	ClL^{NHBn}	HL^{NHPh}	HL^{NHBn}
Fluorescence lifetime (ns)	τ_1 = 6.9 ± 0.1 (18%)<br>τ_2 = 3.31 ± 0.03 (82%)<br>τ_{av_amp} = 3.96 ± 0.02	τ_1 = 6.0 ± 0.5 (29%)<br>τ_2 = 3.5 ± 0.2 (71%)<br>τ_{av_amp} = 4.21 ± 0.05	τ = 7.85 ± 0.02	τ = 7.55 ± 0.02
Phosphorescence lifetime (ms)	τ_1 = 289 ± 6 (39%)<br>τ_2 = 144 ± 3 (61%)<br>τ_{av_amp} = 200 ± 1	τ_1 = 215 ± 6 (30%)<br>τ_2 = 75 ± 2 (70%)<br>τ_{av_amp} = 117 ± 1	n.d.	n.d.

[a] From both fluorescence (ns lifetimes) and phosphorescence (ms lifetimes) spectra in a frozen glassy matrix of 2Me-THF at 77 K. For multiexponential decays, the amplitude-weighted average lifetimes are indicated as well as the different components with their relative amplitudes.

Table S15. Characterization of selected TDA-calculated vertical excited states for the optimized ground state geometry of [Ni(L^{NHPh})Cl] using PBE0/SDD. Only orbital transitions with a weight of at least 10% are listed.

state	osc. strength f_{osc}	λ (nm)	Orbital transitions (major contribution)
S_1	0.002	604.7	HOMO-2→LUMO+3 (70%)
			HOMO→LUMO+3 (14%)

S_4	0.001	464.7	HOMO-13→LUMO+3 (48%)
			HOMO-9→LUMO+3 (35%)
T_5	0.000	455.7	HOMO→LUMO+1 (45%)
			HOMO-1→LUMO (33%)

Table S16. Characterization of selected TDA-calculated excited states for the optimized ground state geometry of [Ni(L^{NHBn})Cl] using PBE0/SDD. Only orbital transitions with a weight of at least 10% are listed.

state	osc. strength f_{osc}	λ (nm)	transitions (major contribution)
S_1	0.003	597.0	HOMO-2→LUMO+2 (60%)
			HOMO→LUMO+2 (20%)
S_4	0.001	466.5	HOMO-14→LUMO+2 (48%)
			HOMO-8→LUMO+2 (29%)
T_5	0.000	446.5	HOMO→LUMO+1 (44%)
			HOMO-1→LUMO (34%)
			HOMO-3→LUMO (10%)

Table S17. Characterization of selected TDA-calculated excited states for the optimized ground state geometry of [Ni(L^{NHPh})Cl] using PBE0/ZORA-DEF2-TZVP. Only orbital transitions with a weight of at least 10% are listed.

state	osc. strength f_{osc}	λ (nm)	transitions (major contribution)
S_1	0.001	582.6	HOMO-2→LUMO+4 (74%)
			HOMO→LUMO+4 (11%)
S_4	0.001	461.4	HOMO-9→LUMO+4 (48%)
			HOMO-13→LUMO+4 (41%)
T_5	0.000	480.4	HOMO→LUMO+1 (42%)
			HOMO-1→LUMO (37%)

Table S18. Characterization of selected TDA-calculated excited states for the optimized ground state geometry of [Ni(L^{NHBn})Cl] using PBE0/ZORA-DEF2-TZVP. Only orbital transitions with a weight of at least 10% are listed.

state	osc. strength f_{osc}	λ (nm)	transitions (major contribution)
S_1	0.001	575.6	HOMO-2→LUMO+2 (66%)
			HOMO→LUMO+2 (15%)
S_4	0.001	463.3	HOMO-8→LUMO+2 (43%)
			HOMO-13→LUMO+2 (38%)
T_5	0.000	469.3	HOMO-1→LUMO (40%)
			HOMO-1→LUMO (42%)

Table S19. Radiative lifetimes from SOC-TDA calculations at the optimized ground state geometry of [Ni(L^{NHPh})Cl] in THF using PBE0/ZORA-DEF2-TZVP. The Boltzmann average at 77 K is given in parentheses in bold.

state	osc. strength f_{osc} (x10^5)	λ (nm)	Radiative lifetime
S_1	225.934	570.2	1.085 μs
S_4	124.957	458.2	1.267 μs
T_5	0.016 / 0.370 / 0.161	478.7 / 478.7 / 478.7	10.600 ms / 466.374 μs / 1.073 ms / **(946.286 μs)**

Table S20. Radiative lifetimes from SOC-TDA calculations at the optimized ground state geometry of [Ni(L^{NHBn})Cl] in THF using PBE0/ZORA-DEF2-TZVP. The Boltzmann average at 77 K is given in parentheses in bold.

state	osc. strength f_{osc} (x10^5)	λ (nm)	Radiative lifetime
S_1	164.065	563.8	1.461 μs
S_4	128.187	460.4	1.247 μs
T_5	0.016 / 0.370 / 0.161	471.6 / 471.6 / 471.6	2.943 ms / 10.443 ms / 2.147 ms / (3.330 ms)

Table S21. Calculated coordinates of the optimized singlet ground state structures of the complexes [Ni(N^C^N)Cl].[a]

[Ni(L^{NHPh})Cl]				[Ni(L^{NHBn})Cl]			
atom	coordinates (Å)			atom	coordinates (Å)		
	X	Y	Z		X	Y	Z
Cl	0.000041	0.712197	1.998640	Cl	–0.45660	–0.96514	–1.65564
C	1.224141	–3.43538	–0.14768	N	–1.85940	1.096781	0.576145
N	–1.95889	–1.20829	0.072724	N	1.892379	1.157801	–0.59799
C	–0.00021	–2.76326	–0.11329	N	–2.28181	–1.18276	0.872175
N	–2.44217	1.069396	0.082245	H	–1.52474	–1.33093	0.202593
H	–1.56419	1.171992	0.601774	N	2.475371	–1.10496	–0.59938
N	1.958713	–1.20856	0.072656	H	1.473272	–1.26718	–0.71294
C	2.361450	–2.51826	–0.08997	C	0.024880	2.686894	0.091704
N	2.442335	1.069051	0.082307	C	1.179787	3.380893	–0.27823
H	1.564416	1.171746	0.601917	C	1.164878	4.785776	–0.24930
C	2.896165	–0.22240	–0.00142	H	2.042551	5.362907	–0.52885
C	–1.22465	–3.43522	–0.14759	C	–0.00528	5.451608	0.139735
C	–2.89620	–0.22199	–0.00135	H	–0.01674	6.537298	0.160303
C	–3.69619	–2.87160	–0.24277	C	–1.16334	4.747134	0.495557
H	–3.97212	–3.91182	–0.36959	H	–2.05723	5.294783	0.782970
C	–2.36183	–2.51794	–0.08983	C	–1.15066	3.342236	0.466060
C	4.522571	4.691836	–0.52737	C	–2.22400	2.408421	0.802663
H	5.043290	5.632019	–0.6828	C	–3.45188	2.760615	1.347240
C	4.737142	3.614196	–1.39288	H	–3.6982	3.804381	1.503208
H	5.418387	3.716771	–2.23301	C	–4.33938	1.740578	1.711657
C	–1.22329	–4.83685	–0.24862	H	–5.30981	1.982712	2.134413
H	–2.15299	–5.39911	–0.28027	C	–3.97263	0.418833	1.543466
C	4.065430	2.404276	–1.20547	H	–4.64621	–0.38260	1.818300
H	4.207390	1.588883	–1.90803	C	–2.70715	0.106092	0.981626
C	–4.06519	2.404753	–1.20549	C	2.277608	2.482256	–0.63098
H	–4.20737	1.58931	–1.90794	C	3.580154	2.874181	–0.91204
C	–3.18262	2.253322	–0.12166	H	3.838113	3.926480	–0.93644
C	4.269379	–0.53552	–0.14025	C	4.546455	1.883312	–1.12374
H	4.997352	0.265821	–0.13215	H	5.574266	2.157512	–1.34165
C	–4.52186	4.692465	–0.52758	C	4.194779	0.549464	–1.02985
H	–5.04244	5.632718	–0.68305	H	4.935568	–0.23092	–1.15044
C	3.695757	–2.87211	–0.24303	C	2.848824	0.197014	–0.75386
H	3.971524	–3.91236	–0.36992	C	–3.12644	–2.33582	1.162431
C	4.660817	–1.8576	–0.24758	H	–3.48422	–2.26918	2.196473
H	5.714531	–2.10301	–0.33813	H	–2.46163	–3.20527	1.122061

C	–2.96017	3.338686	0.743456	C	–4.30783	–2.55096	0.220891
H	–2.27159	3.224366	1.576352	C	-4.22686	–2.21504	–1.13802
C	–3.62210	4.549408	0.533994	H	–3.31876	–1.75902	–1.52566
H	–3.43947	5.379281	1.210983	C	–5.3057	–2.46097	–1.99263
C	–4.66111	–1.85695	–0.24728	H	–5.23077	–2.19429	–3.04354
H	–5.71487	–2.10221	–0.33773	C	–6.47672	–3.04743	–1.50066
C	1.222591	–4.83701	–0.24871	H	–7.3148	–3.23597	–2.16600
H	2.152208	–5.3994	–0.28042	C	–6.56415	–3.38519	–0.14649
C	–4.73672	3.614766	–1.39295	H	–7.47174	–3.83552	0.246634
H	–5.41805	3.717364	–2.23301	C	–5.48606	–3.13495	0.708222
C	2.960807	3.338182	0.743708	H	–5.56138	–3.39451	1.762466
H	2.272301	3.223886	1.576675	C	3.336976	–2.22135	–0.97867
C	–0.00040	–5.51961	–0.30106	H	2.671498	–3.08319	–1.09550
H	–0.00048	–6.60279	–0.37707	H	3.799861	–2.04646	–1.96051
C	3.182966	2.252876	–0.12155	C	4.415174	–2.55353	0.044314
C	–4.26947	–0.53493	–0.14005	C	4.198478	–2.36728	1.416527
H	–4.99733	0.266524	–0.13193	H	3.259750	–1.93416	1.751750
C	3.622912	4.548815	0.534300	C	5.181136	–2.72461	2.344207
H	3.440507	5.378644	1.211402	H	5.001102	–2.57232	3.405141
Ni	–0.00008	–0.99603	0.317123	C	6.392385	–3.27512	1.911250
				H	7.156510	–3.55068	2.632880
				C	6.616045	–3.46312	0.543735
				H	7.556054	–3.88388	0.196904
				C	5.633433	–3.10111	–0.38302
				H	5.814861	–3.24411	–1.44645
				Ni	–0.04815	0.897169	–0.23221

[a] DFT calculations on TPSSh/6-31G(d)/LanL2DZ ecp/CPCM(THF) level of theory.

Table S22. Calculated coordinates of the optimized triplet excited state structures of the complexes [Ni(N^C^N)Cl].[a]

[Ni(L^{NHPh})Cl]				[Ni(L^{NHBn})Cl]			
atom	coordinates (Å)			atom	coordinates (Å)		
	X	Y	Z		X	Y	Z
Cl	0.000006	0.695938	1.923391	Cl	–0.42725	–1.07456	–1.54068
C	1.221909	–3.36993	–0.04951	N	–2.05665	0.980844	0.729041
N	–2.13773	–1.14957	–0.04047	N	2.055289	1.125918	–0.50779
C	0.000000	–2.69978	0.029811	N	–2.56938	–1.25279	1.051118
N	–2.74755	1.069715	0.053192	H	–1.77997	–1.42252	0.428387
H	–1.86453	1.191835	0.554037	N	2.788690	–1.06630	–0.60306
N	2.137733	–1.14957	–0.04048	H	1.799263	–1.27844	–0.73153
C	2.415894	–2.48871	–0.07264	C	–0.09966	2.590165	–0.04169
N	2.74755	1.069713	0.053194	C	1.058817	3.305897	–0.35089
H	1.864536	1.191828	0.554045	C	1.016997	4.712717	–0.33107
C	3.145003	–0.24801	–0.05387	H	1.890796	5.315707	–0.56636
C	–1.22191	–3.36993	–0.04950	C	–0.17960	5.355210	0.014893
C	–3.14500	–0.24801	–0.05386	H	–0.21362	6.441137	0.028477
C	–3.73038	–2.94810	–0.15446	C	–1.32555	4.630593	0.369832
H	–3.94112	–4.01058	–0.19004	H	–2.22182	5.171705	0.663782
C	–2.41589	–2.48871	–0.07263	C	–1.28068	3.223772	0.349788
C	4.977457	4.618338	–0.44921	C	–2.37468	2.311062	0.766566

H	5.538538	5.539244	–0.57690	C	–3.62798	2.743551	1.198764
C	5.212507	3.524748	–1.28875	H	–3.86823	3.799999	1.223455
H	5.951347	3.595622	–2.08229	C	-4.56769	1.787457	1.603846
C	–1.22392	–4.77397	–0.14893	H	–5.55006	2.101997	1.944408
H	–2.14814	–5.34369	–0.21071	C	–4.25683	0.437999	1.561408
C	4.490244	2.339076	–1.13722	H	-4.98364	–0.31137	1.849876
H	4.651791	1.513784	–1.82368	C	–2.97219	0.056066	1.104120
C	–4.49025	2.339067	–1.13723	C	2.257886	2.473475	–0.62040
H	–4.65181	1.513769	–1.82368	C	3.514500	2.992116	–0.93385
C	–3.53190	2.226671	–0.11414	H	3.662677	4.062008	–1.02253
C	4.495206	–0.65416	–0.13328	C	4.582944	2.105746	–1.11622
H	5.290366	0.080631	–0.11406	H	5.572549	2.488974	–1.34816
C	–4.97746	4.618335	–0.44923	C	4.385495	0.738122	–1.00656
H	–5.53854	5.539241	–0.57693	H	5.204410	0.041062	–1.13694
C	3.730375	–2.94810	–0.15449	C	3.082407	0.268479	–0.71514
H	3.941123	–4.01058	–0.19008	C	–3.47595	–2.37477	1.264743
C	4.77099	–2.01207	–0.18648	H	–3.92284	–2.28801	2.261925
H	5.802689	–2.34820	–0.23326	H	–2.84450	–3.26951	1.287476
C	–3.29065	3.328815	0.725604	C	–4.57670	–2.55002	0.222021
H	–2.54628	3.246910	1.513516	C	–4.35380	–2.24864	–1.12926
C	–4.00479	4.514723	0.551125	H	–3.39160	–1.84829	–1.44047
H	–3.80526	5.356466	1.208494	C	–5.36108	–2.45541	–2.07640
C	–4.77099	–2.01207	–0.18645	H	–5.17535	–2.2156	–3.12003
H	–5.80269	–2.34821	–0.23322	C	–6.60273	–2.96831	–1.68567
C	1.223922	–4.77398	–0.14894	H	–7.38531	–3.12627	–2.42272
H	2.148140	–5.34369	–0.21072	C	–6.83227	–3.27154	–0.34008
C	–5.21252	3.524738	–1.28876	H	–7.79546	–3.66432	–0.02531
H	–5.95136	3.595605	–2.08229	C	–5.82486	–3.06045	0.606678
C	3.290654	3.328807	0.725623	H	–6.01103	–3.29260	1.653558
H	2.546294	3.246895	1.513540	C	3.718693	–2.10947	–1.02283
C	–0.000001	–5.45663	–0.18318	H	3.113362	–3.00961	–1.17334
H	–0.000001	–6.54050	–0.25805	H	4.170926	–1.86803	–1.99601
C	3.531898	2.226671	–0.11413	C	4.816846	–2.40662	–0.01012
C	–4.49521	–0.65416	–0.13326	C	4.587499	–2.28469	1.367274
H	–5.29037	0.080631	–0.11404	H	3.621940	–1.92595	1.713963
C	4.004788	4.514716	0.551150	C	5.590094	–2.61091	2.285012
H	3.805263	5.356453	1.208529	H	5.399340	–2.50946	3.350185
Ni	0.000002	–0.77025	0.084035	C	6.834947	–3.06585	1.836674
				H	7.614475	–3.31763	2.550503
				C	7.071828	–3.18880	0.463945
				H	8.037603	–3.53486	0.105440
				C	6.068800	–2.85767	–0.45248
				H	6.260268	–2.94988	–1.51977
				Ni	–0.02570	0.660280	–0.00533

[a] DFT calculations on TPSSh/6-31G(d)/LanL2DZ ecp/CPCM(THF) level of theory.

References

1. Vogt, N. Sandleben, A.; Kletsch, L.; Schäfer, S.; Chin, M.T.; Vicic, D.A.; Hörner, G.; Klein, A. Role of the X Coligands in Cyclometalated [Ni(Phbpy)X] Complexes (HPhbpy = 6-Phenyl-2,2′-bipyridine), *Organometallics* **2021,** *40*, 1776−1785. https://doi.org/10.1021/acs.organomet.1c00237
2. Niazi, M.; Maisuls, I.; Strassert, C.A.; Klein, A. Molecular Rigidification of Cyclometalated N^C^N Pt(II)- and Pd(II)-based Triplet Emitters, *Organometallics* **2024**, *43*, 1547–1556. https://doi.org/10.1021/acs.organomet.4c00121
3. Kletsch, L.; Hörner, G. Klein, A. Cyclometalated Ni(II) Complexes [Ni(N∧C∧N)X] of the Tridentate 2,6-di(2-pyridyl)phen-ide Ligand, *Organometallics* **2020**, *39*, 2820–2829. https://doi.org/10.1021/acs.organomet.0c00355
